# Aggresome formation promotes ASK1/JNK signaling activation and stemness maintenance in ovarian cancer

**DOI:** 10.1038/s41467-024-45698-x

**Published:** 2024-02-13

**Authors:** Yurou Chen, Yulong Qiang, Jiachen Fan, Qian Zheng, Leilei Yan, Guanlan Fan, Xiaofei Song, Nan Zhang, Qiongying Lv, Jiaqiang Xiong, Jingtao Wang, Jing Cao, Yanyan Liu, Jie Xiong, Wei Zhang, Feng Li

**Affiliations:** 1 Department of Gynecology, Zhongnan Hospital of Wuhan University, Wuhan, 430071 China; 2 Department of Medical Genetics, TaiKang Medical School (School of Basic Medical Science), Wuhan University, Wuhan, 430071 China; 3 Department of Immunology, TaiKang Medical School (School of Basic Medical Science), Wuhan University, Wuhan, 430071 China; 4 Hubei Provincial Key Laboratory of Allergy and Immunology, Wuhan University, Wuhan, 430071 China

**Keywords:** Cancer stem cells, Cell signalling, Mechanisms of disease, Ovarian cancer

## Abstract

Aggresomes are the product of misfolded protein aggregation, and the presence of aggresomes has been correlated with poor prognosis in cancer patients. However, the exact role of aggresomes in tumorigenesis and cancer progression remains largely unknown. Herein, the multiomics screening reveal that OTUD1 protein plays an important role in retaining ovarian cancer stem cell (OCSC) properties. Mechanistically, the elevated OTUD1 protein levels lead to the formation of OTUD1-based cytoplasmic aggresomes, which is mediated by a short peptide located in the intrinsically disordered OTUD1 N-terminal region. Furthermore, OTUD1-based aggresomes recruit ASK1 via protein-protein interactions, which in turn stabilize ASK1 in a deubiquitinase-independent manner and activate the downstream JNK signaling pathway for OCSC maintenance. Notably, the disruption of OTUD1-based aggresomes or treatment with ASK1/JNK inhibitors, including ibrutinib, an FDA-approved drug that was recently identified as an MKK7 inhibitor, effectively reduced OCSC stemness (OSCS) of OTUD1^high^ ovarian cancer cells. In summary, our work suggests that aggresome formation in tumor cells could function as a signaling hub and that aggresome-based therapy has translational potential for patients with OTUD1^high^ ovarian cancer.

## Abstract (web summary)

The role of aggresomes in tumorigenesis and cancer progression remains to be explored. Here, the authors perform multi-omics and reveal that aggresome formation supports ovarian cancer stem cell properties via OTUD1 and ASK1/JNK signalling activation.

## Introduction

Epithelial ovarian cancer is the leading cause of gynecologic malignancy-associated death. High-grade serous ovarian cancer (HGSOC), which is the most common histotype in the clinic, is believed to arise from the fallopian tube^1^. Although initial treatment approaches such as the combination of optimal cytoreductive surgery and platinum-based chemotherapy are usually effective, approximately 80% of patients experience recurrence^2^, and treatment options for recurrent disease remain challenging. The presence of ovarian cancer stem cells (OCSCs) has historically been thought to play an important role in tumorigenicity, tumor metastasis and cancer recurrence after initial chemotherapy^3–8^.

The elimination of cancer stem cells (CSCs) is considered an effective strategy for cancer treatment, which prompted us to understand how the CSC subpopulation is maintained in tumors. Similar to normal stem cells, CSCs are capable of self-renewal and differentiation into heterogeneous cancer cells. The balance between self-renewal and differentiation contributes to the abundance of CSCs^9^. Several surface markers, such as CD44, CD133, CD24, ALDH and EpCAM, have been reported for the recognition or isolation of OCSCs^10–15^. Moreover, several transcription factors, including NANOG, OCT4 and SOX2, are assumed to serve as intracellular markers^16^. Whether there are alterations in the expression of certain genes that are essential for the transition of OCSCs to nontumorigenic cells is still largely unknown. Given that the ovarian CSC subpopulation can be maintained via different signaling pathways^9,17^, the elucidation of the mechanism underlying their activation would provide valuable diagnostic biomarkers and therapeutic targets.

The combination of traditional chemotherapy and OCSC-targeted therapy might be an effective and promising anticancer treatment approach for ovarian cancer. The targets of interest for anti-OCSC therapy are surface and intracellular markers of OCSCs that regulate OSCS maintenance. Activation of signaling pathways such as the WNT^18^, Hedgehog^19,20^, Notch^21^, JNK^22,23^ and MAPK^24^ pathways has also been shown to be associated with OCSC properties, and targeting these signaling pathways appears to be a suitable option. Unfortunately, clinical trials testing the anti-OCSC activity of different pathway inhibitors either as monotherapies or in combination with other anticancer drugs have not shown satisfactory efficacy thus far. One of the main reasons for the unpromising results is the intolerable toxicity of the treatments.

In this work, the deubiquitinase OTUD1 is shown to be important for ovarian cell stemness maintenance. High levels of OTUD1 are associated with poor prognosis in ovarian cancer patients. Our study further shows that OTUD1 clearly forms aggresome-like organelles in the cytoplasm via interactions in its N-terminal intrinsically disordered region; these organelles sequester ASK1 and promote its stability, in turn activating the downstream JNK signaling pathway to sustain OSCS maintenance. These findings reveal that aggresome formation contributes to OSCS and plays an important role in cancer progression and recurrence.

## Results

### Multiomics-based screening revealed that OTUD1 is a biomarker of poor prognosis and is associated with CSC maintenance in ovarian cancer

**Fig. 1:**
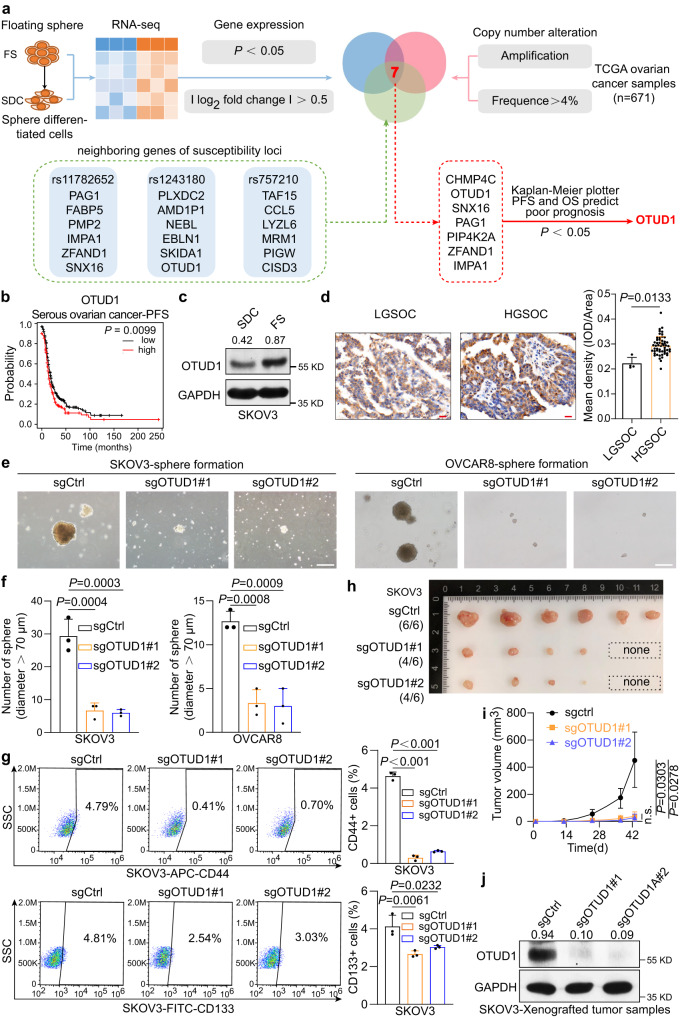
OTUD1 is identified to be associated with OSCS maintenance and its high expression positively correlate with histological high-grade ovarian serous cancers. **a** A multiomic integration approach was developed to screen the key factor controlling self-renewal and maintenance of OCSC. SKOV3 floating spheres and the derived differentiated cells were subjected to RNA-seq analysis, which is sorted by $-{\log }_{10}\,{P\; {{{{\rm{Value}}}}}}$. The obtained differentially expressed genes were further screened based on the gene dataset of susceptibility loci for ovarian cancer and their negatively correlation with tumor prognosis. SDC Sphere Differentiated Cells. FS Floating Sphere. **b** Kaplan–Meier survival curves revealed a negative correlation between OTUD1 expression level and progression-free survival (PFS) in individuals with serous ovarian cancer (https://kmplot.com/analysis/). *n* = 535 patients with serous ovarian cancer. **c** The protein level of OTUD1 were measured in SKOV3 floating spheres and the derived differentiated cells. SDC Sphere Differentiated Cells. FS Floating Sphere. **d** Immunohistochemistry analysis of OTUD1 protein abundance in ovarian serous cancer tissue microarray; representative images of indicated pathological grade are shown (Scale bar, 20 μm). LGSOC samples, *n* = 4; HGSOC samples, *n* = 54. HGSOC, High grade serous ovarian carcinoma. LGSOC Low grade serous ovarian carcinoma. Representative images (**e**) and statistical analysis (**f**) demonstrating the effects of OTUD1 knockout on floating sphere-forming capacity in SKOV3 and OVCAR8 cell lines (*n* = 3). Scale bar, 50 μm. **g** Flow cytometry analysis of CD44^+^ and CD133^+^ CSCs in SKOV3 OTUD1 depleted cell line. Numbers in the charts indicate the percentages of corresponding subpopulations (*n* = 3). Tumor images (**h**) and volumes (**i**) in mice injected with negative control and OTUD1 knockout SKOV3 cells (*n* = 6 mice per group). Tumor sizes were monitored and the protein level of xenografts were analyzed by western blot (**j**). *P* values are calculated using two-tailed unpaired Student’s *t* test (**d**), one-way ANOVA (**f**, **g**) and two-way ANOVA (**i**). n.s. not significant. Representative of *n* = 3 independent experiments (**c**, **e**, **g**). Source data are provided as a Source Data file.

We conducted this study with the aim of systematically identifying the biomarkers driving tumorigenesis and maintaining OSCS in human ovarian cancer. To achieve that goal, we analyzed genes that show altered gene expression at the transcriptional level during the transition from OCSCs to differentiated cells, factors that predict poor prognosis, and genes that are involved in the amplified genomic regions and susceptibility loci. Therefore, an integrated multiomics approach based on the above aspects was developed to screen for the key factors controlling self-renewal and stemness maintenance (Fig. 1a). SKOV3 cells were seeded in ultralow-attachment 96-well plates in DMEM/F12 medium supplemented with 2% B27 serum replacement to allow the formation of floating spheres. Then, these spheres were collected and recultured in a 6-well plate in DMEM with 10% FBS to allow the cells in the spheres to differentiate and attach to the bottom surface, according to a previous study^25^. The cell states were determined by using qPCR to confirm the high expression of stemness-associated genes (i.e., *NOTCH1*, *NANOG*, *OCT4*, *SOX2*, *CD44)* in the floating spheres but not in the differentiated cells (Supplementary Fig. 1a). Subsequently, tumor floating spheres and the derived differentiated cells were subjected to RNA-seq analysis, and differentially expressed genes (*P* < 0.05 and $\left|{\log }_{2}{fold\; change}\right|$ > 0.5) were identified (Fig. 1a). An early study identified rs11782652, rs1243180, and rs757210 as susceptibility loci for ovarian cancer^26^. We assumed that neighboring genes of those loci might be associated with OCSC traits; thus, all of the genes within 200 kb of those loci were chosen (Supplementary Data 1). Moreover, based on human sample data in The Cancer Genome Atlas (TCGA), we identified genes with copy number amplification (frequency >4%) (Supplementary Data 2). By analyzing the intersection of the three gene sets described above, we obtained seven candidate genes (CHMP4C, OTUD1, SNX16, PAG1, PIP4K2A, ZFAND1, IMPA1). Among these genes, only OTUD1, a deubiquitinase, was selected because its high level strongly predicts poor prognosis in serous ovarian cancer (Fig. 1b, Supplementary Fig. 1b) as well as in several other cancer types (Supplementary Fig. 1c). The OTUD1 protein level decreased as the tumor spheres derived from SKOV3 or OVCAR8 cells underwent differentiation (Fig. 1c and Supplementary Fig. 1d and 1e); coincidentally, samples of high-grade serous ovarian cancer (HGSOC), which is a poorly differentiated subtype and possesses a higher mRNA expression-based stemness index (mRNAsi) (Supplementary Fig. 1f), contained higher levels of OTUD1 than samples of low-grade serous ovarian cancer subtypes (Fig. 1d). Taken together, these results strongly suggest that OTUD1 may act as a proto-oncogene in ovarian cancer and potentiate OSCS maintenance.

### OTUD1 knockout effectively decreased tumorigenicity and OSCS but not the proliferation rate of ovarian cancer cells

To verify our hypothesis, we knocked out OTUD1 in the ovarian cancer cell lines SKOV3, OVCAR8 and CAOV3 by using two distinct sgRNAs (Supplementary Fig. 1g). Relative to control cells, cells with OTUD1 knockout (KO) resulted in compromised tumor sphere formation (Fig. 1e, f, and Supplementary Fig. 1h), soft agar colony formation (Supplementary Fig. 1i) and tumor cell invasion ability (Supplementary Fig. 1j). In addition, a flow cytometric cell sorting assay showed that loss of OTUD1 obviously decreased the cancer stem cell ratio (Fig. 1g). Finally, a mouse xenograft tumor model was used to determine the effects of OTUD1 KO on tumorigenesis in vivo. The results showed that compared with controls, tumors derived from knockout cells grew more slowly and were much smaller (Fig. 1h–j, and Supplementary Fig. 1k-l). Interestingly, depletion of OTUD1 did not affect SKOV3 cell growth (Supplementary Fig. 1m), implying that OTUD1 probably contributes to cancer progression through its effect on OSCS.

### OTUD1 is involved in MAPK/JNK pathway regulation and physically interacts with ASK1

**Fig. 2:**
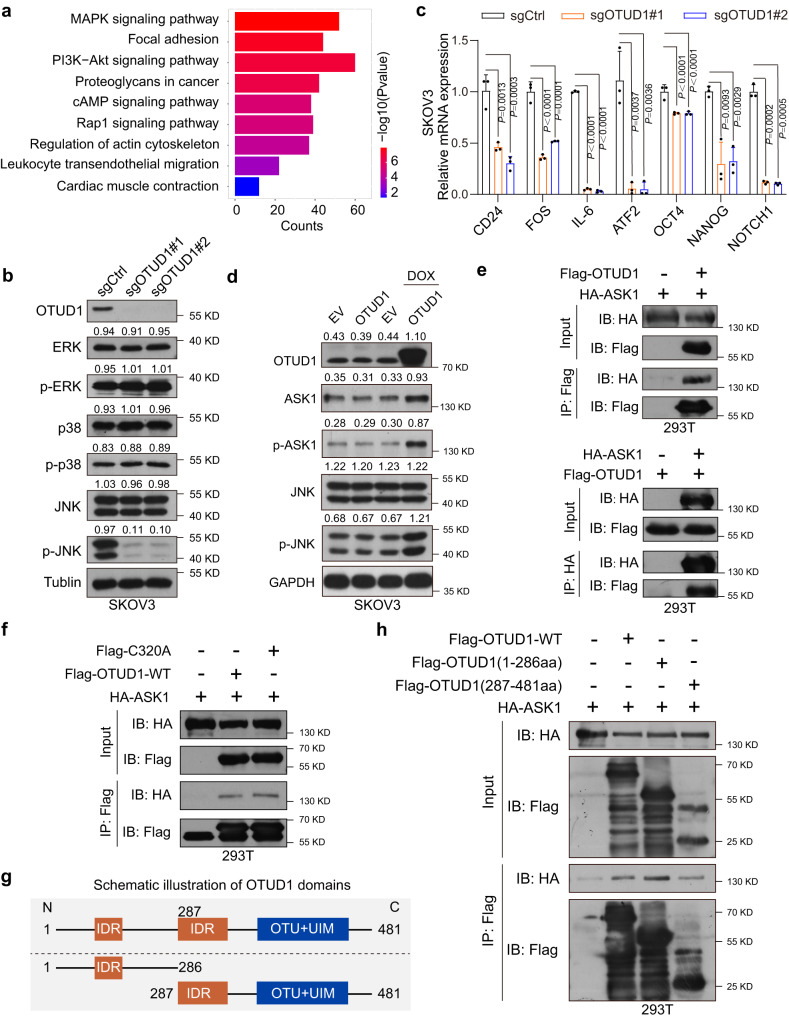
OTUD1 plays a role in MAPK/JNK pathway regulation and physically interacts with ASK1. **a** Kyoto Encyclopedia of Genes and Genomes (KEGG) analysis of the differentially expressed genes between sgRNA-OTUD1 and control cells (sgCtrl). The top 9 enriched pathways were listed. The bubble size indicates changed gene numbers and colors represent false discovery rate (*P*‐value). **b** The specific effect of OTUD1 on the phosphorylation levels of several key components of the MAPK signaling pathway (ERK/p-ERK, p38/p-p38, JNK/p-JNK) were determined. **c** The expression of *CD24*, *FOS* and several other reported JNK target genes (*IL6* and *ATF2*), as well as a number of CSCs markers such as *OCT4, NANOG* and *NOTCH1* in SKOV3 control or OTUD1 depleted cells, were analyzed by qPCR (*n* = 3). **d** A dox-inducible EGFP-OTUD1 expression system was established to evaluate the effects of OTUD1 on protein levels of JNK signal pathway key components (ASK1/p-ASK1, JNK/p-JNK). Dox doxorubicin. **e** Co-immunoprecipitation (Co-IP) was performed with Flag-OTUD1 WT and HA-ASK1 in 293T cells. **f** Co-immunoprecipitation (Co-IP) was performed with Flag-OTUD1 WT or Flag-OTUD1 C320A mutant and HA-ASK1 in 293T cells. C320A, OTUD1-C320A. **g** Schematic illustration of OTUD1 domain. OTU ovarian tumor protease, UIM ubiquitin-interacting motif, IDR intrinsically disordered protein region. **h** Co-immunoprecipitation (Co-IP) was performed with Flag-OTUD1 (aa 1–286 or aa 287–481 truncation) and HA-ASK1 in 293T cells. *P* values are calculated using one-way ANOVA (**c**). Representative of *n* = 3 independent experiments (**b**, **d**–**f**, **h**). Source data are provided as a Source Data file.

To determine the mechanism by which OTUD1 promotes OSCS maintenance, we performed RNA-seq using SKOV3 cells engineered to express sgNC or sgOTUD1. Based on the Kyoto Encyclopedia of Genes and Genomes (KEGG) pathway enrichment analysis of the identified differentially expressed genes, several pathways, including the MAPK, AKT, Rap1, and focal adhesion pathways, were enriched (Fig. 2a). Given that the KEGG analysis showed MAPK pathway enrichment and that many previous studies reported the importance of MAPK/JNK activation in ovarian CSC activity regulation^27–29^, although it may play different roles in different cancer types^30–33^, we focused on the effect of OTUD1 on the MAPK/JNK pathway. p-ERK, p-p38 and p-JNK levels in OTUD1 knockout cells were determined. The results showed that only p-JNK was significantly downregulated (Fig. 2b). Indeed, OTUD1 KO downregulated the expression of several reported JNK target genes and CSC-associated genes, which was confirmed by qPCR (Fig. 2c, and Supplementary Fig. 2a, b). Because OTUD1 can typically stabilize proteins via its deubiquitinase activity, to identify the possible proteins in the JNK pathway that are targeted by OTUD1, we first detected the changes in the protein levels of several main JNK components in the presence of OTUD1. An EGFP-OTUD1 inducible system was then constructed to evaluate the effect of OTUD1 on JNK pathway proteins. As shown in Fig. 2d, ectopic OTUD1 expression upregulated apoptosis signal-regulation kinase-1 (ASK1) and increased ASK1 phosphorylation levels. ASK1 is a mitogen-activated protein kinase family member (MAP3K5) that activates JNK pathways in response to intracellular and extracellular stimuli^34^, and high ASK1 mRNA expression predicts poor prognosis in ovarian cancer (Supplementary Fig. 2c). Moreover, JNK phosphorylation was also significantly increased (Fig. 2d). To firmly establish a link between JNK and the maintenance of stemness, we treated OTUD1-expressing cells with T-5224 (T-5), which is a selective c-Jun inhibitor. Consistently, T-5224 treatment significantly compromised the promoting effect of ectopic OTUD1 on the expression of key CSC genes (Supplementary Fig. 2d). In addition, loss of OTUD1 reduced the c-Jun phosphorylation level in SKOV3, OVCAR8 and CAOV3 cells (Supplementary Fig. 2e), implying that OTUD1 probably regulates OSCS by activating the JNK/c-Jun pathway.

Moreover, depletion of OTUD1 reduced the ASK1 phosphorylation level in SKOV3 and OVCAR8 cells (Supplementary Fig. 2f). We then examined the interaction between OTUD1 and ASK1. Reciprocal coimmunoprecipitation (Co-IP) revealed that OTUD1 binds to ASK1 (Fig. 2e), and their interaction was not influenced by the enzymatically inactive OTUD1 C320A mutant (Fig. 2f). To characterize the domains involved in the protein interaction, a series of truncated constructs were generated. As shown in Fig. 2g, h, both the N-terminus and C-terminus of OTUD1 bound to ASK1. These results prompted us to investigate whether OTUD1 is sufficient to activate ASK1 downstream signaling and whether its deubiquitinase activity is needed for this regulation.

### OTUD1 promotes ASK1 stability in a largely deubiquitinase-independent manner

**Fig. 3:**
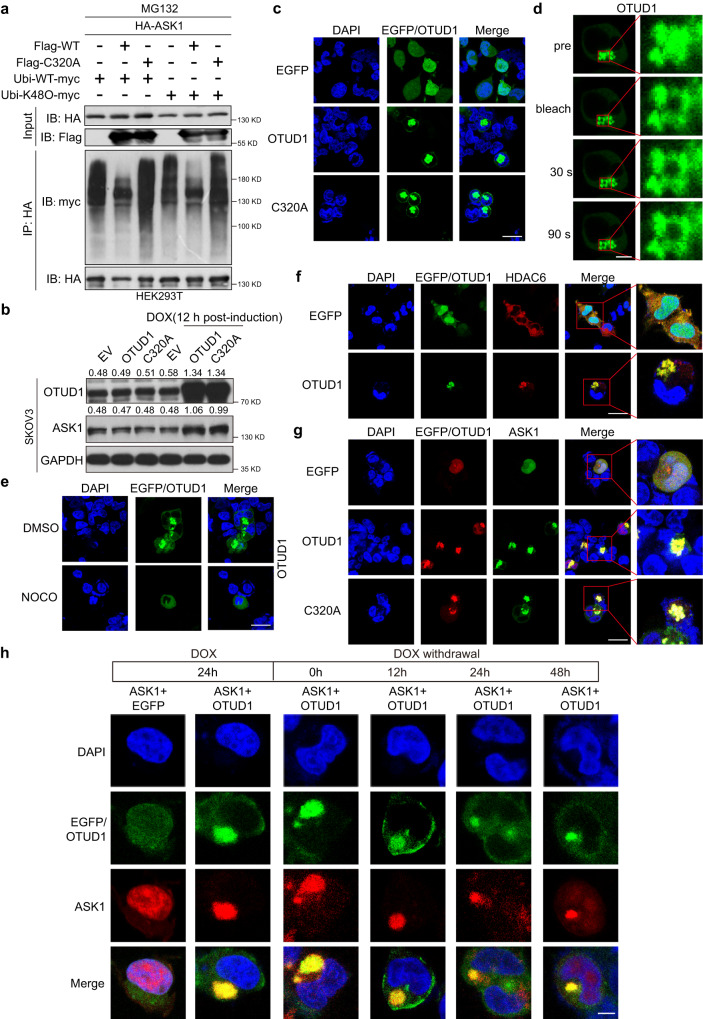
Elevated OTUD1 protein forms aggresome and re-localize ASK1 in the cytoplasm. **a** IB analysis of input and IP products from 293T cells transfected with Flag-OTUD1 (Flag-WT) or Flag-C320A, ubiquitin-myc (Ubi-myc) or Ubi-K48O-myc, and HA-ASK1. The cell lysates were treated with protease inhibitor MG132 prior to Co-IP experiment. WT, OTUD1-WT; C320A, OTUD1-C320A. **b** Two dox-inducible EGFP-OTUD1-WT and EGFP-OTUD1-C320A expression system were established to assess the effect of OTUD1 on ASK1 protein levels. Dox, doxorubicin. **c** Representative immunofluorescence images of 293T cells transiently transfected with EGFP and EGFP-OTUD1 WT or EGFP-C320A plasmids. Scale bar, 25 μm. **d** Representative images of fluorescence recovery at prebleach (0 s) and postbleach (30 s, and 90 s). Scale bar, 10 μm. **e** Representative immunofluorescence images of indicated 293T cells treated with NOCO. Scale bar, 25 μm. NOCO, nocodazole. **f** Immunofluorescence was performed to observe the co-localization of EGFP-OTUD1 and HDAC6 (aggresome biomarker). Scale bar, 25 μm. **g** Representative immunofluorescence images of 293T cells co-transfected with mcherry-ASK1 and EGFP-OTUD1 or EGFP-C320A plasmid. Scale bar, 25 μm. **h** A dox-inducible 293T EGFP-OTUD1 expression system was generated. The resultant cells were transfected with mcherry-ASK1 and treated with doxorubicin (DOX) (1 μM) for 24 h before the media was changed. Subsequently, the aggregation of OTUD1 and ASK1 was observed by Laser Scanning Confocal Microscope at 0, 12 h, 24 h, and 48 h after DOX (1 μM) was removed. Scale bar, 5 μm. Dox doxorubicin. Representative of *n* = 3 independent experiments (**a**–**h**). Source data are provided as a Source Data file.

As a deubiquitinase, wild-type (WT) OTUD1 but not the C320A mutant efficiently reduced ASK1 K48 ubiquitination (Fig. 3a), implying that OTUD1 inhibits ASK1 degradation in a proteasome-dependent manner. The EGFP-OTUD1-WT (WT) and EGFP-OTUD1-C320A (C320A) inducible plasmids were then constructed to evaluate the effect of OTUD1 on ASK1 levels. As shown in Fig. 3b, ectopic expression of WT OTUD1 increased the ASK1 level, consistent with the observation that OTUD1 reduced ASK1 K48 ubiquitination. Surprisingly, expression of the OTUD1 deubiquitinase-dead C320A mutant increased the ASK1 protein level to an extent comparable to that resulting from WT OTUD1 expression (Fig. 3b). We therefore speculated that OTUD1 might stabilize ASK1 in an essentially deubiquitinase-independent manner.

### Elevated OTUD1 protein levels induce the formation of aggresome-like organelles and relocalizes ASK1

We first investigated the cellular location of OTUD1 by using an EGFP-tagged construct. Unexpectedly, when overexpressing EGFP-OTUD1-WT or C320A, we observed an area of protein aggregation with an irregular border in the cytoplasm in most of the cells with EGFP signals (the percentage of the aggregates was 0% for the control, 75.0% for WT and 66.4% for C320A; the mean diameter of the aggregates was 0 μm for the control, 9.2 μm for WT and 8.2 μm for C320A (Fig. 3c, and Supplementary Fig. 3a). To characterize the properties of the aggregation, we performed a fluorescence recovery after photobleaching (FRAP) assay. As shown in Fig. 3d and Supplementary Fig. 3b, live 293T cells transiently transfected with EGFP-OTUD1 WT were subjected to laser microirradiation. FRAP was performed up to 90 s after microirradiation, and no EGFP signal recovery was observed at the irradiation site, suggesting that the aggregation was unlikely to be a result of phase separation. Consistent with these findings, when cells were treated with 1,6-hexanediol (1,6-HDO), a phase separation inhibitor, aggregation was not affected (Supplementary Fig. 3c). We then hypothesized that if the aggregation was an aggresome, which is normally formed depending on the presence of microtubules^35^. To verify this hypothesis, we treated cells with nocodazole (NOCO), a microtubule inhibitor, to disrupt microtubules and block the formation of aggresomes. The aggregation dramatically disappeared in response to nocodazole treatment, indicating that the ectopic OTUD1-mediated aggregates were probably aggresomes (Fig. 3e). To further prove this hypothesis, we investigated the colocalization of OTUD1 and several aggresome markers, namely, HDAC6, P62 and HSP70, and the results showed that OTUD1-based aggresomes were colocalized with all of these markers very prominently (Fig. 3f, Supplementary Fig. 3d–e), reinforcing the idea that OTUD1 can form aggresomes without additional proteasome inhibition. Because OTUD1 strongly bound to ASK1, we sought to determine whether ASK1 could be recruited to OTUD1-based aggresomes. As shown in Fig. 3g, compared with the observations in control cells, ASK1 aggregated upon OTUD1 overexpression, and both WT OTUD1 and the C320A mutant completely colocalized with ASK1. An EGFP-OTUD1 inducible plasmid was then constructed to evaluate the dynamics of the colocalization of OTUD1 and ASK1. As shown in Fig. 3h and Supplementary Fig. 3f, after treating 293T cells with doxorubicin (DOX) for 24 h, OTUD1-based aggresomes were observed, and they were colocalized with ASK1. Interestingly, the aggresomes gradually disappeared upon DOX removal, accompanied by a large decrease in the size of the ASK1 condensate. Moreover, we treated cells expressing OTUD1 with DMSO or NOCO. As shown in Supplementary Fig. 3g, NOCO treatment gradually eliminated OTUD1 aggresome formation, accompanied by a reduction in ASK1 puncta, suggesting that ASK1 aggregation is completely dependent on OTUD1-based aggresome formation. Because aggresomes are partially resistant to proteasomal degradation, we assumed that ASK1 sequestration by OTUD1-based aggresomes significantly promotes ASK1 protein stability and, in turn, activates the downstream JNK pathway.

### The N-terminal intrinsically disordered region of OTUD1 is critical for its supramolecular assembly, aggresome formation, and associated ASK1 recruitment

**Fig. 4:**
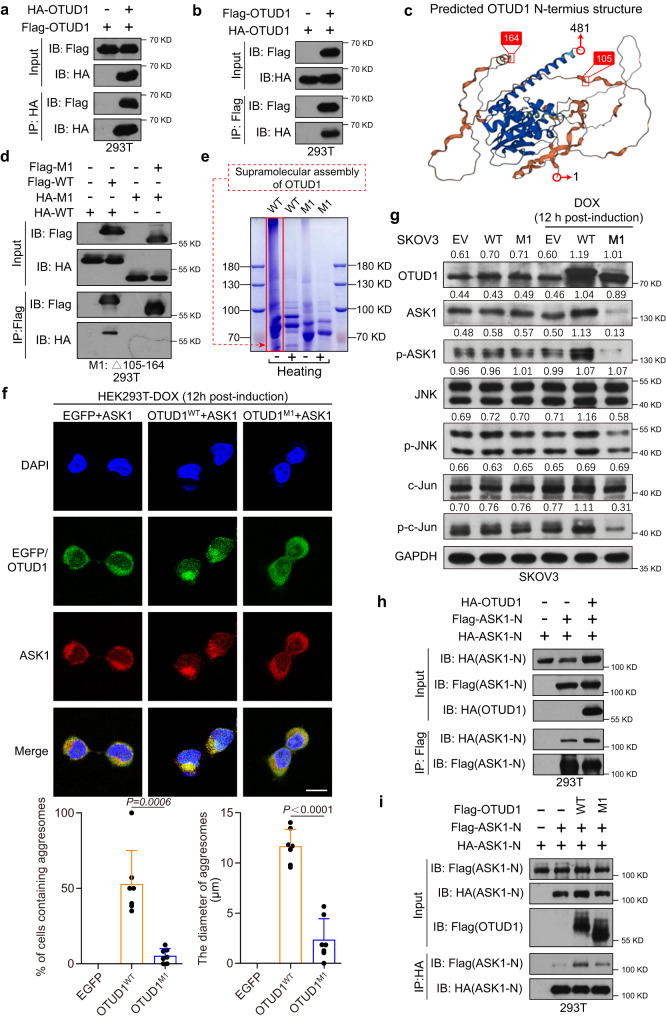
A peptide locates in OTUD1 N-terminal intrinsic disordered region is critical for aggresomes formation and ASK1 recruitment. **a**, **b** IB analysis of input and IP products from 293T cells transfected with Flag- and HA-tagged OTUD1. **c** The predicted three-dimensional protein structure of OTUD1 was obtained from AlphaFold Protein Structure Database (https://alphafold.ebi.ac.uk/entry/Q5VV17). **d** IB analysis of input and IP products from 293T cells transfected with full-length (OTUD1-WT) or OTUD1 IDR mutant plasmid (Δ105–164, M1). IDR, intrinsically disordered protein region. WT, OTUD1-WT; M1, OTUD1-Δ105–164. **e** Gel-shift experiments showing the super-assembly of the purified OTUD1 protein (WT) and Δ105–164 deletion mutant protein (M1). **f** Dox-inducible EGFP-OTUD1 (EGFP-OTUD1^WT^) and EGFP-OTUD1-Δ105–164 (EGFP-OTUD1^M1^) expression plasmids were constructed and transfected into 293T. Representative images of Immunofluorescence on 293T cells co-transfected with mcherry-ASK1 and EGFP-OTUD1^WT^ or EGFP-OTUD1^M1^ plasmid. Scale bar, 10 μm. Statistical analysis showing the percentage of cells containing observable aggresome and their average diameter of OTUD1 (WT) or mutant OTUD1 proteins (Δ105–164, M1). *n* = 7 EGFP positive cells examined across 3 independent experiments. Dox, doxorubicin. **g** The dox-inducible EGFP-OTUD1 (EGFP-OTUD1^WT^) and EGFP-OTUD1-Δ105–164 (EGFP-OTUD1^M1^) expression system was constructed. IB analysis showing the indicated proteins (ASK1, p-ASK1, JNK, p-JNK, c-Jun and p-c-Jun) in SKOV3 cells upon OTUD1^WT^ (WT) or OTUD1^Δ105-164^ (M1) expression. Dox doxorubicin, EV empty vector. **h** IB analysis of input and IP products from 293T cells co-transfected with N-terminus of ASK1 plasmid (Flag- or HA-tagged ASK1) and Flag-OTUD1 to detect the ASK1 dimerization. N, N-terminus. **i** IB analysis of input and IP products from 293T cells transfected with N-terminus of ASK1 plasmid (Flag- or HA-tagged ASK1), Flag-OTUD1^WT^ (WT), and Flag-OTUD1^Δ105-164^ (M1). N, N-terminus. *P* values are calculated using two-tailed unpaired Student’s *t* test (**g**). Representative of *n* = 3 independent experiments (**a**, **b**, **d**–**i**). Source data are provided as a Source Data file.

Previous studies showed that proteasome inhibition can facilitate aggresome formation by several proteins; however, our results provide evidence that an elevated OTUD1 protein level alone was sufficient to induce aggresome formation, implying that OTUD1 might undergo a self-supramolecular assembly process. We first examined the possible oligomerization of the full-length, N-terminus, and C-terminus of the OTUD1 protein. The results clearly showed that both full-length OTUD1 and the N-terminus but not the C-terminus could form complexes because Flag- and HA-tagged OTUD1 interacted with each other (Fig. 4a, b and Supplementary Fig. 4a). Given that aggresome formation is primarily caused by the presence of misfolded proteins, we sought to determine whether OTUD1 contains a unique disordered region that contributes to both its supramolecular assembly and protein aggregation. Indeed, OTUD1 contains an N-terminal intrinsically disordered region (IDR), which was also predicted by the AlphaFold Protein Structure Database (Fig. 4c). After several rounds of screening, an OTUD1 N-terminal peptide located between amino acids (aa) 105 and 164 was shown to be responsible for OTUD1 complex formation (Fig. 4d and Supplementary Fig. 4b–e). Furthermore, while supramolecular assembly of the purified native WT OTUD1 protein was observed in vitro, deletion of aa 105–164 (M1) almost completely abolished OTUD1 aggregation (Fig. 4e).

An EGFP-OTUD1 inducible system was again used to evaluate the dynamics of the colocalization of OTUD1 and ASK1. As shown in Fig. 4f, after treating 293T cells with doxorubicin (DOX) for 12 h, deletion of aa 105–164 disrupted the tendency of OTUD1 to form aggresomes colocalized with ASK1. Accordingly, the expression of Δ105–164 failed to stabilize the ASK1 protein as OTUD1^WT^ did, reinforcing the idea that the recruitment of a protein to aggresomes may enhance its resistance to proteasomal degradation. We then further explored whether the colocalization of ASK1 and OTUD1 affects ASK1 and other JNK protein activities. As shown in Fig. 4g, after treating SKOV3 cells with doxorubicin (DOX) for 12 h, ectopic expression of OTUD1 but not Δ105–164 (M1) increased the phosphorylation levels of ASK1 at Thr838 and the expression levels of the downstream phosphorylated forms of JNK and c-Jun, all of which indicate ASK1 activation. Moreover, we observed enhanced dimerization of ASK1, which is essential for ASK1 activation, most likely because of enforced proximity (Fig. 4h). Consistent with these findings, depletion of aa 105–164 in OTUD1 attenuated ASK1 dimerization (Fig. 4i), suggesting that ASK1 recruitment mediated by aggresomes not only increased its protein stability but also maintained its activity. In addition, we performed an immunofluorescence experiment to detect whether proteins sequestered in the aggresome could be functional or continuously activated. As shown in Supplementary Fig. 4f-4g, enhanced p-ASK1 and p-JNK signal intensities were observed. Moreover, we found that both p-ASK1 and p-JNK were enriched in OTUD1 WT-based aggresomes, while deletion of OTUD1 105–164 aa (M1) failed to sequester p-ASK1 and p-JNK, suggesting that those proteins sequestered in the aggresome could be functional or continuously activated. All the above results demonstrated that OTUD1-based aggresomes contribute to ASK1 protein stabilization and persistent ASK1/JNK activation and that this function is mediated by a region (aa 105–164) located in the OTUD1 N-terminal intrinsically disordered region.

### Abrogation of OTUD1 aggresome formation impaired the stimulatory effect of OTUD1 on OSCS maintenance

**Fig. 5:**
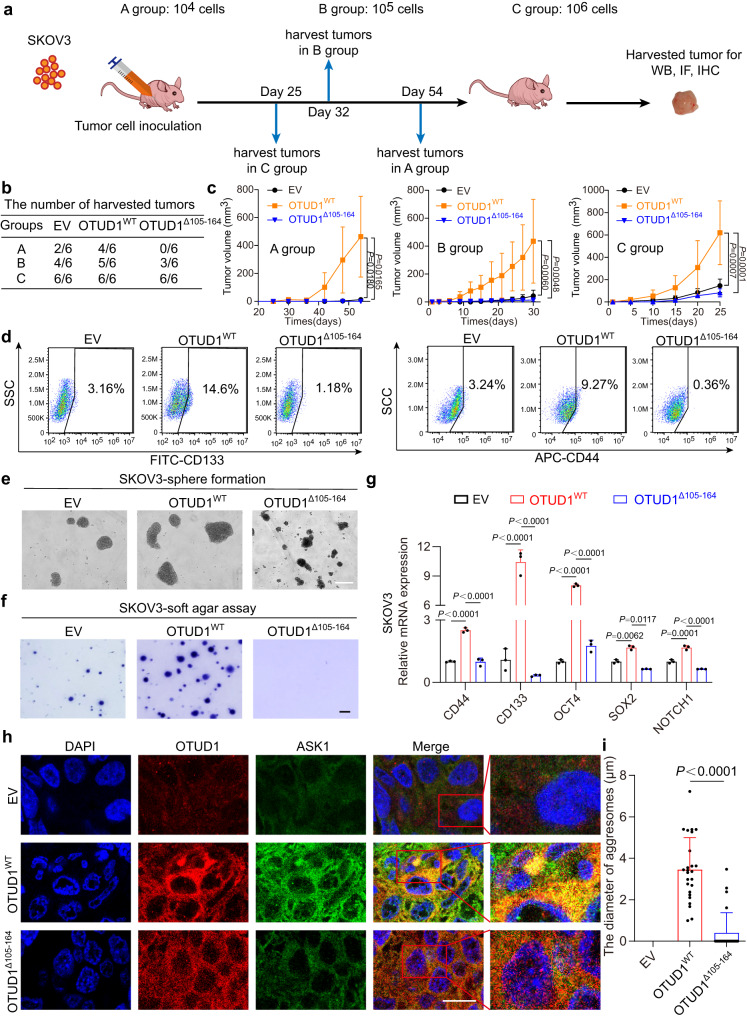
Aggresome formation of OTUD1 is critical for promoting OSCS maintenance. 1 × 10^4^, 1 × 10^5^ or 1 × 10^6^ SKOV3 cells were subcutaneously injected into BALB/c nude female mice (*n* = 6 mice per group). After 25, 32, or 54 days, the mice were sacrificed and the tumor tissues were collected as shown in **a**. The table shows the number of harvested tumors in each group (**b**). Tumor growth curve of indicated xenografts are created based on tumor volume of indicated time (**c**). EV empty vector. **d** Effect of WT OTUD1 or Δ105–164 mutant on CD44^+^ or CD133^+^ cells proportions were evaluated by flow cytometry analysis. EV empty vector. **e** Representative images of the effects of depletion of OTUD1 IDR region (105–164 aa) on sphere-formation in SKOV3 cells. Scale bar, 50 μm. IDR intrinsically disordered protein region. EV empty vector. **f** Representative images of the effect of WT (OTUD1^WT^) or IDR depleted mutant OTUD1 (OTUD1^Δ105–164^) on anchor-independent growth capacity in SKOV3 cells. Scale bar, 400 μm. EV empty vector. **g** qPCR was employed to confirm the expression of stemness-associated genes (ie, *CD133, CD44, CD24, OCT4, SOX2, NOTCH1*) in SKOV3 OTUD1^WT^ or OTUD1^Δ105-164^ expressing cells (*n* = 3). EV empty vector. **h** Immunofluorescence analysis was performed to examine the correlation of OTUD1^WT^ or OTUD1^Δ105–164^ expression and ASK1 protein level or distribution in vivo. Scale bar, 15 μm. **i** Statistical analysis showing the OTUD1-based aggresome average diameter of OTUD1 (WT) or mutant OTUD1 proteins (Δ105–164, M1) in SKOV3 xenografted tumor samples (*n* = 25 cells examined over 2 xenografted tumor samples). EV, empty vector. *P* values are calculated using two-way ANOVA (**c**), one-way ANOVA (**g**), two-tailed unpaired Student’s *t* test (**h**). Representative of *n* = 3 independent experiments (**d**–**f**). Source data are provided as a Source Data file.

Because the active JNK pathway has been shown to be involved in CSC maintenance by numerous studies^22,36^, we focused on the stimulatory effect of both OTUD1^WT^ and OTUD1^Δ105-164^ on OCSCs. To achieve this goal, we initially performed a xenotransplantation limiting dilution assay to investigate the effects of OTUD1^WT^ or mutant on the OSCS. A total of 1 × 10^4^, 1 × 10^5^, or 1 × 10^6^ SKOV3 cells (EV, OTUD1^WT^, OTUD1^∆105-164^) were counted and subcutaneously injected into BALB/c nude female mice. The results showed that OTUD1^WT^-expressing cells exhibited much higher tumor initiation rates and greater tumor growth potential than control cells. In contrast, the deletion of aa 105–164 markedly impaired the induced tumorigenicity, which was reflected by reductions in both the average tumor volume and number (Fig. 5a–c). Next, the CD133^+^ and CD44^+^ cell proportions in each group were determined. The results showed that ectopic expression of OTUD1^WT^ but not OTUD1^∆105-164^ in SKOV3 cells greatly increased the CD133^+^ and CD44^+^ cell proportions (Fig. 5d and Supplementary Fig. 5a). Similarly, expression of OTUD1^WT^ but not OTUD1^∆105-164^ in SKOV3 cells promoted sphere formation and anchorage-independent growth (soft agar assay) (Fig. 5e, f and Supplementary Fig. 5b–d). Accordingly, the expression of OTUD1^WT^ but not the ∆105–164 mutant upregulated the expression of several OCSC marker genes (Fig. 5g). Rescue experiments by re-expressing WT- or M1-OTUD1 in SKOV3 OTUD1-depleted cells were performed to exclude possible off-target effects. As shown in Supplementary Fig. 5e–g, WT-OTUD1 but not the M1 mutant re-expression increased sphere formation and colony formation. These results suggested that OTUD1-based aggresomes are critical for maintaining OSCS. In addition, OTUD1 and ASK1 subcellular localization was analyzed in the xenograft tumor tissues. The results showed that OTUD1^WT^ can aggregate and essentially colocalize with ASK1, while Δ105–164 caused almost no protein aggregation (Fig. 5h, i). IHC analysis of xenograft tumors also revealed that OTUD1^WT^ but not OTUD1^∆105–164^ increased ASK1 protein levels and JNK phosphorylation (Supplementary Fig. 5h). These results strongly suggest that the ASK1/JNK pathway is a therapeutic target in OTUD1-high OCSCs.

### ASK1/JNK pathway inhibitors effectively suppressed OTUD1-mediated OSCS

Targeting the JNK signaling pathway has been recognized as a strategy in cancer therapy, and several JNK inhibitors have been developed. However, the potency of these inhibitors is limited due to the lack of biomarkers. IN-8 is a specific inhibitor of the Jun N-terminal kinase. Selonsertib (SE) is a selective ASK1 inhibitor that was tested for NASH treatment in a clinical trial^37^. Moreover, a recent study showed that the burton tyrosine kinase inhibitor ibrutinib, which has been approved by the FDA for the treatment of lymphoma and chronic lymphocytic leukemia, is an MKK7 inhibitor that can inhibit the ASK1/JNK pathway^38^. We therefore tested the effect of these inhibitors on OSCS driven by OTUD1.

**Fig. 6:**
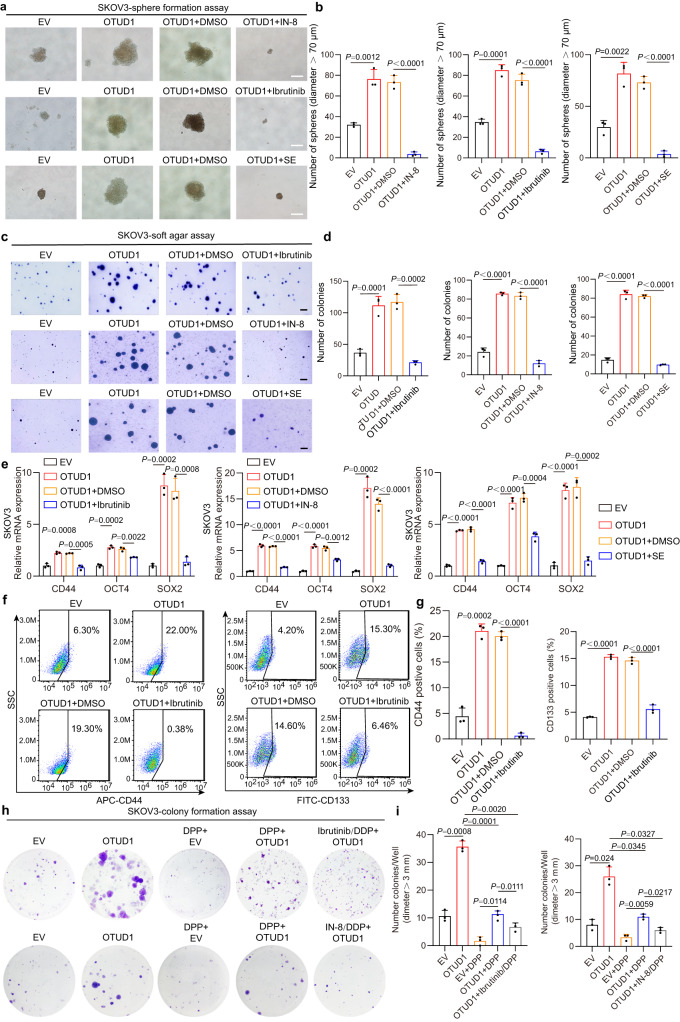
ASK1/JNK inhibitors potentially suppressed stemness of OTUD1^high^ ovarian cancer cells. **a** Representative images demonstrate the effects of IN-8 (2 μg/mL), selonsertib (SE, 1 μM), or ibrutinib (2.5 μg/mL) treatment on SKOV3 sphere-formation with or without ectopic OTUD1, respectively. EV empty vector. Scale bar, 50 μm. **b** Statistical analysis reveals the effect of inhibitors (IN8, Selonsertib, and Ibrutinib) on number of floating spheres of indicated SKOV3 cells in Fig. 6a (*n* = 3). EV empty vector, SE selonsertib. **c** The soft agar assay showing anchorage-independent growth of OTUD1 cells in SKOV3 treated with or without IN-8 (2 μg/mL), Selonsertib (SE, 1 μM), or Ibrutinib (2.5 μg/mL). Scale bar, 400 μm. **d** Statistical analysis illustrates the effect of inhibitors (IN8, Selonsertib, and Ibrutinib) on number of colonies in indicated SKOV3 cells in Fig. 6c (*n* = 3). EV empty vector, SE selonsertib. **e** The qPCR assay was utilized to confirm the expression of stemness-associated genes (i.e., *CD44, OCT4*, and *SOX2*) in SKOV3 cells expressing OTUD1 with indicated treatment (*n* = 3). EV empty vector, SE selonsertib. **f** Flow cytometry analysis of CD44^+^ and CD133^+^ CSCs in SKOV3 OTUD1^WT^ cell with indicated treatment. Numbers in the charts indicate the percentages of corresponding subpopulations. EV empty vector. **g** Statistical analysis showing the effect of those three inhibitors (IN8, Selonsertib, and Ibrutinib) on the percentages of CSCs in Fig. 6f (*n* = 3). EV empty vector. **h** The chemotherapy sensitivity assay by using SKOV3 cells expressing OTUD1 with or without IN-8 (2 μg/mL), Ibrutinib (2.5 μg/mL), or/and DPP (1 μg/mL) treatment. DPP Cisplatin, EV empty vector. **i** Statistical analysis displays the effect of those three inhibitors (IN8, Selonsertib, and Ibrutinib) on the clonal formation in Fig. 6h (*n* = 3). EV empty vector. *P* values are calculated using unpaired Student’s *t* test (**b**, **d**, **e**, **g**), one-way ANOVA (**i**). Representative of *n* = 3 independent experiments (**a**, **c**, **f**, **h**). Source data are provided as a Source Data file.

As shown in Fig. 6a–d, Supplementary Figs. 6a, b and 7a, b, OTUD1 promoted sphere formation and anchorage-independent growth in soft agar, while the promoting effect was significantly compromised by administration of IN-8, SP600125, selonsertin or ibrutinib, as determined by the downregulated expression of key CSC genes and reduced CSC content (Fig. 6e–g, Supplementary Figs. 6c and 7c), indicating that targeting the ASKI/JNK pathway is a potential therapeutic strategy for OTUD1^high^ ovarian cancer. In addition, platinum-based chemotherapy efficacy is determined by the presence of CSCs. Indeed, our results showed that OTUD1 expression reduced the sensitivity of SKOV3, OVCAR8 and CAOV3 cells to cisplatin treatment, most likely by promoting OSCS maintenance (Fig. 6h, i, Supplementary Figs. 6d–g and 7d). Interestingly, treatment with IN8, SP600125 or ibrutinib essentially enhanced the sensitivity to platinum-based (cisplatin, DDP) chemotherapy, suggesting that the combination of ASK1/JNK pathway inhibition and platinum-based chemotherapy might represent a therapeutic strategy in ovarian cancer. These results also suggested that persistent ASK1/JNK activation is important for OTUD1^high^ OSCS. Because ibrutinib is an FDA-approved drug with tolerable toxicity, we focused on the effect of ibrutinib on the tumorigenicity of OTUD1-high ovarian cancer cells.

### The FDA-approved drug ibrutinib preferentially reduces the tumorigenicity of OTUD1^high^ ovarian cancer cells

**Fig. 7:**
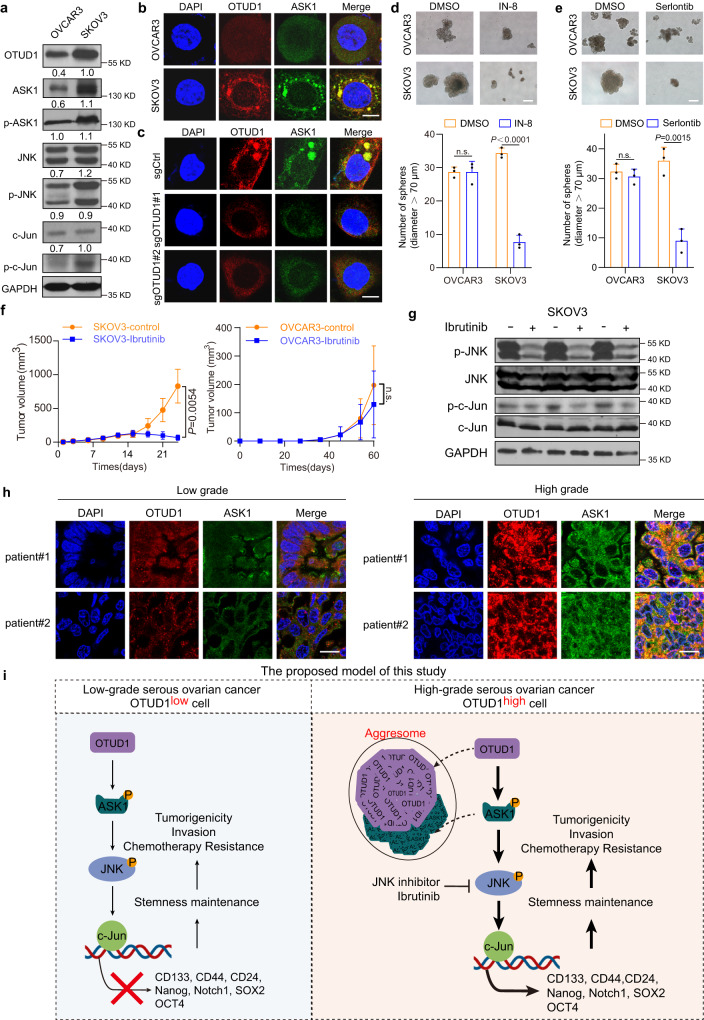
FDA-approved MKK7 inhibitor ibrutinib preferentially inhibit tumorigenicity of OTUD1^high^ ovarian cancer cells. **a** IB analysis of ASK1/JNK pathway proteins derived from OVCAR3 and SKOV3 cells. **b** Representative immunofluorescence images of the aggresome formation in wild-type OVCAR3 and SKOV3 cells. Scale bar, 10 μm. **c** Representative immunofluorescence images of the aggresome formation. Immunofluorescence assay was used to evaluate the effect of OTUD1 depletion on ASK1 aggregation in SKOV3 cells. Scale bar, 10 μm. **d** The sphere formation assay was used to examine the effect of IN-8 (2 μg/mL) on spheres formation of OVCAR3 and SKOV3 cells (above panel). Scale bar, 50 μm. Statistical analysis showing the effect of IN-8 on number of floating spheres (bottom panel). *n* = 3. **e** The sphere formation assay was used to examine the effect of Selonsertib (1 μM) on spheres formation of OVCAR3 and SKOV3 cells (above panel). Scale bar, 50 μm. Statistical analysis showing the effect of Selonsertib on number of floating spheres (bottom panel). *n* = 3. **f** SKOV3 cells or OVCAR3 were subcutaneously injected into BALB/c nude female mice (*n* = 6 mice per group) with or without ibrutinib administration (25 mg/kg). After 24 d or 62 d, the mice were sacrificed, tumors were collected and their volume was measured. Tumor growth curve of indicated xenografts are used to reflect the tumor volume. **g** IB analysis of the JNK pathway proteins (JNK/p-JNK, c-Jun/c-Jun) derived from xenografted tumors with or without ibrutinib treatment. **h** Immunofluorescence assay was used to investigate the correlation of OTUD1 protein level and ASK1 distribution in clinical serous ovarian cancer tissues. Scale bar, 10 μm. **I** A proposed model of this study. *P* values are calculated using unpaired Student’s *t* test (**d**, **e**), two-way ANOVA (**f**). n.s. not significant. Representative of *n* = 3 independent experiments (**a**–**c**, **e**, **g**). Source data are provided as a Source Data file.

To further verify the effect of ibrutinib, we selected several ovarian cancer cell lines, and SKOV3 cells were shown to contain a relatively higher protein abundance of OTUD1 than OVCAR3 cells, accompanied by elevated protein levels of ASK1, p-ASK1, p-JNK and p-c-Jun (Fig. 7a). In addition, higher OTUD1, ASK1 and p-ASK1 levels were also found in OVCAR8 cells than in OVCAR3 cells (Supplementary Fig. 8a). Consistent with the above findings, OTUD1 aggregated and colocalized with ASK1 in SKOV3 cells but not in OVCAR3 cells (Fig. 7b). Moreover, knocking out OTUD1 in SKOV3 cells abrogated ASK1 aggregation (Fig. 7c). Both SP600125, IN-8 and ibrutinib much more efficiently inhibited sphere formation in both SKOV3 and OVCAR8 cells than in OVCAR3 cells (Fig. 7d, e and Supplementary Fig. 8b–f). Notably, ibrutinib dramatically suppressed tumor growth in a xenograft model established with SKOV3 cells and OVCAR8 cells; in contrast, it only modestly affected OVCAR3 cell-derived tumor growth (Fig. 7f and Supplementary Fig. 8g). Immunoblotting (IB) revealed that the JNK/c-Jun pathway was inhibited by ibrutinib (Fig. 7g). These results strongly suggested that a high level of OTUD1 in ovarian cancer could serve as a useful biomarker for ibrutinib-based OCSC-targeted therapy. To further support this idea, we analyzed the OTUD1 and ASK1 protein levels and distribution in high- and low-grade serous ovarian cancer tissues, and the results revealed that OTUD1 preferentially aggregated and colocalized with ASK1 in high-grade tissues (Fig. 7h). Collectively, these results indicated that the presence of aggresomes is likely to be an etiology of and therapeutic target in certain types of malignancy, including serous ovarian cancer, in addition to neurodegenerative disorders such as Parkinson’s disease and dementia with Lewy bodies (DLB)^39^.

## Discussion

By screening for key components in tumorigenesis and CSC maintenance in ovarian cancer, we identified the deubiquitinase OTUD1 as a unique factor and found that its high expression predicts poor prognosis and potentiates ovarian tumor cell stemness maintenance (Fig. 1). OTUD1 physically interacts with ASK1 and is involved in MAPK pathway regulation, which is essential for the maintenance of CSC properties (Fig. 2). Although OTUD1 is capable of stabilizing the ASK1 protein, its deubiquitinase activity appears to be unnecessary for this effect. Interestingly, the OTUD1 protein clearly forms proteasomal degradation-resistant aggresomes in the cytoplasm and recruits ASK1 to these aggresomes via protein interactions, therefore significantly stabilizing ASK1 while maintaining ASK1 activity (Fig. 3). A peptide located in the intrinsically disordered OTUD1 N-terminal region is responsible for OTUD1 aggresome formation and associated ASK1 aggregation, as well as downstream JNK activation (Fig. 4). Consistent with these findings, the abrogation of OTUD1 aggresome formation efficiently inhibited ovarian cancer cell stemness (Fig. 5). ASK1/JNK inhibitors very effectively suppressed OSCS induced by OTUD1 overexpression (Fig. 6), and ibrutinib, an FDA-approved MKK7 inhibitor, preferentially reduced the tumorigenicity of OTUD1^high^ ovarian cancer cells (Fig. 7). Based on the above observations, we proposed the following model. OTUD1 is more likely to be overexpressed in high-grade serous ovarian cancer tissues, and its overexpression is sufficient for aggresome-like organelle formation via its N-terminal intrinsically disordered region. The resultant aggresome sequesters ASK1 and stabilizes it, thereby activating JNK signaling and significantly promoting tumor cell stemness. Disruption of aggresome formation or treatment with ibrutinib might be a promising therapeutic strategy for OTUD1^high^ ovarian cancer by targeting CSCs (Fig. 7h).

Aggresome formation in the cytosol is a general response of mammalian cells when the capacity of the proteasome is exceeded by the production of misfolded proteins^40^. Because the accumulation of misfolded proteins is cytotoxic, aggresomes are thought to be cytoprotective, and the misfolded proteins are stored or eventually degraded by autophagy. The presence of aggresomes has long been thought to be a hallmark of neurodegenerative diseases; one example is the formation of cytoplasmic TDP-43 aggregates in amyotrophic lateral sclerosis (ALS) and a subtype of frontotemporal lobar degeneration (FTLD)^41^. While some proteins have been shown to be more substantially complexed/aggregated in cancer tissues than in normal tissues, a comprehensive understanding of the pathological role of aggresomes in human cancer is still lacking. The presence of aggresomes has been shown to be correlated with poor prognosis in cancer patients^42^. A previous study showed that structurally destabilized p53 mutants could coaggregate with wild-type p53 and its paralogs p63 and p73, thus blocking the transcriptional activity and proapoptotic function of wild-type p53^43,44^. In contrast, our results indicated that instead of inactivating the sequestered ASK1 protein, OTUD1 stabilizes it and maintains or improves its ability to activate the downstream JNK pathway, most likely because proteins incorporated in aggresomes usually escape from proteasome-dependent degradation. This process is precisely controlled, as ASK1 aggregation is tightly coupled with OTUD1 aggresome formation (Fig. 3), suggesting that aggresomes in tumor cells could promote cancer progression via distinct mechanisms, namely, by either eliminating the tumor suppressor activity of sequestered proteins or protecting targeted proteins from being degraded in a proteasome-dependent manner.

Deubiquitinases remove posttranslational ubiquitin (Ub) modifications from proteins and regulate nearly all Ub-dependent processes^45^. Previous cancer research involving the OTUD1 protein has mainly focused on its canonical deubiquitinase activity. For example, OTUD1 can inhibit colonic inflammation by deubiquitinating RIPK1 to suppress NF-κB signaling^46^. OTUD1 can deubiquitinate SMAD7 to enable SMURF2 binding and subsequent TβRI turnover on the cell surface to increase breast cancer metastasis^47^. OTUD1 was also shown to exacerbate colon cancer progression by deubiquitinating and stabilizing iron-responsive element-binding protein 2 (IREB2)^48^, indicating that OTUD1 could act as an oncogene. On the other hand, a recent study showed that OTUD1 could noncanonically regulate AKT activity through its disordered N-terminal region^49^, a mechanism that has been much less appreciated, probably because OTUD1 lacks homology with any known secondary structure domains, indicating that the exact function of the intrinsically disordered N-terminus of OTUD1 is unknown and remains to be further addressed. In this study, our results clearly showed that high OTUD1 expression is closely correlated with poor prognosis and that a region spanning amino acids 105–164 is capable of mediating OTUD1 self-assembly both in vitro and in vivo, an event that is needed for OTUD1-associated aggresome formation, ASK1 stabilization, tumor cell stemness maintenance and tumorigenesis, underscoring the importance of the disordered OTUD1 N-terminal region in the regulation of characteristic cancer signaling pathways and reinforcing the idea that OTUD1 is an oncogene in ovarian cancer.

The signaling pathways that regulate the stemness maintenance and survival of CSCs have become targets for cancer therapy. Previous studies have established that the activated JNK signaling pathway contributes to CSC maintenance^30^; however, the molecular mechanism by which JNK is activated in OCSCs is not well understood. Our results indicated that a high level of OTUD1 drives JNK pathway activation, suggesting that OTUD1 might serve as a biomarker for JNK inhibition-based OCSC-targeted therapy. In addition, numerous JNK inhibitors have been developed, but very few such drugs are available for clinical use; one of the major reasons for this lack is their intolerable cytotoxicity. We found that the FDA-approved drug ibrutinib, which has been used for treating relapsed/refractory nongerminal center B-cell-like diffuse large B-cell lymphoma (DLBCL) in clinical trials^50^, was recently reported to be an MKK7 inhibitor^38^, and we found that it effectively suppresses OSCS and tumorigenesis in OTUD1^high^ ovarian cancer and sensitizes cancer cells to chemotherapy. Given that CSCs are mostly arrested in G0 phase and are relatively static, thus evading the cytotoxic effect of chemotherapeutic drugs^51^, our work may provide a rationale for developing a safe and reliable combination treatment for ovarian cancer.

In summary, our work suggests that aggresome formation in tumor cells could function as a signaling hub and that aggresome-based therapy has translational potential for patients with OTUD1^high^ ovarian cancer. A previous study showed that a conserved aggregation-nucleating sequence within the hydrophobic core of the DNA-binding domain of p53 becomes exposed after mutation and is responsible for mutant p53 self-assembly. Moreover, a peptide designed to inhibit p53 amyloid formation to rescue p53 functions and reduce tumorigenesis and metastasis was also reported, prompting us to explore strategies such as targeting OTUD1 and abrogating its aggresome formation, which warrants further investigation.

## Methods

### Study approval

All the animal used in this study were evaluated and approved by the Experimental Animal Welfare Ethics Committee, Zhongnan Hospital of Wuhan University (license no. ZN2022255). Tumor tissues from patients were obtained from the Shanghai Outdo Biotech Company. The use of tissue microarray for research purposes was approved by the Ethics Committee of Shanghai Outdo Biotech Company (license no. YBM-05-02). Written informed patient consent was obtained prior to the commencement of the study. All procedures performed in the study were in accordance with the Declaration of Helsinki.

### Cell culture

293T (SCSP-502), SKOV3 (TCHu185), CAOV3 (SCSP-570), and OVCAR3 (TCHu228) cell lines originated from the National Collection of Authenticated Cell Cultures (Shanghai, China). OVCAR8 cell line was a gift from Professor Chaoyang Sun (Tongji Hospital, Tongji Medical College, Huazhong University of Science and Technology). Cell lines were authenticated by STR profiling and verified to be mycoplasma negative using the MycoBlue Mycoplasma Detector kit (Vazyme, China, #D101-01). 293T, SKOV3 and CAOV3 cell lines were cultured in Dulbecco’s modified Eagle’s medium (DMEM, Hyclone, America, #SH30243.01) supplemented with 10% fetal bovine serum (FBS, AusgeneX, Australia, #FBS500-S). OVCAR8 cell line was cultured in RPMI-1640 (Hyclone, America) supplemented with 10% FBS. OVCAR3 cell line was cultured in RPMI-1640 supplemented with 20% FBS. SKOV3, CAOV3, OVCAR8 and OVCAR3 cell line were cultured in DMEM/F12 (Gibco, America, #11320033) supplemented with 2% B-27 supplement (Gibico, America, #12587010), 20 ng/mL epidermal growth factor (EGF, Solarbio, China, P00033), 20 ng/mL basic fibroblast growth factor (bFGF, Solarbio, China, #P00032), and 5 μg/mL insulin (Solarbio, China, #I8040) for the tumor sphere formation assay.

### Lentiviral transduction

Lentiviruses were produced by transfecting 293T cells with sgRNA-targeting plasmids, phage plasmids, or pCW-EGFP plasmids, along with the packaging plasmids pMD2.G and psPAX2. The cell supernatants were harvested 48 h after transfection using Lipofectamine 2000 reagent (Invitrogen, America, #11668019) and were used to infect SKOV3, OVCAR8, CAOV3 or 293T cells, respectively. To obtain stable cell lines, cells were infected at low confluence (20%) for 24 h with lentiviral supernatants diluted 1:1 with normal culture medium in the presence of 5 ng/mL of polybrene (Solarbio, China, #H8761). Forty-eight hours after infection, stable cell lines were obtained by using puromycin (Solarbio, China, #P8230) for one week. The maintenance concentration of puromycin was 2 µg/mL for SKOV3 cells, OVCAR3, OVAR8, and CAOV3 cells.

### Vectors and plasmids production

Wild type, truncating mutation and deletion mutation of OTUD1, as well as wild type, truncating mutation of ASK1, were cloned into pHAGE for mammalian expression with different tags. Additionally, the pET28a-mEGFP-OTUD1, pET28a-mEGFP -Δ105–164 constructs were cloned for protein purification. OTUD1 C320A point mutations were generated by site-directed mutagenesis PCR from the pHAGE-Flag-OTUD1 plasmids.

### Antibodies

Anti-OTUD1 (HPA038504, 1:1000) and Anti-OTUD1 (HPA038503, 1:200) were purchased from Atlas Antibodies. Anti-OTUD1 (29921-1-AP, 1:200), anti-ASK1 (67072-1-Ig, 1:1000), Anti-ERK (11257-1-AP, 1:1000), anti-p38 (114064-1-AP, 1:1000), anti-JNK, (66210-1-Ig, 1:1000), anti-HSP70, (10995-1-AP, 1:200), anti-P62 SQSTM1, (18420-1-AP, 1:200), anti-GAPDH, (60004-1-Ig, 1:3000), and anti-Flag tag, (20543-1-AP, 1:1000) were purchased from Proteintech. Anti-phospho-p44/42 MAPK (Erk1/2) Thr202/Tyr204) (#9101, 1:1000), anti-phospho- p38 MAPK (Thr180/Tyr182) (#9211, 1:1000), anti-SAPK/JNK (Phospho-Thr183/Tyr185) (81E11) (#4668, 1:1000), anti-c-Jun (Phospho-Ser73) (D47G9), (#3270, 1:1000), and anti-c-Jun (#9165, 1:1000) were purchased from Cell Signaling Technology. Anti-ASK1 (380952, 1:1000) was purchased from. Anti-ASK1 (Phospho-Thr838) (orb335764, 1:1000) was purchased from Biorbyt. anti-HA tag (2063, 1:1000) and anti-Myc tag (2097, 1:1000) were purchased from DiaAn Biotech. Goat anti-Rabbit IgG H&L (Alexa Fluor® 488) (ab15007, 1:200), Goat anti-Rabbit IgG H&L (Alexa Fluor 555) (ab150078, 1:200), APC Mouse Anti-Human CD44(G44-26) (559942, 1:50), and FITC Mouse Anti-Human CD133(W6B3C1) (567029, 1:50) were purchased from BD Pharmingen. Goat anti- Rabbit IgG H&L (HRP) (BF03008, 1:5000) and Goat anti-Mouse IgG H&L (HRP) (BF03001, 1:5000) were purchased from Biodragon,

### CRISPR/Cas9 generation of OTUD1 Knockout

The CRISPR-Cas9-based editing of OTUD1 was generated by cloning the single guide RNAs (sgRNAs) into a lentiCRISPR V2 vector, which encodes both Cas9 and a sgRNA of interest. The sgRNA design and cloning were conducted according to the general cloning protocols of the Feng Zhang lab (https://zlab.bio/guide-design-resources) and the sequences of sgRNAs were listed in Supplementary Table 1. All the constructs were validated by DNA sequencing and details of the plasmid constructions are available upon request. Cells were plated on 6-well plates the day before the transfection. Transfection was performed using Lipofectamine 2000 (Invetrogen, # 11668019) according to the manufacturer’s instructions. A total of 2 μg of sgRNA plasmid DNA, 1 μg of plasmid pMD2.G, and 1 μg of plasmid psPAX per dish with 2:3 Lipofectamine 2000 ratio were used. Supernatant containing virus was collected 48 h after transfection.

### RNA extraction and qRT-PCR

Total RNA was extracted using TRIzol (Invitrogen, America, #15596018) and thenreverse transcribed using All-in-One cDNA synthesis SuperMix (Bimake, America, #B24403). qPCR was carried out using SYBR Green (Bimake, America, #B21802) using CFX Connect Real Time PCR Detection System (Bio-Rad, America). All target gene-expression levels were normalized to GAPDH. The primer sequences are provided in Supplementary Table 2.

### Western blotting

Cells were lysed with 100 μL RIPA lysis buffer (Beyotime, China, #P0013K) containing protease inhibitor mixture (MedChemExpress, America, #HY-K0010) for 30 min at 4 °C followed by ultrasonic-assisted extraction. The cell lysates were then sonicated and centrifuged to obtain the supernatant. 20–40 μg of proteins were separated by SDS-PAGE. Primary antibodies and secondary anti-mouse or anti-rabbit antibodies conjugated to horseradish peroxidase were then incubated with the blots. Protein bands were visualized on X-ray film (Sigma, America) using an ECL recognition system (Vazyme, China, #SQ201). Antibodies used are shown in Supplementary Table 3.

### Co-immunoprecipitation

The cell pellet was resuspended in ice-cold cell lysis buffer and incubated on ice for 30 min. The cells were then sonicated in an ice bath and centrifuged at 12,000 × *g* at 4 °C for 10 min. The supernatant was transferred to a fresh tube, and the remaining lysates were incubated with the lysis buffer treated at room temperature for 40 min. Immunoprecipitation was performed with anti-FLAG magnetic beads (MedChemExpress, America, #HY-K0207) or with anti-HA magnetic beads (MedChemExpress, America, #HY-K0201) for 6–8 h at 4 °C. Proteins immobilized on the beads were eluted with 2× loading buffer and heated at 100 °C for 10 min. Protein samples were resolved by SDS-PAGE using 8–12% acrylamide gels, transferred to nitrocellulose (NC) membranes, and immunoblotted using the indicated antibodies.

### Gel-shift assay

Cells were lysed with RIPA lysis buffer (Beyotime, China, #P0013K) containing protease inhibitor mixture (MedChemExpress, America, #HY-K0010) for 30 min at 4 °C, followed by ultrasonic-assisted extraction. The cell lysates were sonicated and centrifuged to obtained the supernatant. Part of the supernatants were collected and heated at 100 °C. The heated and unheated proteins were then separated by SDS-PAGE. Coomassie Brilliant Blue dye was used to stain the SDS-PAGE gels to visualize proteins.

### RNA sequencing

Total RNA was extracted from different groups using TRIzol (Invitrogen, America, #15596018). The quality of RNA was measured, and RNA sequencing were performed by Novogene Biotech company (Beijing, China). The cDNA libraries were sequenced using the Illumina novaseq-PE150 sequencing platform at Novogene Biotech company (Beijing, China). For the downstream analyses with RNA-Seq data, the Featurecount software was used to quantitatively analyze the gene level. The EdgeR software was used to analyze the differential expression of gene in each sample. To obtain the Kyoto Encyclopedia of Genes and Genomes (KEGG) result, the KOBAS softewarewas employed.

### CCK-8 assay

Cell viability was measured using the CCK-8 kit (MedChemExpress, America, #HY-K0301). Cells were seeded at 30–50% density in 96-well plates. Then, after 0 h, 24 h, 48 h, and 72 h of incubation, all medium was removed and replaced with the fresh medium containing CCK-8 reagent for 1 h.Tthe plates were then read by micro-plate reader (BioTeK, America) at 450 nm.

### Colony formation assay

Ovarian cancer cells were seeded into 6-well plates. When the cells were adherent, they were stimulated with appropriate inhibitors, such as SP600125 (MedChemExpress, America, #HY-12041), ibrutinib (MedChemExpress, America, # HY-10997), IN-8(MedChemExpress, America, #HY13319), selonsetib (MedChemExpress, America, #HY18938), T-5224 (MedChemExpress, America, #HY-12270) and cisplatin (MedChemExpress, America, # HY-17394) or a combination of both for 2-3 weeks. Then, the medium was removed and fixation solution was added into the plates at room temperature for 10 min. The colony cells were soaked in 0.5% crystal violet solution and incubated at room temperature for 2 h after removing the fixation solution.

### Transwell invasion assays

Thaw the stock 10 mL Matrigel® (Corning, America, #356234) overnight at 4 °C by placing inside at cold room. Dilute Matrigel at a 1:8 ratio with chilled serum-free growth medium before coating. 100 μL the chilled diluted Matrigel was placed directly onto the center of the Transwell insert. Place the plates in a humidified incubator at 37 °C for 60 min to allow gelling. Meanwhile, a proper density of cancer cells in 200 ml serum-free growth medium were added to the upper chamber of replicate and 500 μL serum-containing medium was added to the lower chamber. The plate was incubated for 24–72 h at 37 °C. The inserts were stained with crystal violet and were observed under microscope (Olympus, America, IX53 + DP80) for identification.

### Soft agar colony formation assay

1% noble agar and 0.6% noble agar were prepared. The bottom layer was obtained by mixing 1% noble agar with equal volume of 2X cell culture medium. Once the lower layer of agar solidified, the upper layer containing with cells were plated onto lower layer. The cells and agar mixture were placed into a 37 °C cell culture incubator for around 21 days. Formed colonies were stained with 0.01% crystal violet and photographs of wells were obtained by using microscope (Olympus, America, IX53 + DP80).

### Tumor sphere formation assays

Single-cell suspensions of ovarian cancer cells (4 × 10^2^ cells/mL) were plated on ultra-low attachment 24 wells plates (Corning, America) and cultured in phenol red-free DMEM/F12 (Gibco, America) containing B27 supplement (Gibico, America, #12587010) and 20 ng/mL epidermal growth factor (EGF, Solarbio, China, #P00033), 20 ng/mL basic fibroblast growth factor (bFGF, Solarbio, China, #P00032), and 5 μg/mL insulin (Solarbio, China, #I8040). Tumorsphere were visualized under a phase-contrast microscope (Olympus, America, IX53 + DP80).

### Tissue microarray immunohistochemistry assay

Tissue chips containing with 4 cases of low grade ovarian serous carcinoma and 54 cases of high grade ovarian serous carcinoma were obtained from Shanghai Outdo Biotech Company (#HOvaC070PT01). The slides were deparaffinized in xylene for 10 min and then rinsed with distilled water. After that, the slides were treated with citrate buffer and microwaved for 10 min. Once cooled, the slides were rinsed with TBST for 3 min each. Subsequently, the slides were exposed to 1% H_2_O_2_ for 10 min, followed by another rinse with distilled water. The slides were then blocked in 5% BSA for 1 h. The tissues chips were incubated with the primary antibody overnight at 4 °C, and then in the secondary antibody for 1 h, as listed in Supplementary Table 2. Finally, the indicated proteins were visualized using the microscope (Olympus, America, #IX53 + DP80). The glass slides were scanned using a panoramic scanning instrument, and the staining results were analyzed using CaseViewer software. The used of tissue microarray for research purposed was approved by the Ethics Committee of Shanghai Outdo Biotech Company (license no. YBM-05-02).

### Immunofluorescence assay

In the case of cells, ovarian cancer cells or 293T were seeded on cover glass. The cells were fixed by 4% paraformaldehyde and permeabilized with 0.2% triton X-100. The cover glass was then rinsed and blocked in 5% BSA for 10 min. Subsequently, the glass slides were incubated with primary antibodies (listed in Supplementary Table 2) with different reactivity overnight at 4 °C. The slides were then incubated in the fluorescent-dye conjugated secondary antibodies, washed with PBST, and stained with DAPI (4’6-diamidino-2-phenylindole, Beyotim, China, #P0131) for 5 min for nucleus labeling. After air-drying for 10 min, the samples were observed using a confocal microscope (Leica, Germany, #TCS SP8).

In the case of tissues, firstly, formalin-fixed paraffin-embedded tissue sections were dewaxed and rehydrated. Next, the tissue sections were incubated with the primary antibody for 8 h at room temperature following heat-induced antigen retrieval. Finally, the tissue sections were incubated with a fluorophore-conjugated secondary antibody according to the manufacturer’s instructions. The tissue sections were observed using confocal microscope (Leica, Germany, #TCS SP8).

### Fluorescence recovery after photo-bleaching measurements

Confocal fluorescence measurements were taken using a Leica inverted microscope with a confocal laser scanning module. EGFP fluorescence was monitored at 488 nm. Each Fluorescence Recovery After Photo-Bleaching measurements (FRAP) assay started with a baseline control image of 5 cells, followed by 1 s photobleaching of the region of interest using high-intensity 488 nm illumination with >90% EGFP fluorescence. Typically, images were recorded at normal laser intensity after photobleaching.

### Xenograft assay

5-week-old female BALB/c nude mice were used as wild-type and acquired from Wuhan Wanqianjiaxing Biotechnology Co., Ltd (China, Wuhan). All animals were maintained in a specific pathogen-free environment and housed with no more than five animals per cage under 12 light/12 dark cycle, temperatures of 22 ± 2 °C with 50 ± 10% humidity.

In the xenotransplantation limiting dilution assay, different stable SKOV3 cells (empty vector, OTUD1^WT^, OTUD1^△105–164^) were prepared for xenograft assay. 5-week-old female BALB/c nude mice were acquired from Wuhan Wanqianjiaxing Biotechnology Co., Ltd (China, Wuhan). After a week of adjustable feeding, the mice were divided into 3 groups (18 mice each group). Cells were serially diluted to obtain the following final cell concentrations: dilution group 1 was 1 × 10^4^ cells/each; dilution group 2 was 1 × 10^5^ cells/each; dilution group 3 was 1 × 10^6^ cells/each. Different stable SKOV3 cells were injected into the left or right armpit flank subcutaneously. Tumor incidence and size were monitored within the indicated days after injection. All mice are euthanized by cervical dislocation after deep anesthesia. After all mice were sacrificed, subcutaneous tumors were further tissue analyzed. The permitted maximal tumor size did not exceed 1.5 cm at the largest diameter.

For SKOV3 cells and OVCAR3 cells, 5-week-old female BALB/c nude female mice (Wuhan Wanqianjiaxing Bioscience (China)) were used. At 6th week, SKOV3-sgCtrl or SKOV3-sgOTUD1 cells were randomly injected into the left or right armpit flank subcutaneously, 5 × 10^6^ cells per needle. At 6th week, OVCAR3-sgCtrl or OVCAR3-sgOTUD1 cells were randomly injected into the left or right armpit flank subcutaneously, 1 × 10^7^ cells per needle. Tumor incidence and size were monitored within the indicated days after injection. The permitted maximal tumor size did not exceed 1.5 cm at the largest diameter. All mice are euthanized by cervical dislocation after deep anesthesia. After all mice were sacrificed, subcutaneous tumors were further tissue analyzed.

For SKOV3 cells, 5-week-old female BALB/c nude female mice (Wuhan Wanqianjiaxing Bioscience (China)) were used. At 6th week, 1 × 10^7^ SKOV3 cells were randomly injected into the left or right armpit flank subcutaneously. On day 14 post-injection, the mice were randomly divided into 2 groups and treated with ibrutinib (25 mg/kg, MedChemExpress, America, #HY-10997) or vehicle control (20% SBE-β-CD in saline) each other day by intraperitoneal injection. Tumor incidence was monitored within indicated days after injection. All mice are euthanized by cervical dislocation after deep anesthesia. The tumor tissues were harvested and used to detect the levels of OTUD1 related proteins. For OVCAR3 cells, the same method was used for xenograft assay in BALB/c nude female mice, and the inoculation site was the left or right armpit flank subcutaneously. On day 45 after injection, the mice were randomly divided into 2 groups and treated with ibrutinib (25 mg/kg, MedChemExpress, America, #HY-10997) or vehicle control (20% SBE-β-CD in saline) each other day by intraperitoneal injection. All mice are euthanized by cervical dislocation after deep anesthesia. Tumor incidence was monitored on indicated days after injection. For OVCAR8 cells, 1 × 10^7^ OVCAR8 cells were injected subcutaneously into 6-week-old female BALB/c nude female mice, and the inoculation site was the left or right armpit flank subcutaneously. On day 24 post-injection, the mice were randomly divided into 2 groups and treated with ibrutinib (25 mg/kg, MedChemExpress, America, #HY-10997) or vehicle control (20% SBE-β-CD in saline) each other day by intraperitoneal injection. Tumor incidence and size were monitored within the indicated days after injection. All mice are euthanized by cervical dislocation after deep anesthesia. After all mice were sacrificed, subcutaneous tumors were further tissue analyzed. The permitted maximal tumor size did not exceed 1.5 cm at the largest diameter.

### Statistics

All graphs in this study were generated by GraphPad Prism 9. Statistical tests were shown in the Figure legends. Data are presented as mean ± standard error (SD) unless otherwise stated. Two-tailed Student’s *t* test (unpaired), one-way ANOVA test or two-way ANOVA test was used to assess the significance of the experiments. *P* value < 0.05 was considered to be statistically significant.

### Reporting summary

Further information on research design is available in the Nature Portfolio Reporting Summary linked to this article.

### Supplementary information

Supplementary Information

Peer Review File

Description of Additional Supplementary Files

Supplementary Data 1-2

Reporting Summary

### Source data

Source Data

## Data Availability

Publicly available datasets reported in this paper are The Cancer Genome Atlas^52^ (TCGA, https://portal.gdc.cancer.gov), Kaplan–Meier Plotter^53,54^ (KM-plotter, https://kmplot.com/analysis/), Gene Expression Profiling Interactive Analysis^55^ (GEPIA, http://gepia.cancer-pku.cn/), AlphaFold Protein Structure Database^56,57^ (https://alphafold.ebi.ac.uk/entry/Q5VV17). Publicly available design tool in this study is Zhang lab Website Guide Design Tools^58^ (http://guides.sanjanalab.org/#/). An early study that rs11782652, rs1243180, and rs757210 were identified as susceptibility loci for ovarian cancer was cited^26^. The RNA-seq data of floating spheres and floating differentiated cells generated in this study are deposited in the GEO database under accession code GSE232783. The RNA-seq data of SKOV3 cells engineered to express sgNC or sgOTUD1 generated in this study are deposited in the GEO database under accession code GSE232786. The remaining data are available within the Article, Supplementary Information or Source Data file. Source data are provided with this paper.

## References

[1] Domcke S, Sinha R, Levine DA, Sander C, Schultz N (2013). Evaluating cell lines as tumour models by comparison of genomic profiles. Nat. Commun..

[2] Baek MH (2022). Secondary Cytoreductive Surgery in Platinum-Sensitive Recurrent Ovarian Cancer: A Meta-Analysis. J. Clin. Oncol..

[3] Wilczynski JR, Wilczynski M, Paradowska E (2022). Cancer Stem Cells in Ovarian Cancer-A Source of Tumor Success and a Challenging Target for Novel Therapies. Int. J. Mol. Sci..

[4] Abubaker K (2013). Short-term single treatment of chemotherapy results in the enrichment of ovarian cancer stem cell-like cells leading to an increased tumor burden. Mol. Cancer.

[5] Chen LY (2022). Epigenomic Profiling of Epithelial Ovarian Cancer Stem-Cell Differentiation Reveals GPD1 Associated Immune Suppressive Microenvironment and Poor Prognosis. Int. J. Mol. Sci..

[6] Wesley T, Berzins S, Kannourakis G, Ahmed N (2021). The attributes of plakins in cancer and disease: perspectives on ovarian cancer progression, chemoresistance and recurrence. Cell Commun. Signal.

[7] Li Y (2021). ZEB2 facilitates peritoneal metastasis by regulating the invasiveness and tumorigenesis of cancer stem-like cells in high-grade serous ovarian cancers. Oncogene.

[8] Wen Y (2021). EZH2 activates CHK1 signaling to promote ovarian cancer chemoresistance by maintaining the properties of cancer stem cells. Theranostics.

[9] Cui T (2018). DDB2 represses ovarian cancer cell dedifferentiation by suppressing ALDH1A1. Cell Death Dis..

[10] Munoz-Galvan S, Carnero A (2020). Targeting Cancer Stem Cells to Overcome Therapy Resistance in Ovarian Cancer. Cells.

[11] Xiang T (2015). Interleukin-17 produced by tumor microenvironment promotes self-renewal of CD133+ cancer stem-like cells in ovarian cancer. Oncogene.

[12] Silva IA (2011). Aldehyde dehydrogenase in combination with CD133 defines angiogenic ovarian cancer stem cells that portend poor patient survival. Cancer Res..

[13] Zong X, Nephew KP (2019). Ovarian Cancer Stem Cells: Role in Metastasis and Opportunity for Therapeutic Targeting. Cancers.

[14] Wang Y (2014). Epigenetic targeting of ovarian cancer stem cells. Cancer Res..

[15] Wei X (2010). Mullerian inhibiting substance preferentially inhibits stem/progenitors in human ovarian cancer cell lines compared with chemotherapeutics. Proc. Natl Acad. Sci. USA.

[16] Hu L, McArthur C, Jaffe RB (2010). Ovarian cancer stem-like side-population cells are tumourigenic and chemoresistant. Br. J. Cancer.

[17] Niu N, Mercado-Uribe I, Liu J (2017). Dedifferentiation into blastomere-like cancer stem cells via formation of polyploid giant cancer cells. Oncogene.

[18] Le PN, McDermott JD, Jimeno A (2015). Targeting the Wnt pathway in human cancers: Therapeutic targeting with a focus on OMP-54F28. Pharmacol. Therapeut..

[19] Cascio S (2021). Cancer-associated MSC drive tumor immune exclusion and resistance to immunotherapy, which can be overcome by Hedgehog inhibition. Sci. Adv..

[20] Jia YF, Wang YS, Xie JW (2015). The Hedgehog pathway: role in cell differentiation, polarity and proliferation. Arch. Toxicol..

[21] Diaz-Padilla I (2015). A phase II study of single-agent RO4929097, a gamma-secretase inhibitor of Notch signaling, in patients with recurrent platinum-resistant epithelial ovarian cancer: A study of the Princess Margaret, Chicago and California phase II consortia. Gynecologic Oncol..

[22] Seino M (2014). Requirement of JNK signaling for self-renewal and tumor-initiating capacity of ovarian cancer stem cells. Anticancer Res..

[23] Seino M (2016). Time-staggered inhibition of JNK effectively sensitizes chemoresistant ovarian cancer cells to cisplatin and paclitaxel. Oncol. Rep..

[24] Lee HG (2017). Salinomycin reduces stemness and induces apoptosis on human ovarian cancer stem cell. J Gynecol. Oncol..

[25] Duan D (2011). Electrophysiological characterization of NSCs after differentiation induced by OEC conditioned medium. Acta Neurochir..

[26] Pharoah PDP (2013). GWAS meta-analysis and replication identifies three new susceptibility loci for ovarian cancer. Nat. Genet..

[27] Tsai SY, Huang YL, Yang WH, Tang CH (2012). Hepatocyte growth factor-induced BMP-2 expression is mediated by c-Met receptor, FAK, JNK, Runx2, and p300 pathways in human osteoblasts. Int. Immunopharmacol..

[28] Binato R (2021). NRIP1 is activated by C-JUN/C-FOS and activates the expression of PGR, ESR1 and CCND1 in luminal A breast cancer. Sci. Rep..

[29] Xu ZY (2021). Endothelial deletion of SHP2 suppresses tumor angiogenesis and promotes vascular normalization. Nat. Commun..

[30] Semba T (2020). JNK Signaling in Stem Cell Self-Renewal and Differentiation. Int. J. Mol. Sci..

[31] Girnius N, Edwards YJ, Garlick DS, Davis RJ (2018). The cJUN NH(2)-terminal kinase (JNK) signaling pathway promotes genome stability and prevents tumor initiation. Elife.

[32] Zhang P (2020). SH3RF3 promotes breast cancer stem-like properties via JNK activation and PTX3 upregulation. Nat. Commun..

[33] Xie X (2017). c-Jun N-terminal kinase promotes stem cell phenotype in triple-negative breast cancer through upregulation of Notch1 via activation of c-Jun. Oncogene.

[34] Ichijo H (1997). Induction of apoptosis by ASK1, a mammalian MAPKKK that activates SAPK/JNK and p38 signaling pathways. Science.

[35] Webb JL, Ravikumar B, Rubinsztein DC (2004). Microtubule disruption inhibits autophagosome-lysosome fusion: implications for studying the roles of aggresomes in polyglutamine diseases. Int. J. Biochem. Cell Biol..

[36] Yoon CH (2012). c-Jun N-terminal kinase has a pivotal role in the maintenance of self-renewal and tumorigenicity in glioma stem-like cells. Oncogene.

[37] Harrison SA (2020). Selonsertib for patients with bridging fibrosis or compensated cirrhosis due to NASH: Results from randomized phase III STELLAR trials. J. Hepatol..

[38] Schroder M (2020). Catalytic Domain Plasticity of MKK7 Reveals Structural Mechanisms of Allosteric Activation and Diverse Targeting Opportunities. Cell Chem. Biol..

[39] Olzmann JA, Li L, Chin LS (2008). Aggresome formation and neurodegenerative diseases: Therapeutic implications. Curr. Med. Chem..

[40] Goldberg AL (2003). Protein degradation and protection against misfolded or damaged proteins. Nature.

[41] Watanabe S (2020). Aggresome formation and liquid-liquid phase separation independently induce cytoplasmic aggregation of TAR DNA-binding protein 43. Cell Death Dis..

[42] Yehia M (2019). Association of Aggresomes with Survival Outcomes in Pediatric Medulloblastoma. Sci. Rep..

[43] Li JZ (2022). p53 amyloid aggregation in cancer: function, mechanism, and therapy. Exp. Hematol. Oncol..

[44] Petronilho EC (2021). Phase separation of p53 precedes aggregation and is affected by oncogenic mutations and ligands. Chem. Sci..

[45] Nijman SM (2005). A genomic and functional inventory of deubiquitinating enzymes. Cell.

[46] Wu B (2022). The deubiquitinase OTUD1 inhibits colonic inflammation by suppressing RIPK1-mediated NE-kappa B signaling. Cell Mol. Immunol..

[47] Zhang Z (2017). Breast cancer metastasis suppressor OTUD1 deubiquitinates SMAD7. Nat. Commun..

[48] Song J (2021). The deubiquitinase OTUD1 enhances iron transport and potentiates host antitumor immunity. Embo. Rep..

[49] Fan GL (2023). The deubiquitinase OTUD1 noncanonically suppresses Akt activation through its N-terminal intrinsically disordered region. Cell Rep..

[50] Wilson WH (2015). Targeting B cell receptor signaling with ibrutinib in diffuse large B cell lymphoma. Nat. Med..

[51] Issa ME (2017). Epigenetic strategies to reverse drug resistance in heterogeneous multiple myeloma. Clin. Epigenetics.

[52] TCGA. (2011). Integrated genomic analyses of ovarian carcinoma. Nature.

[53] Győrffy B (2023). Discovery and ranking of the most robust prognostic biomarkers in serous ovarian cancer. Geroscience.

[54] Nagy Á, Munkácsy G, Győrffy B (2021). Pancancer survival analysis of cancer hallmark genes. Sci. Rep..

[55] Tang Z (2017). GEPIA: a web server for cancer and normal gene expression profiling and interactive analyses. Nucleic Acids Res..

[56] Jumper J (2021). Highly accurate protein structure prediction with AlphaFold. Nature.

[57] Varadi M (2022). AlphaFold Protein Structure Database: massively expanding the structural coverage of protein-sequence space with high-accuracy models. Nucleic Acids Res..

[58] Canver MC (2018). Integrated design, execution, and analysis of arrayed and pooled CRISPR genome-editing experiments. Nat. Protoc..

