# Supplementary material for: Aggresome formation promotes ASK1/JNK signaling activation and stemness maintenance in ovarian cancer
Source: Nat Commun. 2024 Feb 13;15:1321. doi: 10.1038/s41467-024-45698-x (PMC10864366; doi:10.1038/s41467-024-45698-x)
Supplement: Supplementary file 1 — Supplementary Information [file 41467_2024_45698_MOESM1_ESM.pdf]

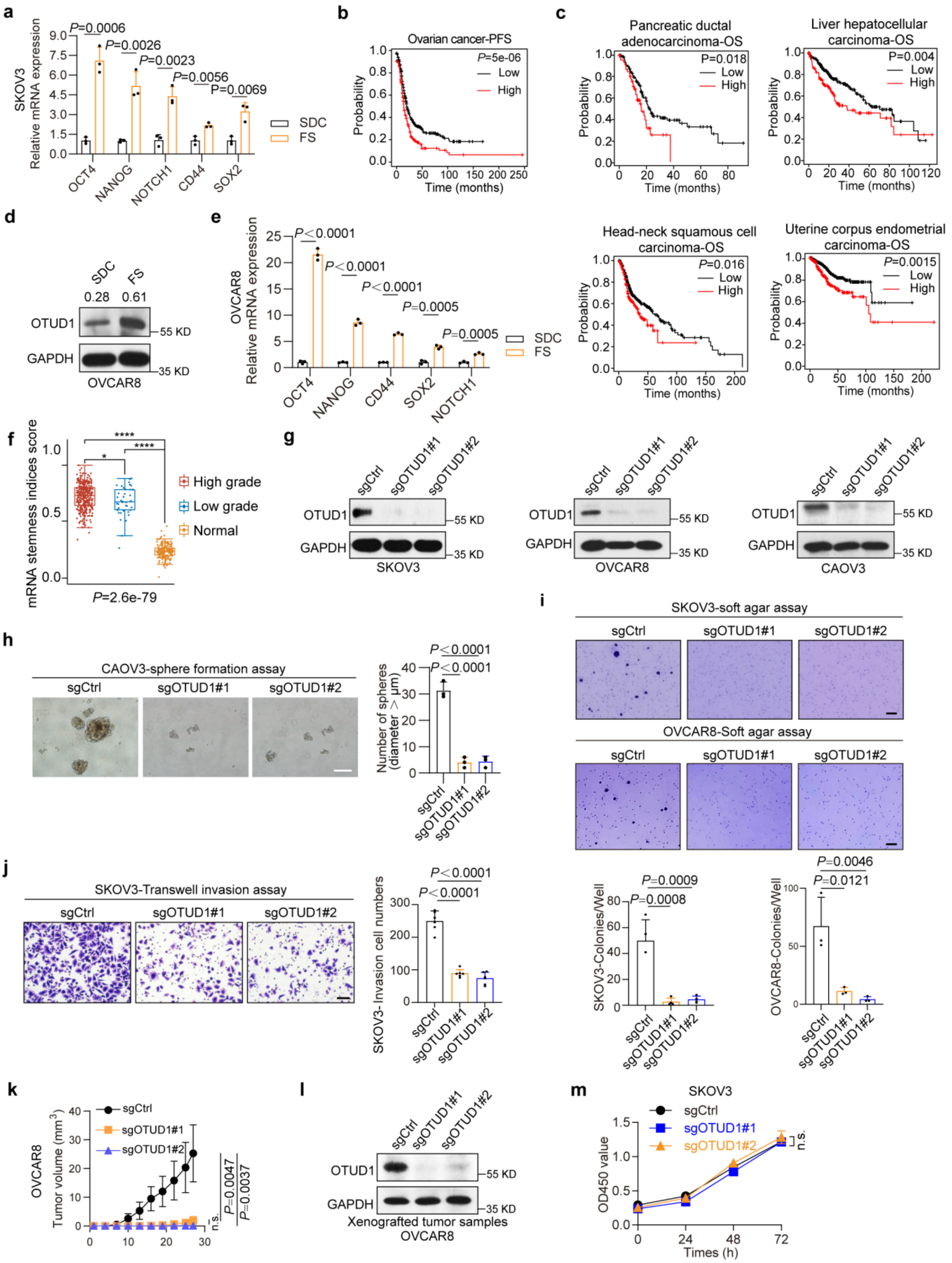

Supplementary Fig. 1 (Related to Fig. 1) OTUD1 sustains the stemness in serous

ovarian cancer.

a The expression of stemness-associated genes (ie, *NANOG*, *OCT4, NOTCH1, CD44 and SOX2*) were quantified by qPCR in the SKOV3 floating sphere and differentiated cells (*n* = 3*).*

b Kaplan–Meier survival curves show that the progression-free survival (PFS)of individuals had a negative correlation with OTUD1 expression level in ovarian cancer (https://kmplot.com/analysis/). *n* = 687 patients with ovarian cancer.

c Kaplan–Meier curves show the correlation between the overall survival (OS) of individuals and OTUD1 gene expression in several types of cancer. (https://kmplot.com/analysis/). *n* = 261 patients with pancreatic ductal adenocarcinoma. *n* = 704 patients with liver hepatocellular carcinoma. *n* = 609 patients with head-neck squamous cell carcinoma. *n* = 713 patients with uterine corpus endometrial carcinoma.

d OTUD1 protein level was detected in OVCAR8 floating spheres and the derived differentiated cells.

e The expression of stemness-associated genes (ie, *NANOG*, *OCT4, NOTCH1, CD44 and SOX2*) were quantified by qPCR in the OVCAR8 floating sphere and differentiated cells (*n* = 3*).*

f Stemness-related genes mRNA expression-based stemness index (mRNAsi) in normal (54 samples), high-grade serous ovarian cancer (323 samples), and low-grade serous ovarian cancer (43 samples) tissues were analyzed in ASSISTANT for Clinical Bioinformation (https://www.aclbi.com/static/index.html#/). The abscissa represents different groups of samples, and the ordinate represents the distribution of the mRNA score.

g OTUD1 knockout with distinct sgRNAs was validated by IB assays in SKOV3, OVCAR8 and CAOV3 cells.

h Representative images showing the effects of OTUD1 knockout on floating sphere-forming capacity in CAOV3 cell. Statistical analysis showing the effect of OTUD1 depletion on number of floating spheres in CAOV3 cell linse (*n* = 3). Scale bar, 50 μm.

i The effect of OTUD1 depletion on anchorage-independent growth ability of SKOV3 and OVCAR8 cells. Representative pictures are shown and the colonies were quantified (*n* = 3). Scale bar, 400 μm.

j Transwell assays were performed to examine the invasion ability of SKOV3 OTUD1 knockout cells or control cells. Representative pictures are shown and their invasion ability were quantified (*n* = 6). Scale bar, 20 μm.

Tumor volumes in mice injected with negative control and OTUD1 knockout OVCAR8 cells (n = 6 mice per group). Tumor sizes (**k**) were monitored and the protein level of xenografts (**l**) were analyzed by western blot.

m Cell proliferation assays for SKOV3 cells with or without OTUD1 depletion (*n* = 3*).*

P values are calculated using two-tailed unpaired Student's t test (**a, e**), one-way ANOVA (**h, I, j**) and two-way ANOVA (**k, m**). n.s., not significant. Representative of n = 3 independent experiments (**a, d, e, g, k, j, i**). Source data are provided as a Source Data file.

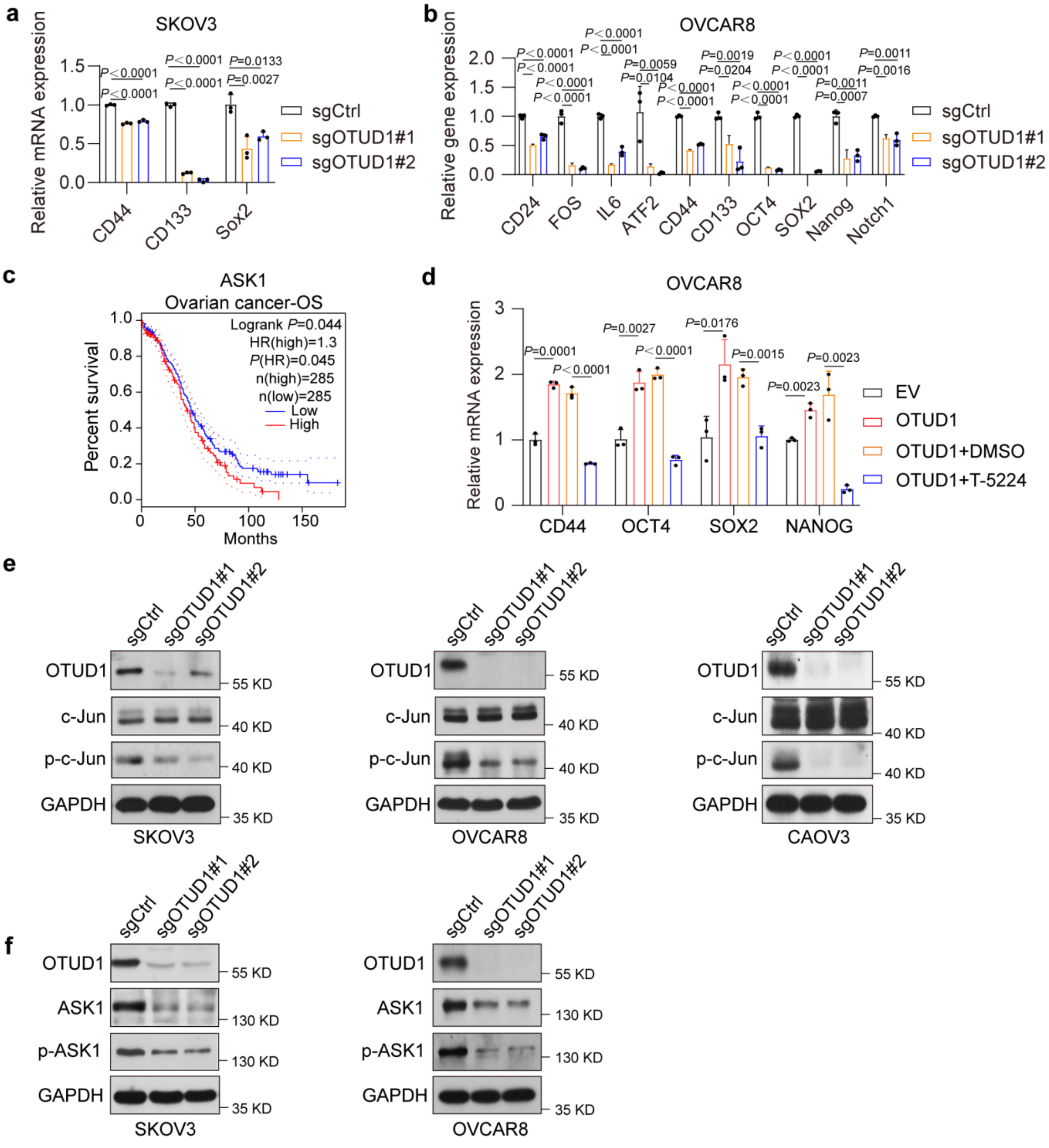

Supplementary Fig. 2 (Related to Figure 2) OTUD1 plays a role in ovarian cancer stem cell stemness and JNK/ASK1 pathway regulation,

a Expression of stemness-associated genes (ie, *SOX2, CD44* and *CD133*) in SKOV3 control or OTUD1 depteted cells were measured by qPCR (*n* = 3*).*

b The qPCR analysis of a number of CSCs markers, including *OCT4, NANOG, SOX2, CD44, CD133* and *NOTCH1* in OVCAR8 OTUD1 depleted cells (*n* = 3).

c Survival plot shows that ASK1 high expression predicts poor prognosis (overall survival, OS) in ovarian cancer (http://gepia.cancer-pku.cn/). *n* = 570 patients with ovarian cancer.

d The qPCR assay was used to confirm the expression of stemness-associated genes (ie, *CD44, OCT4, SOX2* and NANOG) in OVCAR8 cells expressing OTUD1 with T-5224 (2 μg/mL) treatment (*n* = 3).

e The specific effect of OTUD1 on the phosphorylation levels of JNK targeted molecular c-Jun were determined in SKOV3, OVCAR3 and CAOV3 OTUD1 depleted cells.

f ASK1/p-ASK1 protein level in SKOV3 or OVCAR8 OTUD1 depleted cells were detected.

P values are calculated using one-way ANOVA (**a, b**) and two-tailed unpaired Student’s t test (**d**). Representative of n = 3 independent experiments (**a, b, e, f**). Source data are provided as a Source Data file.

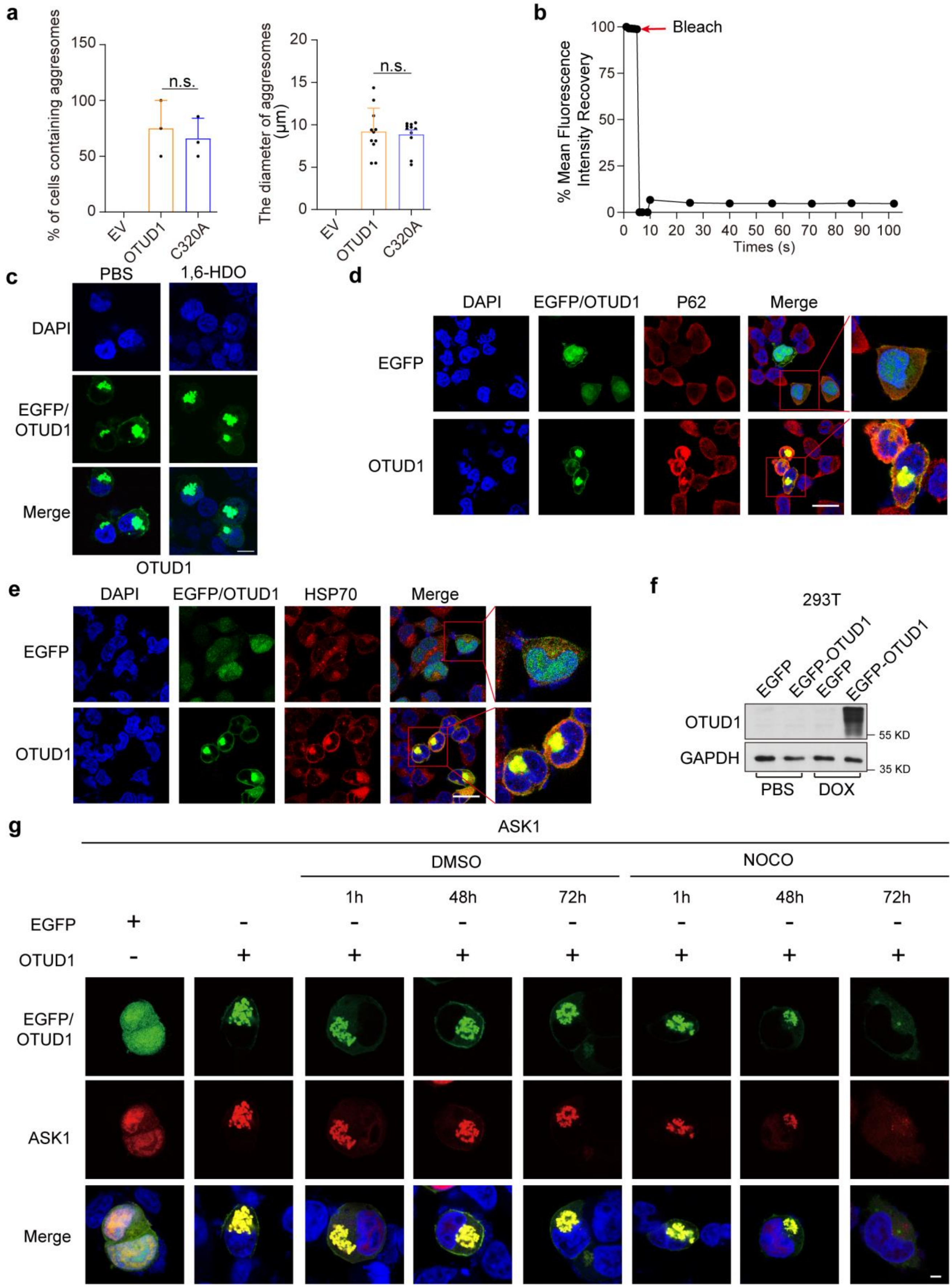

Supplementary Figure 3. (Related to Figure 3) ASK1 was sequestered into OTUD1

based aggresome.

a The percentage of aggresomes in control cells, EGFP-OTUD1-WT (WT) or EGFP-OTUD1-C320A (C320A) expressing cells that contain aggresome (*n* = 3). The diameter of aggresome in indicated cells (n = 11 EGFP positive cells examined across 3 independent experiments).

b Fluorescence recovery rate was calculated after photobleaching of OTUD1 based aggresome.

c 293T cells transfected with EGFP-OTUD1 plasmid were treated with 1,6-hexanediol (1,6-HDO) and the EGFP-OTUD1 aggregates were observed. Scale bar, 10 μm.

d-e Immunofluorescence assay was performed to observe the co-localization of EGFP-OTUD1 and indicated aggresome markers (ie, P62 and HSP70). Scale bar, 25 μm.

f Inducible OTUD1 expression was validated by IB assays in 293T cells treated with doxorubicin for 24 h.

g The effect of Nocodazole (NOCO) on aggresomes formation and ASK1 recruitment in 293T cells expressing mcherry-ASK1 and EGFP-OTUD1. Scale bar, 5 μm.

P values are calculated using two-tailed unpaired Student's t test (**a**). Representative of n = 3 independent experiments (**b, c, d, e, f, g**). Source data are provided as a Source Data file.

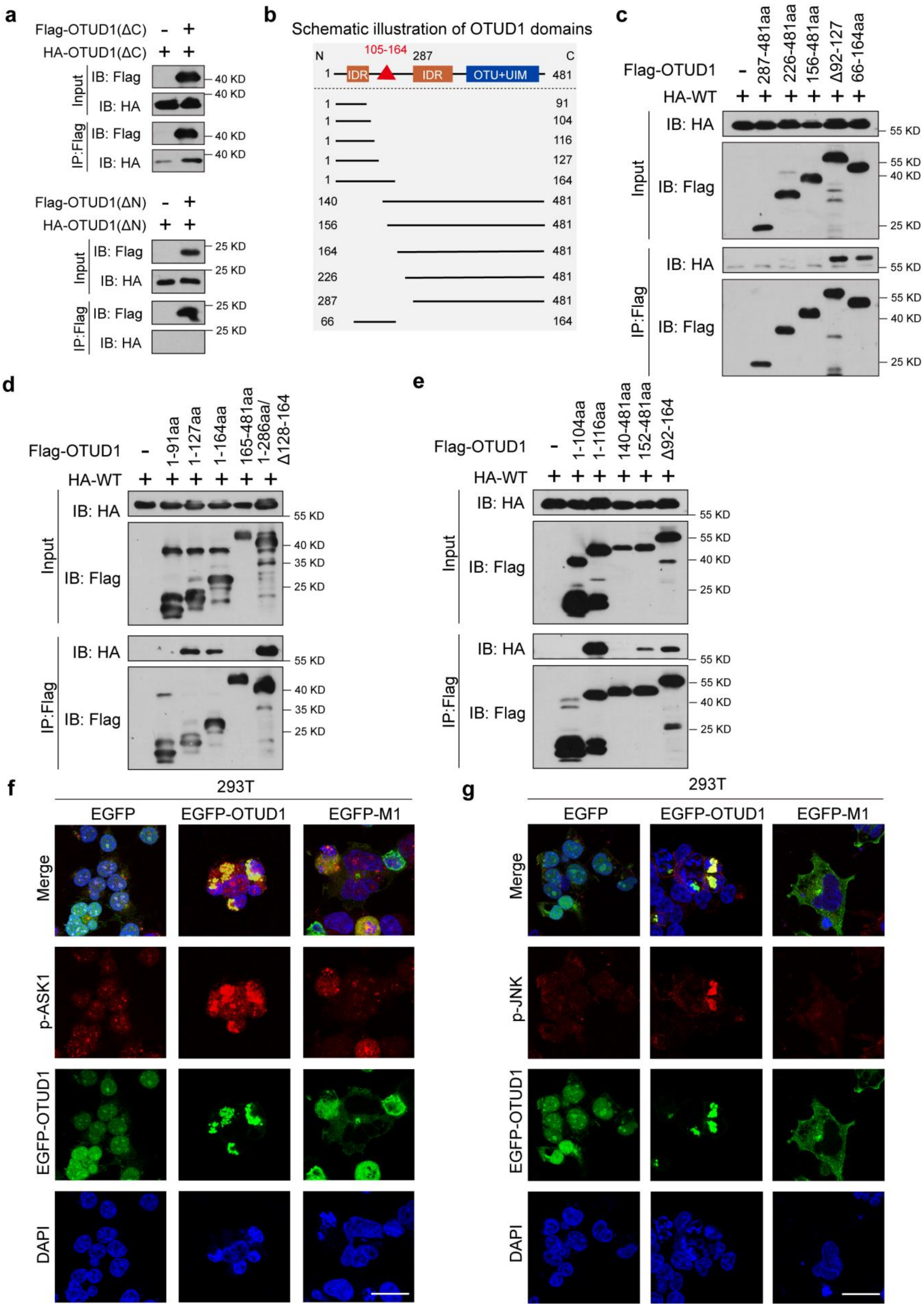
a
Flag-OTUD1(ΔC)
HA-OTUD1(ΔC)
Input
IP:Flag
IB: Flag
IB: HA
40 KD
Flag-OTUD1(ΔN)
HA-OTUD1(ΔN)
25 KD
b
Schematic illustration of OTUD1 domains
105-164
N
C
287
IDR
OTU+UIM
1
481
91
104
116
127
164
140
156
226
66
c
Flag-OTUD1
287-481aa
226-481aa
156-481aa
Δ92-127
66-164aa
HA-WT
55 KD
25 KD
d
1-91aa
1-127aa
1-164aa
165-481aa
1-286aa/Δ128-164
35 KD
e
1-104aa
1-116aa
140-481aa
152-481aa
Δ92-164
f
293T
EGFP
EGFP-OTUD1
EGFP-M1
Merge
p-ASK1
DAPI
g
p-JNK

Supplementary Figure 4. (Related to Fig. 4) The OTUD1 N-terminal intrinsic disordered region is critical for its oligomerization and activation of the downstream JNK/c-Jun pathway.

a IB analysis of input and IP products from 293T cells transfected with N-terminus (287-481 aa) or C-terminus (1-286 aa) of OTUD1 plasmid.

b Schematic illustration of OTUD1 domain and truncation constructs. OTU, ovarian tumor protease; UIM, ubiquitin-interacting motif; IDR, intrinsically disordered protein region.

c-e IB analysis of input and IP products from 293T cells transfected with full-length OTUD1 and the indicated constructs of OTUD1.

f Representative immunofluorescence images of OTUD1 and p-ASK1 protein subcellular localization in 293T cells transfected with EGFP-OTUD1 or EGFP-OTUD1-Δ105-164 (EGFP-M1) plasmid. Scale bar, 25 μm.

g Representative immunofluorescence images of of OTUD1 and p-JNK protein subcellular localization in 293T cells transfected with EGFP-OTUD1 or EGFP-OTUD1-Δ105-164 plasmid (EGFP-M1). Scale bar, 25 μm.

Representative of n = 3 independent experiments (**a, c, d, e, f, g**). Source data are provided as a Source Data file.

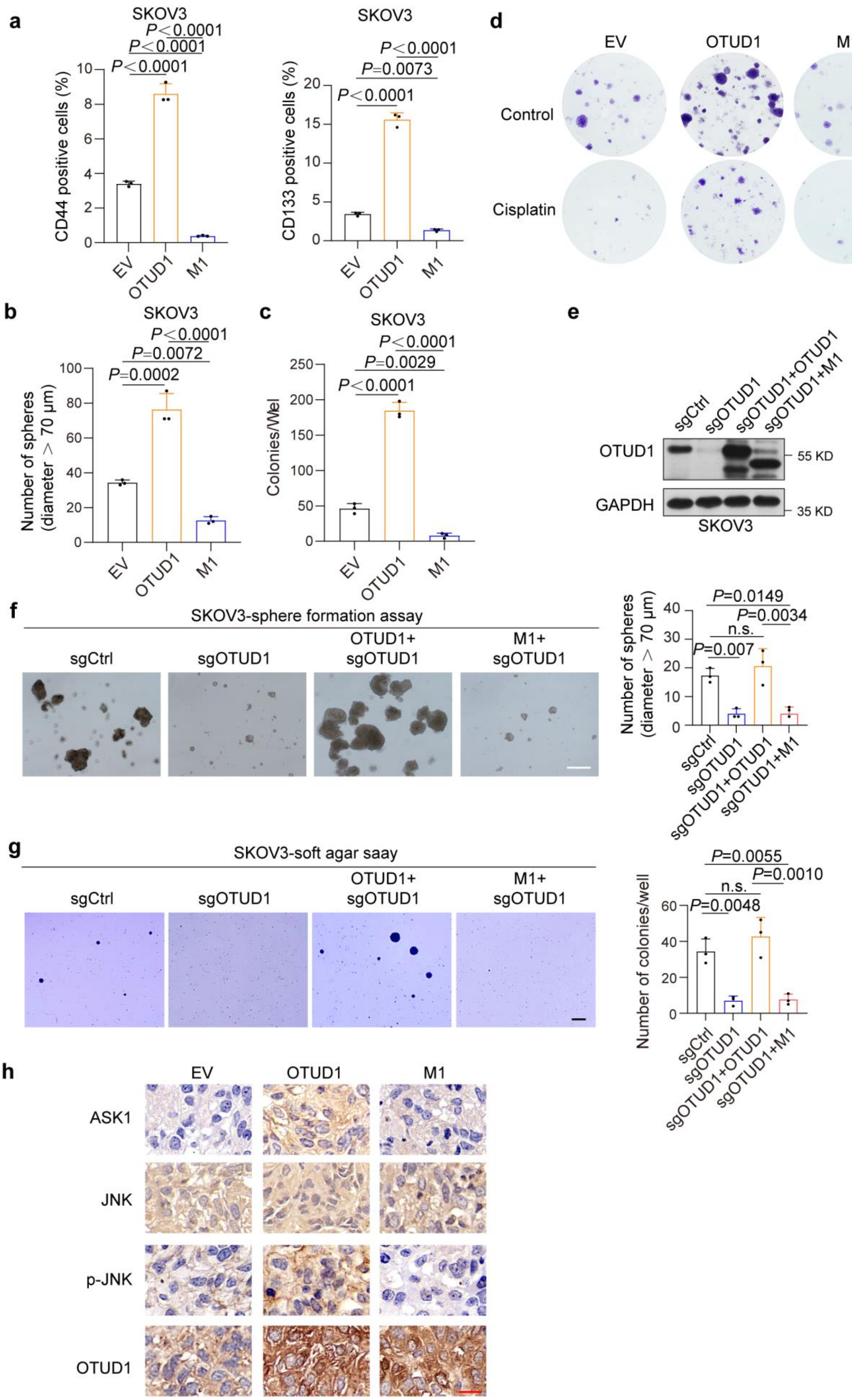
a
SKOV3
P<0.0001
P<0.0001
P<0.0001
CD44 positive cells (%)
EV
OTUD1
M1
SKOV3
P<0.0001
P=0.0073
P<0.0001
CD133 positive cells (%)
d
EV
OTUD1
M1
Control
Cisplatin
b
SKOV3
P<0.0001
P=0.0072
P=0.0002
Number of spheres (diameter > 70 μm)
c
SKOV3
P<0.0001
P=0.0029
P<0.0001
Colonies/Wel
e
sgCtrl
sgOTUD1
sgOTUD1+OTUD1
sgOTUD1+M1
OTUD1
55 KD
GAPDH
35 KD
SKOV3
f
SKOV3-sphere formation assay
sgCtrl
sgOTUD1
OTUD1+ sgOTUD1
M1+ sgOTUD1
P=0.0149
P=0.0034
n.s.
P=0.007
Number of spheres (diameter > 70 μm)
g
SKOV3-soft agar saay
P=0.0055
P=0.0010
n.s.
P=0.0048
Number of colonies/well
h
EV
OTUD1
M1
ASK1
JNK
p-JNK
OTUD1

125

Supplementary Figure 5. (Related to Figure 5) N-terminal disordered region of OTUD1 is essential for its effect on tumorigenesis.

a The percentage of $CD44^+$ or $CD133^+$ CSCs cells in SKOV3.cells expressing OTUD1-WT or Δ105-164 mutant (*n* = 3).

b The counting result of spheres numbers of SKOV3 cells expressing OTUD1-WT or Δ105-164 mutant (*n* = 3).

c The counting result of colony numbers of SKOV3 cells expressing OTUD1-WT or Δ105-164 mutant (*n* = 3).

d The colony formation ability of $OTUD1^{WT}$, $OTUD1^{\Delta 105\text{-}164}$ cells in SKOV3 treated with cisplatin (2 μg/mL) for 14 d.

e $OTUD1^{WT}$ or $OTUD1^{\Delta 105\text{-}164}$ re-expression in SKOV3 OTUD1 deleted cell.was validated by IB assays.

f Representative images of floating spheres formation assay with OTUD1-WT or OTUD1-Δ105-164 (M1) re-expression in SKOV3 KO cells. The spheres numbers were shown in the right panel (*n* = 3). Scale bar, 50 μm.

g Representative images of soft agar assay in OTUD1-WT or OTUD1-Δ105-164 (M1) re-expression in SKOV3 KO cells. The colony numbers to reflect the anchor-independence growth ability were shown in right panel (*n* = 3). Scale bar, 400 μm.

h The IHC analysis of xenograft tumor showing the effect of OTUD1 WT or mutant on ASK1 protein level and JNK phosphorylation. Scale bar, 20 μm.

P values are calculated using one-way ANOVA (**b, c**). Representative of n = 3 independent experiments (**d, e**). Source data are provided as a Source Data file.

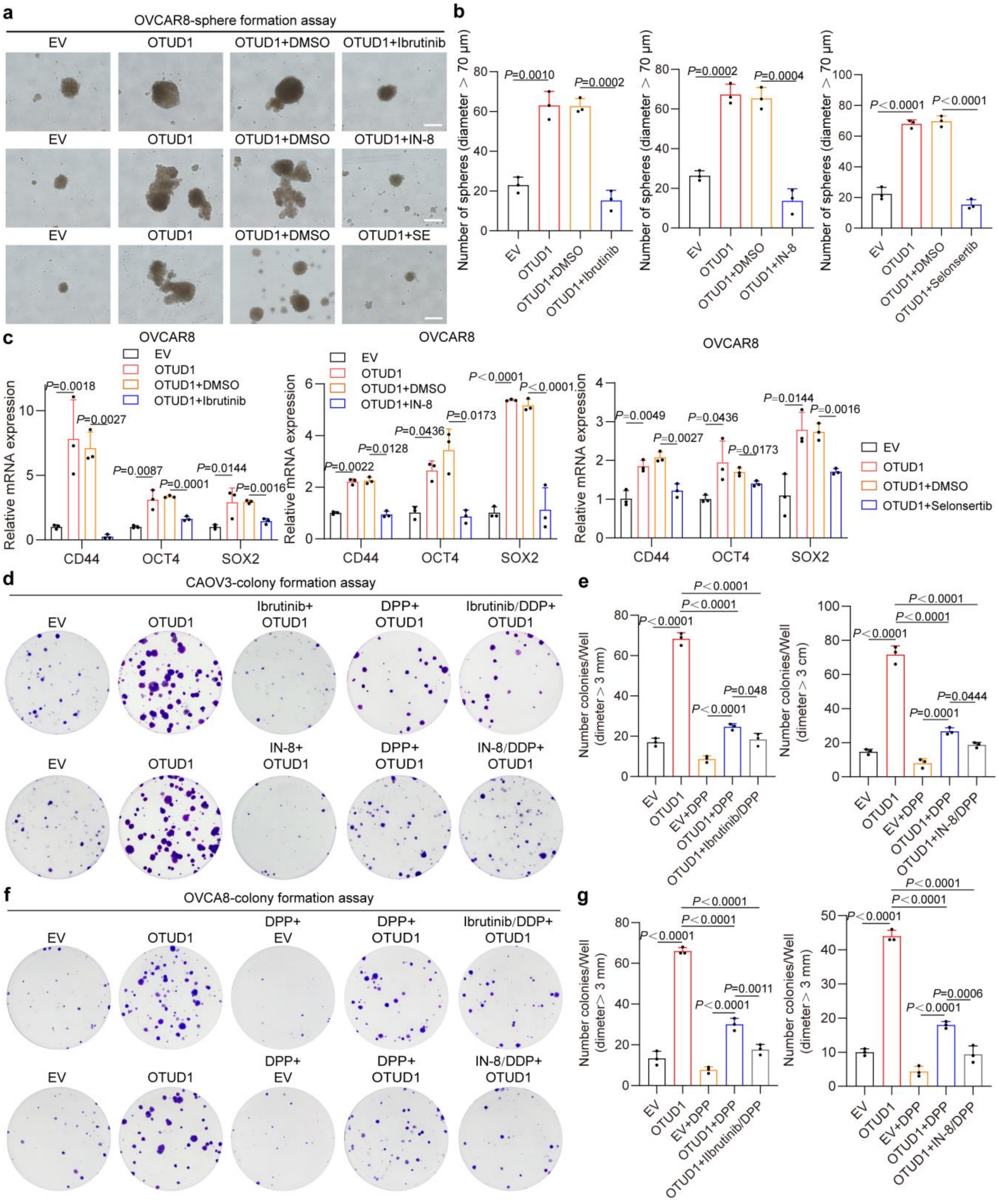

Supplementary Figure 6. (Related to Figure 6) ASK1/JNK inhibitors comprise the stimulatory effect of OTUD1 on OCSC stemness.

a-b Representative images of the effects of OTUD1 overexpressing on sphere-forming in

OVCAR8 cells with or without IN-8 (2 μg/mL), selonsertib (SE, 1μM) or ibrutinib (2.5 μg/mL) respectively (**a**). Scale bar, 50 μm. Spheres counting results showing the effect of three inhibitors in (**a**) on floating sphere-forming ability (**b**). *n* = 3.

c The qPCR assay was used to confirm the expression of stemness-associated genes (ie, *CD44, OCT4* and *SOX2*) in OVCAR8 cells expressing OTUD1 with or without IN-8 (2 μg/mL), selonsertib (SE, 1μM) or ibrutinib (2.5 μg/mL) treatment, respectivel (*n* = 3).

d-e The chemotherapy resistance of CAOV3 cells expressing OTUD1 with or without IN-8 (2 μg/mL), Ibrutinib (2.5 μg/mL), or/and DPP (1 μg/mL) were measured by colony formation assay (**d**). Counting result showing the number of colonies of CAOV3 cell in indicated treatment (**e**). *n* = 3. IN-8, JNK-IN-8. DPP, Cisplatin.

f-g The chemotherapy sensibility of OVCAR8 cells expressing OTUD1 with or without IN-8 (2 μg/mL), Ibrutinib (2.5 μg/mL), or/and DPP (1 μg/mL) were measured by colony formation assay (**f**). Counting result showing the number of colonies of OVCAR8 cell in indicated treatment (**g**). n = 3. IN-8, JNK-IN-8. DPP, Cisplatin.

P values are calculated using two-tailed unpaired Student's t test (**b, c**) and one-way ANOVA (**e, g**). Representative of n = 3 independent experiments (**a, d, f**). Source data are provided as a Source Data file.

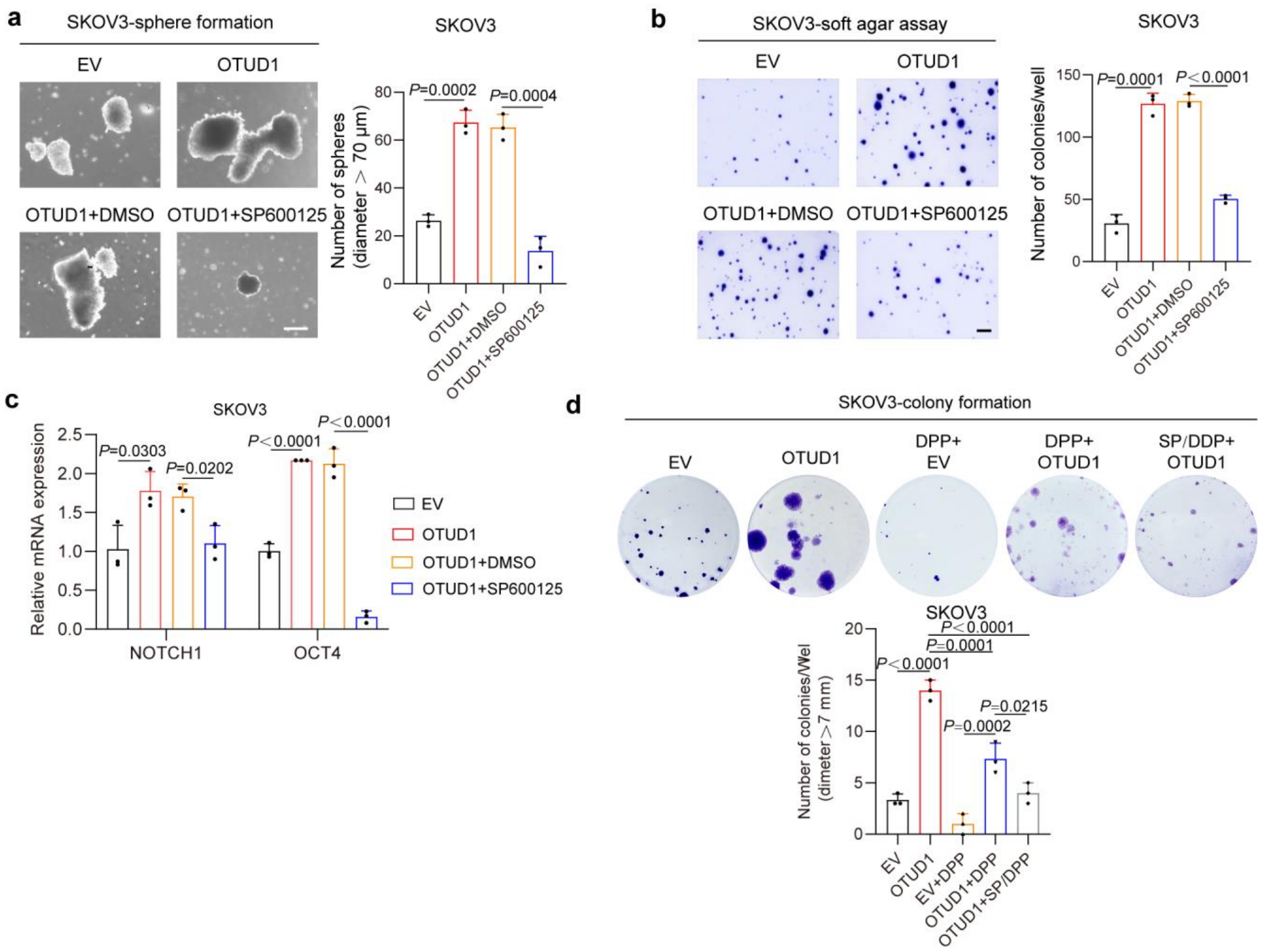

Supplementary Figure 7. (Related to Figure 6) JNK inhibitors comprise the stimulatory effect of OTUD1 on OCSC stemness.

a Representative images of the effects of OTUD1 overexpressing on sphere-forming in SKOV3 cells with or without SP600125 (2 μg/mL) (left panel). Scale bar, 50 μm. Spheres counting results showing the effect of three inhibitors in (**a**) on floating sphere-forming ability (right panel). *n* = 3.

b Representative images of the effects of OTUD1 overexpressing on anchorage-independent growth in SKOV3 cells with or without SP600125 (2 μg/mL) (left panel). Scale bar, 400 μm. Spheres counting results showing the effect of three inhibitors in (**b**) on floating anchorage-independent growth ability (right panel). *n* = 3.

c The qPCR assay was used to confirm the expression of stemness-associated genes (*NOTCH1*, *OCT4*) in SKOV3 cells expressing OTUD1 with or without SP600125 (2 μg/mL). *n* = 3.

d The chemotherapy sensibility of SKOV3 cells expressing OTUD1 with or without SP600125 (2 μg/mL) were measured by colony formation assay. Counting result showing the number of colonies of SKOV3 cell in indicated treatment. *n* = 3.

P values are calculated using two-tailed unpaired Student's t test (**a, b, c**) and one-way ANOVA (**d**). Representative of n = 3 independent experiments (**a-d**). Source data are provided as a Source Data file.

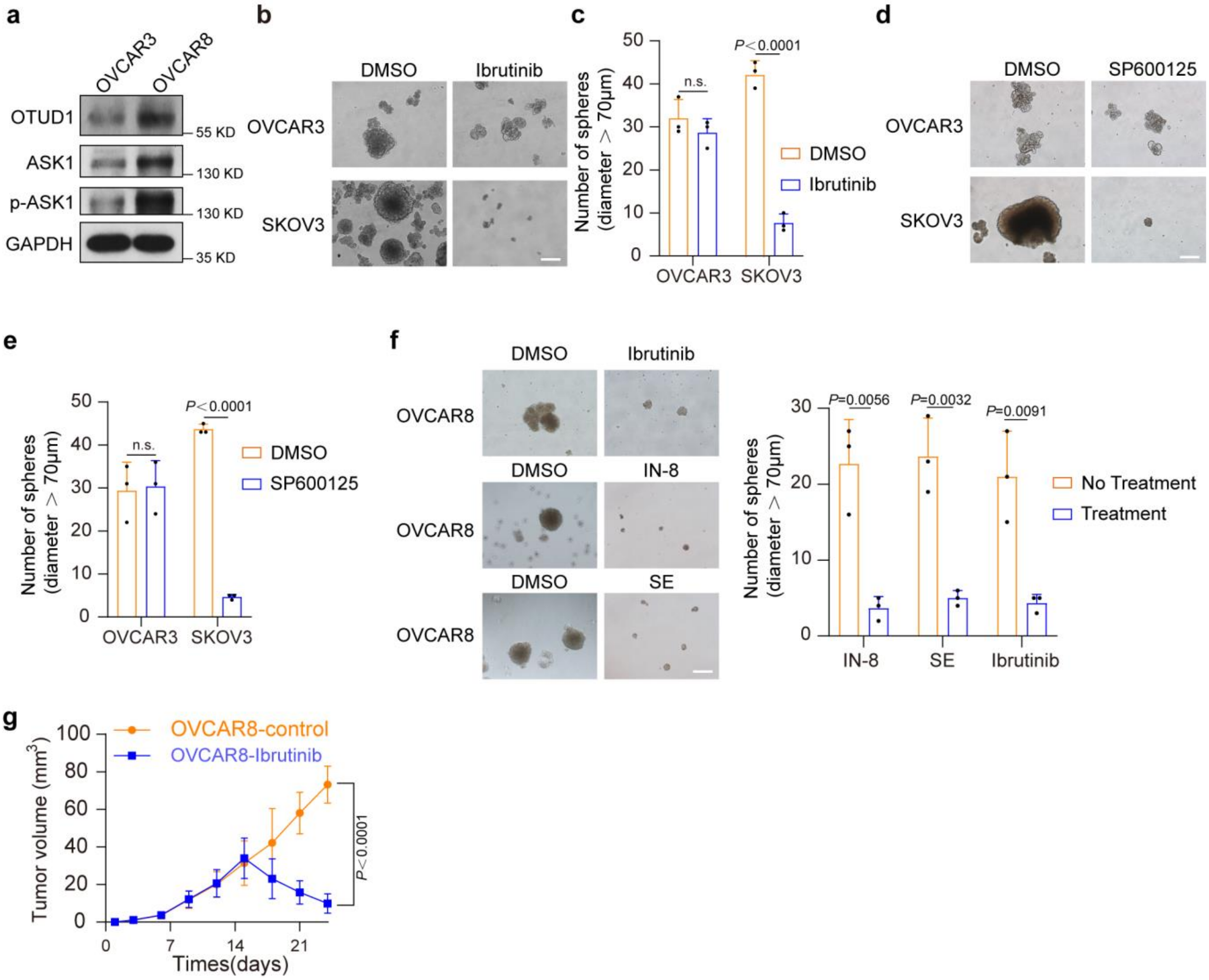

Supplementary Figure 8. (Related to Figure 7) ASK1/JNK preferentially inhibit tumorigenicity of OTUD1^{high} ovarian cancer cells.

a IB analysis of ASK1/p-ASK1 proteins derived from OVCAR3 and OVCAR8 cells.

b-c Representative images of the floating spheres. Floating sphere formation assay was used to evaluate the effect of ibrutinib on sphere formation in SKOV3 and OVCAR3 cells (**b**). Scale bar, 50 μm. Counting results showing the effect of ibrutinib (2.5 μg/mL) on the number of spheres in SKOV3 and OVCAR3 cells (**c**). *n* = 3.

d-e The sphere formation assay was used to examine the effect of SP600125 (2 μg/mL) on SKOV3 cells (**d**). Scale bar, 50 μm. Counting result showing the effect of SP600125 on

number of floating spheres (**e**). *n* = 3.

f The sphere formation assay was used to examine the effect of IN-8 (2 μg/mL), selonsertib (2.5 μg/mL) or ibrutinib (2.5 μg/mL) on OVCAR8 cells (left panel). Scale bar, 50 μm. Counting result showing the effect of IN-8 on number of floating spheres (right panel). *n* = 3.

g OVACR8 cells were subcutaneously injected into BALB/c nude female mice (n = 6 mice per group) with or without ibrutinib administration (25 mg/kg). After 24 d, the mice were sacrificed, tumors were collected and their volume was measured. Tumor growth curve of indicated xenografts are reflected by tumor volume.

P values are calculated using two-tailed unpaired Student's t test (**c, e, f**) and two-way ANOVA (**g**). Representative of n = 3 independent experiments (**a, b, d, f**). Source data are provided as a Source Data file.

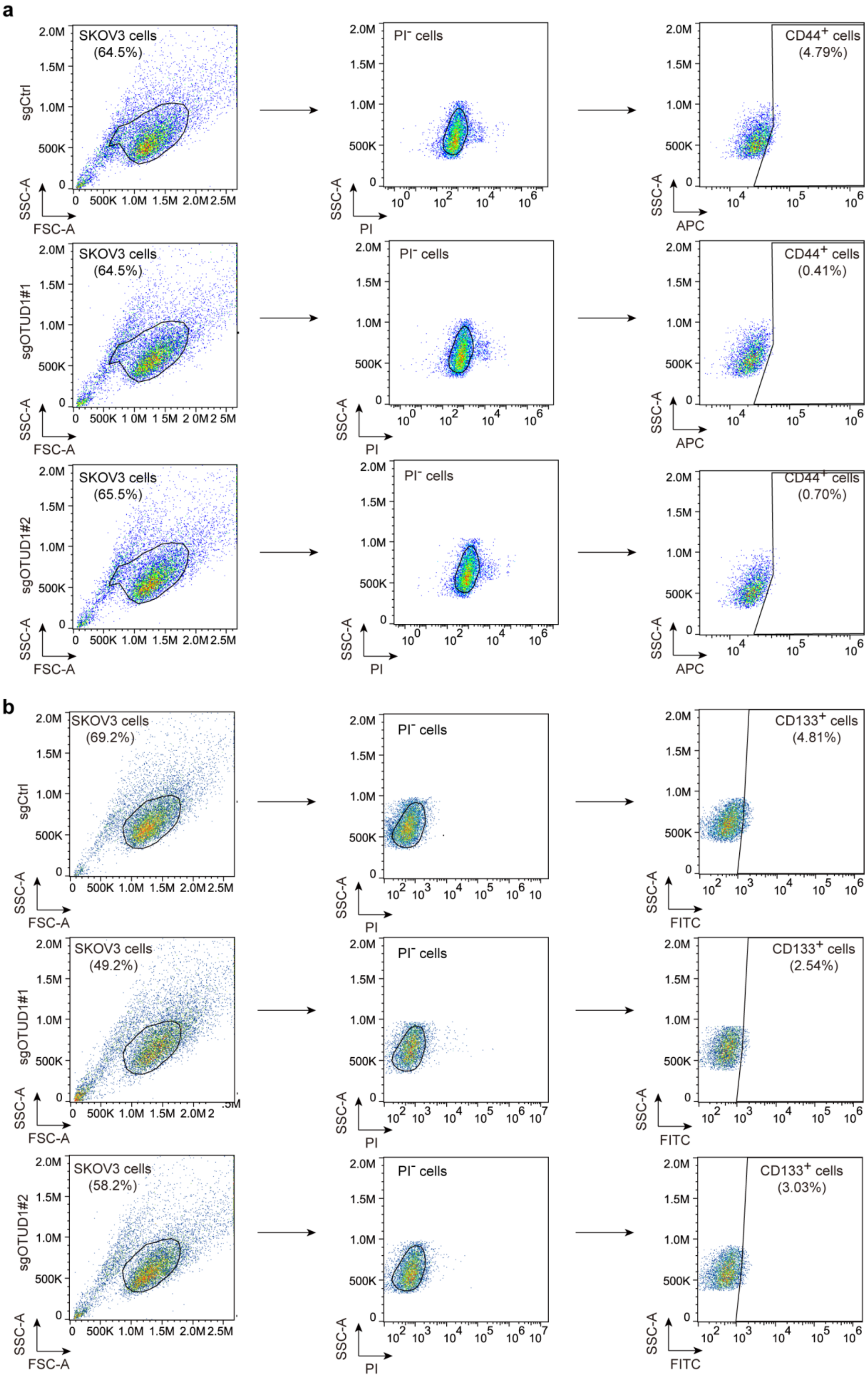
a
sgCtrl
sgOTUD1#1
sgOTUD1#2
SKOV3 cells
(64.5%)
(64.5%)
(65.5%)
PI⁻ cells
CD44⁺ cells
(4.79%)
(0.41%)
(0.70%)
SSC-A
FSC-A
PI
APC
b
SKOV3 cells
(69.2%)
(49.2%)
(58.2%)
CD133⁺ cells
(4.81%)
(2.54%)
(3.03%)
FITC

Supplementary Fig. 9 Gating strategies Gating strategies for flow cytometric analysis in Fig. 1 FSC-A/SSC-A gating was first used to identify cells and removed debris. Live cells were then isolated by negative staining for Propidium Iodide (PI). **a** Gating strategy for analyzing the expression of CD44 in Fig. 1g. **b** Gating strategy for analyzing the expression of CD133 in Fig. 1g.

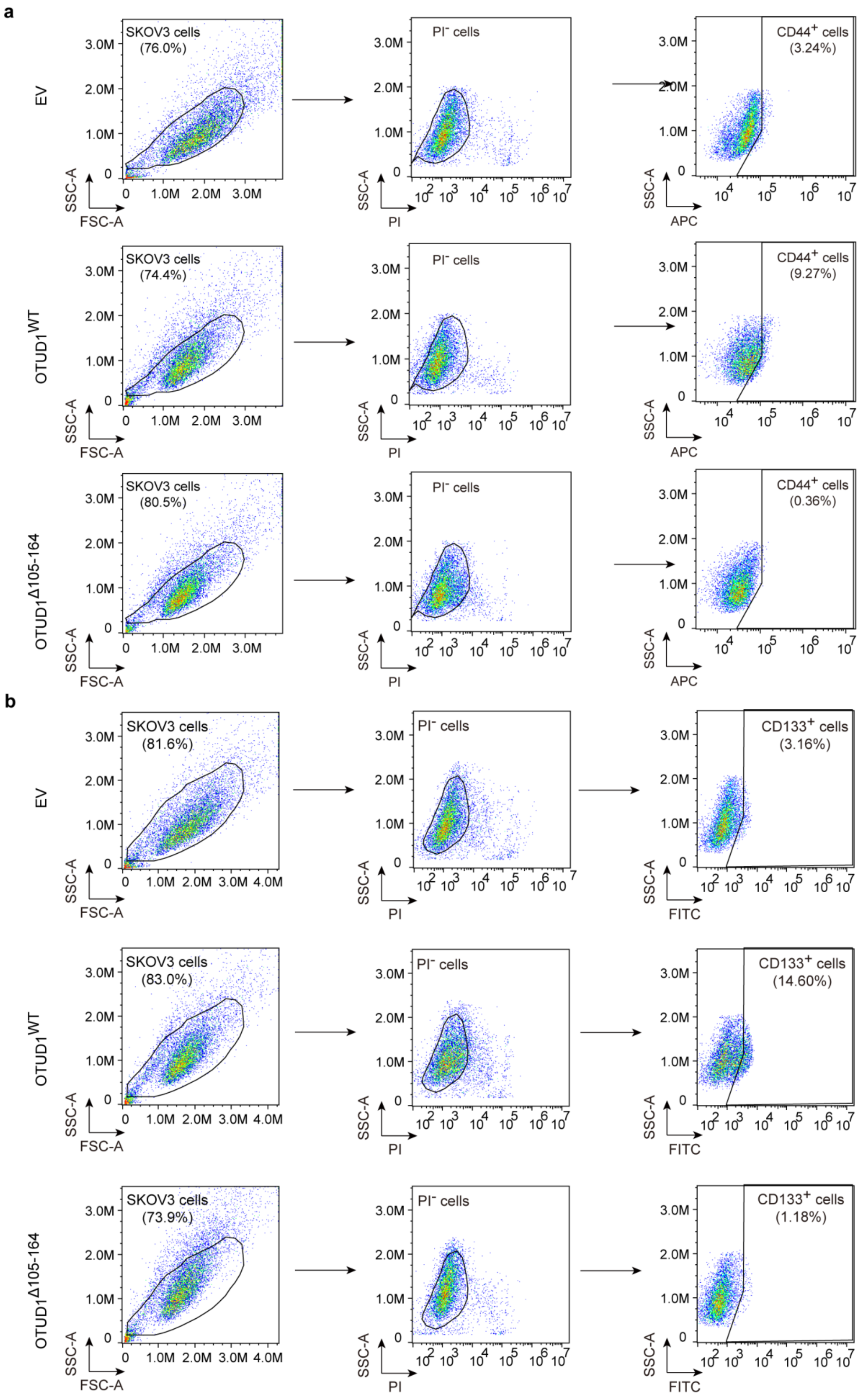
a
EV
SKOV3 cells
(76.0%)
PI⁻ cells
CD44⁺ cells
(3.24%)
OTUD1WT
SKOV3 cells
(74.4%)
PI⁻ cells
CD44⁺ cells
(9.27%)
OTUD1Δ105-164
SKOV3 cells
(80.5%)
PI⁻ cells
CD44⁺ cells
(0.36%)
SSC-A
FSC-A
PI
APC
b
EV
SKOV3 cells
(81.6%)
PI⁻ cells
CD133⁺ cells
(3.16%)
OTUD1WT
SKOV3 cells
(83.0%)
PI⁻ cells
CD133⁺ cells
(14.60%)
OTUD1Δ105-164
SKOV3 cells
(73.9%)
PI⁻ cells
CD133⁺ cells
(1.18%)
SSC-A
FSC-A
PI
FITC

Supplementary Fig. 10 Gating strategies Gating strategies for flow cytometric analysis in Fig. 5. FSC-A/SSC-A gating was first used to identify cells and removed debris. Live cells were then isolated by negative staining for Propidium Iodide (PI). **a** Gating strategy for analyzing the expression of CD44 in Fig. 5d. **b** Gating strategy for analyzing the expression of CD133 in Fig. 5d.

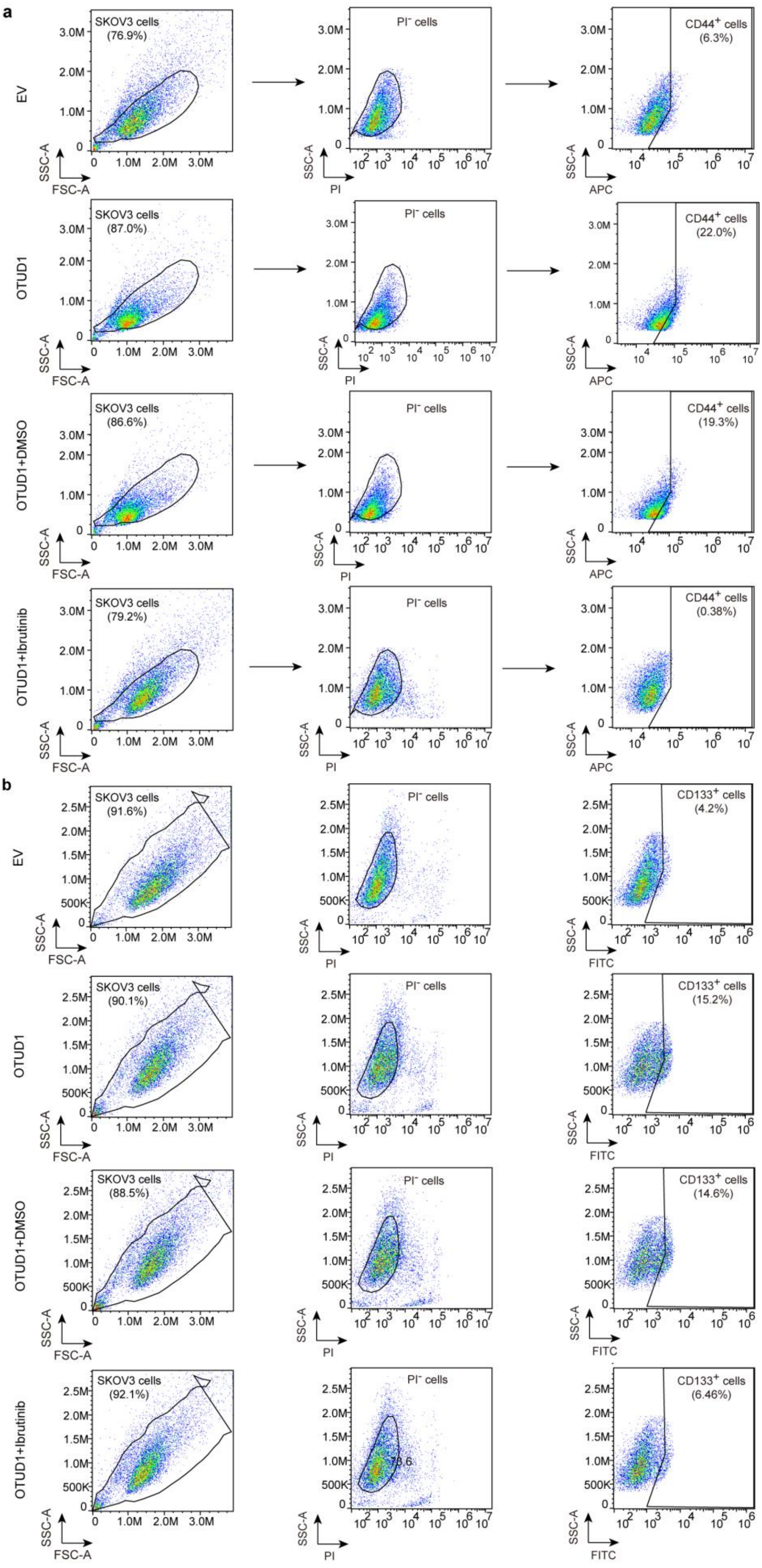
a
EV
OTUD1
OTUD1+DMSO
OTUD1+Ibrutinib
SKOV3 cells (76.9%)
SKOV3 cells (87.0%)
SKOV3 cells (86.6%)
SKOV3 cells (79.2%)
PI⁻ cells
CD44⁺ cells (6.3%)
CD44⁺ cells (22.0%)
CD44⁺ cells (19.3%)
CD44⁺ cells (0.38%)
SSC-A
FSC-A
PI
APC
b
EV
OTUD1
OTUD1+DMSO
OTUD1+Ibrutinib
SKOV3 cells (91.6%)
SKOV3 cells (90.1%)
SKOV3 cells (88.5%)
SKOV3 cells (92.1%)
PI⁻ cells
CD133⁺ cells (4.2%)
CD133⁺ cells (15.2%)
CD133⁺ cells (14.6%)
CD133⁺ cells (6.46%)
SSC-A
FSC-A
PI
FITC

Supplementary Figure 10 Gating strategies Gating strategies for flow cytometric analysis in Figure 6. FSC-A/SSC-A gating was first used to identify cells and removed debris. Live cells were then isolated by negative staining for Propidium Iodide (PI). **a** Gating strategy for analyzing the expression of CD44 in Fig. 6f. **b** Gating strategy for analyzing the expression of CD133 in Fig. 6f.

Supplementary Table 1. Sequences for sgRNA and shRNA.

Name	Primer of sequence (5'-3')
sgOTUD1#1	Forward: CACCGACCGGTCTCGGGCTCGGGCG
	Reverse: AAACCGCCCGAGCCCGAGACCGGTC
sgOTUD1#2	Forward: CACCGCGGTGAGTGCCAGCCCGCCG
	Reverse: AAACCGGCGGGCTGGCACTCACCGC

Supplementary Table 2. Primer sequences for qPCR

Gene		Primer sequence
OTC4	forward	ATGTGGTCCGAGTGTGGTTC
	reverse	GAGACAGGGGGAAAGGCTTC
NANOG	forward	CTCATCAATGCCTGCAGTTTTTCA
	reverse	CTCCTCAGGGCCCTTGTCAGC
NOTCH1	forward	GGAAGTTGAACGAGCATAGTCC
	reverse	GCATGATGCCTACATTTCAAGA
CD133	forward	GGGTGTATCCAAAACCCGGA
	reverse	ACACTGAAAGTTACATCCACAGAA
CD44	forward	GCAGGTATGGGGTTCATAGAAGG
	reverse	GGTGTTGGATGTGAGGATGT
SOX2	forward	GGAGGGGGTGCAAAAGAGGAGAG
	reverse	TCCCCCAAAAAAGAAGTCCAGG
IL6	forward	TTCGGTCCAGTTGCCTTCTC
	reverse	CAGCTCTGGCTTGTTCCTCA
FOS	forward	AGGAGAATCCGAAGGGAAAG
	reverse	CTTCTCCTCCAGCAGGTTGG
ATF2	forward	GGTAGTCTGATTGGCTTAACTCGTA
	reverse	GGCCTGTTAGAGGATGGTGC
GAPDH	forward	TGCACCACCAACTGCTTAGC
	reverse	GGCATGGACTGTGGTCATGAG

Supplementary Table 3. The list of antibodies

Name	Dilution	Co,Ltd	Cat No.	Source
OTUD1	1:1000	Atlas Antibodies	HPA038504	Rabbit
OTUD1	1:200	Atlas Antibodies	HPA038503	Rabbit
OTUD1	1:200	Proteintech	29921-1-AP	Rabbit
ASK1 Rabbit pAb	1:1000	ZENBIO	380952	Rabbit
ASK1 Monoclonal antibody	1:1000	Proteintech	67072-1-Ig	Mouse
ASK1 (Phospho-Thr838) antibody	1:1000	Biorbyt	orb335764	Rabbit

ERK antibody	1:1000	Proteintech	11257-1-AP	Rabbit
Phospho-p44/42 MAPK (Erk1/2) (Thr202/Tyr204) Antibody	1:1000	Cell Signaling Technology	9101	Rabbit
P38 antibody	1:1000	Proteintech	14064-1-AP	Rabbit
Phospho-p38 MAPK (Thr180/Tyr182) Antibody	1:1000	Cell Signaling Technology	9211	Rabbit
JNK Monoclonal antibody	1:1000	Proteintech	66210-1-Ig	Mouse
Phospho-SAPK/JNK (Thr183/Tyr185) (81E11)	1:1000	Cell Signaling Technology	#4668	Rabbit
c-Jun (60A8)	1:1000	Cell Signaling Technology	#9165	Rabbit
Phospho-c-Jun (Ser73) (D47G9)	1:1000	Cell Signaling Technology	#3270	Rabbit
HSP70 Polyclonal antibody	1:200	Proteintech	10995-1-AP	Rabbit
P62, SQSTM1 Polyclonal antibody	1:200	Proteintech	18420-1-AP	Rabbit
GAPDH Monoclonal antibody	1:3000	Proteintech	60004-1-Ig	Mouse
polyclonal anti-Flag tag	1:1000	Proteintech	0543-1-AP	Rabbit
monoclonal anti-HA tag	1:1000	Dia-An Biotech	2063	Mouse
monoclonal anti-Myc tag	1:1000	Dia-An Biotech	2097	Mouse
Goat Anti-Rabbit IgG H&L (Alexa Fluor® 488)	1:200	Abcam	ab150077	Goat
Goat anti-Rabbit IgG H&L (Alexa Fluor 555)	1:200	Abcam	ab150078	Goat
Goat Anti-Rabbit IgG H&L (HRP)	1:5000	Biodragon	BF03008	Goat
Goat Anti-Mouse IgG H&L (HRP)	1:5000	Biodragon	BF03001	Goat
APC Mouse Anti-Human CD44(G44-26)	1:50	BD Pharmingen	559942	Mouse
FITC Mouse Anti-Human CD133(W6B3C1)	1:50	BD Pharmingen	567029	Rabbit
