# Supplementary material for: Aggresome formation promotes ASK1/JNK signaling activation and stemness maintenance in ovarian cancer
Source: Nat Commun. 2024 Feb 13;15:1321. doi: 10.1038/s41467-024-45698-x (PMC10864366; doi:10.1038/s41467-024-45698-x)
Supplement: Supplementary file 3 — Description of Additional Supplementary Files [file 41467_2024_45698_MOESM3_ESM.pdf]

Description of Additional Supplementary Files

File Name: Supplementary Data 1
Description: Genes within 200 kb of susceptibility loci (rs11782652, rs1243180, and rs757210) for ovarian cancer.

File Name: Supplementary Data 2
Description: Genes with copy number amplification (frequency > 4%) in the human serous ovarian cancer samples of The Cancer Genome Atlas (TCGA) Database.
