# Supplementary material for: Aggresome formation promotes ASK1/JNK signaling activation and stemness maintenance in ovarian cancer
Source: Nat Commun. 2024 Feb 13;15:1321. doi: 10.1038/s41467-024-45698-x (PMC10864366; doi:10.1038/s41467-024-45698-x)
Supplement: Supplementary file 5 — Reporting Summary [file 41467_2024_45698_MOESM5_ESM.pdf]

nature portfolio

Corresponding author(s): Jie Xiong, Wei Zhang, Feng Li

Last updated by author(s): Jan 26, 2024

Reporting Summary

Nature Portfolio wishes to improve the reproducibility of the work that we publish. This form provides structure for consistency and transparency in reporting. For further information on Nature Portfolio policies, see our Editorial Policies and the Editorial Policy Checklist.

Statistics

For all statistical analyses, confirm that the following items are present in the figure legend, table legend, main text, or Methods section.

n/a	Confirmed	
☐	☒	The exact sample size (*n*) for each experimental group/condition, given as a discrete number and unit of measurement
☐	☒	A statement on whether measurements were taken from distinct samples or whether the same sample was measured repeatedly
☐	☒	The statistical test(s) used AND whether they are one- or two-sided<br>*Only common tests should be described solely by name; describe more complex techniques in the Methods section.*
☒	☐	A description of all covariates tested
☐	☒	A description of any assumptions or corrections, such as tests of normality and adjustment for multiple comparisons
☐	☒	A full description of the statistical parameters including central tendency (e.g. means) or other basic estimates (e.g. regression coefficient) AND variation (e.g. standard deviation) or associated estimates of uncertainty (e.g. confidence intervals)
☐	☒	For null hypothesis testing, the test statistic (e.g. *F*, *t*, *r*) with confidence intervals, effect sizes, degrees of freedom and *P* value noted<br>*Give P values as exact values whenever suitable.*
☒	☐	For Bayesian analysis, information on the choice of priors and Markov chain Monte Carlo settings
☒	☐	For hierarchical and complex designs, identification of the appropriate level for tests and full reporting of outcomes
☐	☒	Estimates of effect sizes (e.g. Cohen's *d*, Pearson's *r*), indicating how they were calculated

Our web collection on statistics for biologists contains articles on many of the points above.

Software and code

Policy information about availability of computer code

Data collection	CFX Connect Real Time PCR Detection System (Bio-Rad, America);<br>Illumina novaseq-PE150 sequencing platform (Novogene Biotech company, China);<br>ioTeK Micro-plate reader (BAmerica);<br>CytoFLEX flow cytometry (China)<br>Leica Application Suite X (Germany);<br>Olympus microscope (America);

Data analysis	For RNA-Seq data, Featurecount software was used to quantitatively analyze the gene level.<br>The EdgeR software was used to analyze the differential expression of gene in each sample.<br>The KOBAS softeware and Heatmapper versatile web (http://heatmapper.ca/) were employed to analyze the enrichment differential expression of gene.<br>mRNA expression-based stemness index (mRNAsi) were analyzed in ASSISTANT for Clinical Bioinformation (https:// www.aclbi.com/static/index.html#/).<br>For Transwell assay, Image J software (America) was used to count cell numbers.<br>For immunohistochemistry assay, Image J software (America) was used to Semi-quantitative analysis. CaseViewer software (Hungary) was used to view the tissue slides.<br>FlowJo v10.8.1 was used to analyze flow cytometry.<br>Statistical analysis was performed using Graph Pad Prism 9 software (America).

For manuscripts utilizing custom algorithms or software that are central to the research but not yet described in published literature, software must be made available to editors and reviewers. We strongly encourage code deposition in a community repository (e.g. GitHub). See the Nature Portfolio guidelines for submitting code & software for further information.

Data

Policy information about availability of data

All manuscripts must include a data availability statement. This statement should provide the following information, where applicable:

- Accession codes, unique identifiers, or web links for publicly available datasets
- A description of any restrictions on data availability
- For clinical datasets or third party data, please ensure that the statement adheres to our policy

Publicly available datasets reported in this paper are The Cancer Genome Atlas (TCGA, https://portal.gdc.cancer.gov), Kaplan-Meier Plotter (KM-plotter, https://kmplot.com/analysis/), AlphaFold Protein Structure Database (https://alphafold.ebi.ac.uk/entry/Q5VV17), Zhang lab Website Guide Design Tools (http://guides.sanjanalab.org/#/). The RNA-seq data iof floating spheres and floating differentiated cells in this study are deposited in the GEO database under accession code GSE232783 (https://www.ncbi.nlm.nih.gov/geo/query/acc.cgi?acc=GSE232783). The RNA-seq data iof SKOV3 cells engineered to express sgNC or sgOTUD1 in this study are deposited in the GEO database under accession code GSE232786 (https://www.ncbi.nlm.nih.gov/geo/query/acc.cgi?acc=GSE232786). The remaining data are available within the Article, Supplementary Information or Source Data file. Source data are provided with this paper.

Research involving human participants, their data, or biological material

Policy information about studies with human participants or human data. See also policy information about sex, gender (identity/presentation), and sexual orientation and race, ethnicity and racism.

Reporting on sex and gender	No sex and gender analysis were performed.
Reporting on race, ethnicity, or other socially relevant groupings	No race, ethnicity, or other socially relevant groupings were performed.
Population characteristics	Tissue chips containing with 4 cases of low grade ovarian serous carcinoma and 54 cases of high grade ovarian serous carcinoma were enrolled in this study.
Recruitment	Tissues chips were obtained from Shanghai Outdo Biotech Company (#HOvaC070PT01). There were no self-selection bias or other bias that may be present and impact results.
Ethics oversight	All procedures involving human samples were approved by the Ethics Committee of Shanghai Outdo Biotech Company (license no. YBM-05-02) and were in accordance with the Declaration of Helsinki.

Note that full information on the approval of the study protocol must also be provided in the manuscript.

Field-specific reporting

Please select the one below that is the best fit for your research. If you are not sure, read the appropriate sections before making your selection.

☒ Life sciences ☐ Behavioural & social sciences ☐ Ecological, evolutionary & environmental sciences

For a reference copy of the document with all sections, see nature.com/documents/nr-reporting-summary-flat.pdf

Life sciences study design

All studies must disclose on these points even when the disclosure is negative.

Sample size	Although we did not use statistical methods to calculate sample size, we used a minimum of 3 biological replicates. For mice experiments, we based numbers on our previously published experiments (PMID: 36640312) and typical sample size for drug studies documented in the literature (PMID:32004442), taking into account the animal welfare. The exact number of all sample sizes is given in the figure legends.
Data exclusions	No data were excluded from analysis.

Replication	All attempts at replication were performed. The number (n) of biological replicates or animals is indicated as an exact number in the figure legends. A minimum of 3 biological replicates were performed.
Randomization	For animal experiments, same-aged mice were randomly divided into different experimental groups. All cell culture experiments were independently performed at least three times with the same outcome.
Blinding	Blinding was not performed in cell and animal experiments. Because the investigator had to know the groups to perform the study. For animal experiments, same-aged mice were randomly divided into different experimental groups. Experimental groups in cell culture experiments were allocated randomly.

Reporting for specific materials, systems and methods

We require information from authors about some types of materials, experimental systems and methods used in many studies. Here, indicate whether each material, system or method listed is relevant to your study. If you are not sure if a list item applies to your research, read the appropriate section before selecting a response.

Materials & experimental systems

n/a	Involved in the study	
☐	☒	Antibodies
☐	☒	Eukaryotic cell lines
☒	☐	Palaeontology and archaeology
☐	☒	Animals and other organisms
☒	☐	Clinical data
☒	☐	Dual use research of concern
☒	☐	Plants

Methods

n/a	Involved in the study	
☒	☐	ChIP-seq
☐	☒	Flow cytometry
☒	☐	MRI-based neuroimaging

Antibodies

Antibodies used	Anti-OTUD1, Atlas Antibodies, HPA038504 (1:1000 dilution).<br>Anti-OTUD1, Atlas Antibodies, HPA038503 (1:200 dilution).<br>Anti-OTUD1, Proteintech, 29921-1-AP (1:200 dilution).<br>Anti-ERK, Proteintech, 11257-1-AP(1:1000 dilution).<br>Anti-ERK(phospho-p44/42 MAPK (Erk1/2) Thr202/Tyr204), Cell Signaling Technology, #9101 (1:1000 dilution).<br>Anti-p38, Proteintech, 114064-1-AP(1:1000 dilution).<br>Anti-p38(phospho-Thr180/Tyr182),Cell Signaling Technology, #9211 (1:1000 dilution).<br>Anti-ASK1, ZENBIO, 380952 (1:1000 dilution).<br>Anti-ASK1, Proteintech, 67072-1-Ig (1:1000 dilution).<br>Anti-ASK1 (Phospho-Thr838), Biorbyt orb335764 (1:1000 dilution).<br>Anti-JNK, Proteintech, 66210-1-Ig (1:1000 dilution).<br>Anti-SAPK/JNK (Phospho-Thr183/Tyr185) (81E11), Cell Signaling Technology, #4668 (1:1000 dilution).<br>Anti-c-Jun (60A8), Cell Signaling Technology, #9165 (1:1000 dilution).<br>Anti- c-Jun (Phospho-Ser73) (D47G9), Cell Signaling Technology, #3270 (1:1000 dilution).<br>Anti-HSP70, Proteintech, 10995-1-AP (1:200 dilution).<br>Anti-P62 SQSTM1, Proteintech, 18420-1-AP (1:200 dilution).<br>Anti-GAPDH, Proteintech, 60004-1-Ig (1:3000 dilution).<br>Anti-Flag tag, Proteintech, 20543-1-AP (1:1000 dilution).<br>Anti-HA tag, DiaAn Biotech, 2063 (1:1000 dilution).<br>Anti-Myc tag, DiaAn Biotech, 2097 (1:1000 dilution).<br>Goat anti-Rabbit IgG H&L (Alexa Fluor® 488), Abcam, ab150077 (1:200 dilution).<br>Goat anti-Rabbit IgG H&L (Alexa Fluor 555), Abcam, ab150078 (1:200 dilution).<br>Goat anti- Rabbit IgG H&L (HRP), Biodragon, BF03008 (1:1000 dilution).<br>Goat anti-Mouse IgG H&L (HRP), Biodragon, BF03001 (1:1000 dilution).<br>APC Mouse Anti-Human CD44(G44-26), BD Pharmingen, 559942 (1:50 dilution).<br>FITC Mouse Anti-Human CD133(W6B3C1), BD Pharmingen, 567029 (1:50 dilution).
Validation	All antibodies have been validated following the information provided by the manufacturer:<br>Anti-OTUD1, Atlas Antibodies, HPA038504 (https://www.atlasantibodies.com/products/antigens/control-antigens/prest-antigen/otud1-antigen-aprest80327/?q=&t=).<br>Anti-OTUD1, Atlas Antibodies, HPA038503 (https://www.atlasantibodies.com/products/antibodies/primary-antibodies/triple-a-polyclonals/otud1-antibody-hpa038503/?q=HPA038503&t=e6rTf8Rqf1Nlyb4zPoBU9w==).<br>Anti-OTUD1, Proteintech, 29921-1-AP (https://www.ptgcn.com/products/OTUD1-Antibody-29921-1-AP.htm).<br>Anti-ERK, Proteintech, 11257-1-AP(https://www.ptgcn.com/products/ERK1-Antibody-11257-1-AP.htm).<br>Anti-ERK(hospho-Thr202/Tyr204), Cell Signaling Technology, #9101 (https://www.cellsignal.com/products/primary-antibodies/phospho-p44-42-mapk-erk1-2-thr202-tyr204-antibody/9101).<br>Anti-p38,Proteintech, 114064-1-AP(https://www.ptgcn.com/products/p38-Antibody-14064-1-AP.htm).

Anti-p38(hospho-Thr180/Tyr182),Cell Signaling Technology, #9211 (https://www.cellsignal.com/products/primary-antibodies/p38-mapk-antibody/9212).
Anti-ASK1, ZENBIO, 380952 (http://www.zen-bio.cn/prod_view.aspx?IsActiveTarget=True&TypeId=180&Id=537308&FId=t3:180:3).
Anti-ASK1, Proteintech, 67072-1-Ig (https://www.ptgcn.com/products/ASK1-Antibody-67072-1-Ig.htm).
Anti-ASK1 (Phospho-Thr838), Biorbyt, orb335764 (https://biorbyt.com.cn/ask1-phospho-thr838-antibody-orb335764.html).
Anti-JNK, Proteintech, 66210-1-Ig (https://ptgcn.com/products/JNK-Antibody-66210-1-Ig.htm).
Anti-SAPK/JNK (Phospho-Thr183/Tyr185) (81E11), Cell Signaling Technology, #4668 (https://www.cellsignal.cn/products/primary-antibodies/phospho-sapk-jnk-thr183-tyr185-81e11-rabbit-mab/4668?site-search-type=Products&N=4294956287&Ntt=%234668&fromPage=plp&_requestid=1882359).
Anti-c-Jun (60A8), Cell Signaling Technology, #9165 (https://www.cellsignal.cn/products/primary-antibodies/c-jun-60a8-rabbit-mab/9165?site-search-type=Products&N=4294956287&Ntt=%239165&fromPage=plp&_requestid=1882428).
Anti- c-Jun (Phospho-Ser73) (D47G9), Cell Signaling Technology, #3270 (https://www.cellsignal.cn/products/primary-antibodies/phospho-c-jun-ser73-d47g9-xp-rabbit-mab/3270?site-search-type=Products&N=4294956287&Ntt=%233270&fromPage=plp&_requestid=1882489).
Anti-HSP70, Proteintech, 10995-1-AP (https://www.ptgcn.com/products/HSPA1A-Antibody-10995-1-AP.htm).
Anti-P62 SQSTM1, Proteintech, 18420-1-AP (https://www.ptgcn.com/products/SQSTM1-Antibody-18420-1-AP.htm).
Anti-GAPDH, Proteintech, 60004-1-Ig (https://www.ptgcn.com/products/GAPDH-Antibody-60004-1-Ig.htm).
Anti-Flag tag, Proteintech, 0543-1-AP (https://www.ptglab.com/products/Flag-Tag-Antibody-20543-1-AP.htm).
Anti-HA tag, DiaAn Biotech, 2063 (https://www.dia-an.cn/index/product.html?pro_id=4330&aid=48,49,53#view_zjy).
Anti-Myc tag, DiaAn Biotech, 2097 (https://www.dia-an.cn/index/product.html?pro_id=4338&aid=48,49,53#view_zjy).
Secondary anti-Rabbit IgG H&L (Alexa Fluor® 488), Abcam, ab150077 (https://www.abcam.cn/products/secondary-antibodies/goat-rabbit-igg-hl-alexa-fluor-488-ab150077.html).
Secondary anti-Rabbit IgG H&L (Alexa Fluor 555), Abcam, ab150078 (https://www.abcam.cn/products/secondary-antibodies/goat-rabbit-igg-hl-alexa-fluor-555-ab150078.html).
Secondary anti- Rabbit IgG H&L (HRP), Biodragon, BF03008 (https://www.biodragon.cn/hrpbjek/74422.html).
Secondary anti-Mouse IgG H&L (HRP), Biodragon, BF03001 (https://www.biodragon.cn/hrpbjek/74425.html).
APC Mouse Anti-Human CD44(G44-26), BD Pharmingen, 559942 (https://www.bdbiosciences.com/en-us/products/reagents/flow-cytometry-reagents/research-reagents/single-color-antibodies-ruo/apc-mouse-anti-human-cd44.559942).
FITC Mouse Anti-Human CD133(W6B3C1), BD Pharmingen, 567029 (https://www.bdbiosciences.com/en-us/products/reagents/flow-cytometry-reagents/research-reagents/single-color-antibodies-ruo/fitc-mouse-anti-human-cd133.567029).

Eukaryotic cell lines

Policy information about cell lines and Sex and Gender in Research

Cell line source(s)	293T (SCSP-502), SKOV3 (TCHu185), CAOV3 (SCSP-570), OVCAR8, and OVCAR3 (TCHu228) cell lines originated from the National Collection of Authenticated Cell Cultures (Shanghai, China). OVCAR8 cell line was a gift from Professor Chaoyang Sun (Tongji Hospital, Tongji Medical College, Huazhong University of Science and Technology).
Authentication	All cell lines were authenticated in our lab by morphological examination using microscope and were authenticated genetically.
Mycoplasma contamination	All cell lines were tested negative for mycoplasma contamination by Mycoplasma Rapid Test kit (Quick Cell, AC16L061) to ensure that they are mycoplasma free.
Commonly misidentified lines (See ICLAC register)	There is no ICLAC line used in this study.

Animals and other research organisms

Policy information about studies involving animals; ARRIVE guidelines recommended for reporting animal research, and Sex and Gender in Research

Laboratory animals	5-week-old female BALB/c nude mice were from from Wuhan Wanqianjiaxing Biotechnology Co., Ltd (China, Wuhan).All animals were maintained in a specific pathogen-free environment and housed with no more than five animals per cage under controlled light (12-hour light and 12-hour dark cycle), temperature (24 ± 2oC) and humidity (50% ± 10%) conditions, and provided with ad libitum access to food and water throughout all experiments.
Wild animals	This study did not involve wild animals.
Reporting on sex	Female BALB/c nude mice aged 6 weeks were used for CDX experiments.
Field-collected samples	This study did not involve samples collected from the field.
Ethics oversight	All the animal used in this study were evaluated and approved by the Experimental Animal Welfare Ethics Committee, Zhongnan Hospital of Wuhan University (license no. ZN2022255).

Note that full information on the approval of the study protocol must also be provided in the manuscript.

Plants

Seed stocks

Report on the source of all seed stocks or other plant material used. If applicable, state the seed stock centre and catalogue number. If plant specimens were collected from the field, describe the collection location, date and sampling procedures.

Novel plant genotypes

Describe the methods by which all novel plant genotypes were produced. This includes those generated by transgenic approaches, gene editing, chemical/radiation-based mutagenesis and hybridization. For transgenic lines, describe the transformation method, the number of independent lines analyzed and the generation upon which experiments were performed. For gene-edited lines, describe the editor used, the endogenous sequence targeted for editing, the targeting guide RNA sequence (if applicable) and how the editor was applied.

Authentication

Describe any authentication procedures for each seed stock used or novel genotype generated. Describe any experiments used to assess the effect of a mutation and, where applicable, how potential secondary effects (e.g. second site T-DNA insertions, mosiacism, off-target gene editing) were examined.

Flow Cytometry

Plots

Confirm that:

☒ The axis labels state the marker and fluorochrome used (e.g. CD4-FITC).

☒ The axis scales are clearly visible. Include numbers along axes only for bottom left plot of group (a 'group' is an analysis of identical markers).

☒ All plots are contour plots with outliers or pseudocolor plots.

☒ A numerical value for number of cells or percentage (with statistics) is provided.

Methodology

Sample preparation	Diefferent gene-type SKOV3 cell lines (EV, OTUD1-WT, OTUD1-M1, sgRNA-Ctrl, sgOTUD1) were cultured in DMEM containing 10% fetal calf serum (FCS, Gibco) and antibiotics at 37 °C in a 95% air/5% CO2 humidified atmosphere.<br>Diefferent gene-type OVCAR3 cell lines (sgRNA-Ctrl, sgOTUD1) were cultured in 1640 containing 20% fetal calf serum (FCS, Gibco) and antibiotics at 37 °C in a 95% air/5% CO2 humidified atmosphere.<br>Diefferent gene-type OVCAR8 and CAOV3 cell lines (sgRNA-Ctrl, sgOTUD1) were cultured in DMEM containing 10% fetal calf serum (FCS, Gibco) and antibiotics at 37 °C in a 95% air/5% CO2 humidified atmosphere.<br>Diefferent gene-type HEK293T cell lines (EV, OTUD1-WT, OTUD1-M1) were cultured in DMEM containing 10% fetal calf serum (FCS, Gibco) and antibiotics at 37 °C in a 95% air/5% CO2 humidified atmosphere.
Instrument	For Flow analysis, we used CytoFLEX instrument.
Software	CytoFLEX Flow cytometer was used for data collection. FlowJo v10.8.1 was used for analysed.
Cell population abundance	The cells were detached with pancreatic ferment (Sigma, USA), blocked with PBS containing 2% BSA, washed with PBS containing 0.1% BSA and incubated with the respective antibody conjugated with fluorescein isothiocyanate (FITC) or allophycocyanin (APC) including CD133 and CD44 (BD, PharMingen).
Gating strategy	FSC-A/SSC-A gating was first used to identify cells and removed debris. Live cells were then isolated by negative staining for Propidium Iodide (PI). Expression of indicated proteins were checked on these populations as indicated in the figures and figure legends.

☒ Tick this box to confirm that a figure exemplifying the gating strategy is provided in the Supplementary Information.
