# Supplementary material for: Aggresome formation promotes ASK1/JNK signaling activation and stemness maintenance in ovarian cancer
Source: Nat Commun. 2024 Feb 13;15:1321. doi: 10.1038/s41467-024-45698-x (PMC10864366; doi:10.1038/s41467-024-45698-x)
Supplement: Supplementary file 2 — Peer Review File [file 41467_2024_45698_MOESM2_ESM.pdf]

Peer Review File

Aggresome formation promotes ASK1/JNK signaling activation and stemness maintenance in ovarian cancer

Open Access This file is licensed under a Creative Commons Attribution 4.0 International License, which permits use, sharing, adaptation, distribution and reproduction in any medium or format, as long as you give appropriate credit to the original author(s) and the source, provide a link to the Creative Commons license, and indicate if changes were made. In the cases where the authors are anonymous, such as is the case for the reports of anonymous peer reviewers, author attribution should be to 'Anonymous Referee' followed by a clear attribution to the source work. The images or other third party material in this file are included in the article's Creative Commons license, unless indicated otherwise in a credit line to the material. If material is not included in the article's Creative Commons license and your intended use is not permitted by statutory regulation or exceeds the permitted use, you will need to obtain permission directly from the copyright holder. To view a copy of this license, visit http://creativecommons.org/licenses/by/4.0/.

Reviewers' Comments:

Reviewer #1:
Remarks to the Author:
Given their focus is on an ovarian serous histophenotype the inclusion of SKOV3 and A2780 cell lines is questionable. These two lines, albeit used extensively in the past for ovarian cancer research have fallen out of favor as optimal models since it has been shown they are less like high grade ovarian serous cancer (Domcke et al., 2013) then more appropriate lines. Moreover, A2780 cells were originally derived from an ovarian endometrioid subtype and are p53 wild type. The OVCAR3 cell line is considered a line more representative of HGSOC. It is highly recommended the investigators expand their cell line repertoire to include more lines representative of HGSOC histophenotype if investigators want to make the claims related to HGSOC.

It has long been appreciated that the JNK signaling pathway contributes to the maintenance of CSC like cell in several types of solid tumors. There are also published studies demonstrating that inhibition of the JNK signaling can negatively impact self-renewal and tumor initiating capacity of A2780 CSCs (Okada et al. 2005, Seino et al., 2014 and Seino et al 2016 and should be included). However, the upstream mechanism(s) leading to JNK mediated support of the ovarian cancer stem cells was not known. More specifically the link between OTUD1 and aggresome like organelles promoting ASK1 sequestration and stability activating JNK and subsequently supporting CSCs was not appreciated. This has the potential to move the field forward.

In the introduction the authors focus on the epithelium of the ovarian surface epithelium. They point out that it is less differentiated than the epithelium of other anatomical sites. However, it is well accepted now that the bulk of the high grade serous ovarian cancer cases arise in the epithelium of the fallopian tube and not the ovarian surface epithelium. However, there is not reference to this in their manuscript which implies there may be limited background in the field and understanding of the disease itself.

The authors imply that OTUD1 maintains tumor dedifferentiation but also implies it promotes dedifferentiation – this raises the question - does treatment induced enrichment of CSCs result in further dedifferentiation? Specifically, are the authors implying OTUD1 de differentiates CSCs or are they implying it is causing non-CSCs to take on some stem like properties via dedifferentiation?

Examples: Line 93 …….cancer stem cell de-differentiation in ovarian cancer or Line 144 -145 ….by which OTUD1 regulates ovarian cancer stemness and dedifferentiation. Their differeing use of the term dedifferentiation makes it challenging to interpret what they mean exactly.

Also in their early analyses it was not readily clear what parameters were used for determining a de-differentiated cell from a differentiated cell.

Line 122- 123 It is not clear by what the authors mean with regards to the statement ……high grade serous ovarian cancer ………possesses enhanced OCSCs….. Secondarily, are the authors implying that low grade serous ovarian cancer do not use the same mechanisms to maintain their CSCs or do not rely on CSCs?

The IHC images provided for the HGSOC and LGSC are of poor quality and the staining for OTUD1 is not clear enough to discern organelles. In some instances, the staining appears diffuse and in others it appears to be localized to the nuclei. Moreover, if the staining is representative of OTUD1 and CSCs as implied then these images would suggest that the bulk of the tumor cells within HGSOCs are CSCs.

The investigators suggest they used CRISPR to knockout OTUD1 yet their immunoblots of the xenograft tumors suggest it was not completely knocked out. Please explain.

The authors appear to focus their efforts on stem like markers in SKOV3 cells but not the other lines. Given their implied interest in HGSOCs it would be more relevant to demonstrate the loss knockdown of OTUD1 on CSC markers in the OVCAR3 cells as well as for all cells used herein the present study.

Line 80 …… the lack of biomarkers for OCSC-targeted therapy. Please explain given the authors themselves list a number of biomarkers for assessment of OCSC.

Line 161 – Is this protein or mRNA?

Line 174 Figure 3 b shows two cell lines, but authors refer to other ovarian lines…. Which ones?

Why did the authors use HEK293 cells for their experiments? Please justify what they would not perform these experiments in ovarian lines for further proof of concept?

Lines 271 -276The flow data results, colony forming and sphere forming data are not accompanied by any statistical supportive data. If the authors are going to make statements related to changes/differences then this information needs to replicated and p values provided.

The in vivo data is highly informative. It's unfortunate the ibrutinib treatment was only shown to be significant on SKOV3 xenografts and not the ovcar3 xenografts which is more representative of the serous phenotype. This implies their conclusions may be over interpreted. This is especially true given the lack of true preclinical models of high grade ovarian cancer.

Moreover, given the number of published studies on treatment induced enrichment of CSCs in preclinical models high grade serous ovarian cancer the authors should make the effort to better appreciate how this would alter OTUD1, aggresome formation and subsequent ASK1 signaling.

Line 423 – While targeting OTUD1 is of interest there is not enough data provided to support the fact the statement as a therapeutic target for high grade serous ovarian cancer.

Reviewer #2:
Remarks to the Author:
This paper investigates the mechanism underpinning ovarian cancer cell de-differentiation. The data show that ovarian cancer cells exhibiting characteristics of stem cells display increased expression of the deubiquitinase OTUD1 that leads to the formation of aggresome-like organelles. The stabilization of ASK1 following its recruitment to these organelles via direct interaction with OTUD1 activates JNK. ASK1/JNK activation associated with elevated OTUD1 expression contributes to maintaining ovarian cancer stem cells (OCSCs) tumorigenic properties.

Opinion:

The paper contains a large amount of interesting data. However, I have raised a number of main concerns that must be addressed prior to considering the paper for publication in Nature Communications.

1 - In addition to providing evidence that different ovarian cancer cell lines (i.e. OVCAR3 and SKOV3) exhibit different levels of OTUD1 protein (Figure 7A), the authors must demonstrate that the protein level of OTUD1 in floating SKVO3 spheres (i.e. SKOV3 cells seeded in ultra-low-attachment plates) is higher compared with that in differentiated cells (i.e. SKOV3 spheres re-cultured in plates). These data should be included in Figure 1 to confirm that OTUD1 is important for maintaining ovarian cancer stem cell characteristics.

2- Figure 2D: The apparent very high level of ectopic expression of OTUD1 in SKOV3 raises concern about the possibility of artefacts associated with transfection of expression vectors to study protein functions, in particular as a consequence of overwhelming endogenous protein sorting processes. Therefore, the authors must repeat this experiment using an inducible system (e.g. the dox-inducible EGFP-OTUD1 plasmid they used in Figure 3H) that would permit to achieve a level of ectopic OTUD1 expression similar to that observed in spheres (see my comment above). This should be done even if the OTUD1M1 mutant does not appear to form aggresomes (Figure 4F), despite expressing at the same level as the wild type protein (Figure 4D/4H). A similar criticism can be made when interpreting the data obtained based on ASK1 overexpression (Figure 3).

3- Figure 2 Panels A and B: The justification for focusing on JNK signalling is weak for a number of reasons:

a) The role of JNK in CSC remains controversial with some studies suggesting that JNK does not affect CSC activity, while others indicating that JNK contributes to maintaining CSC properties. Hence, the authors must revise their statement that: "MAPK/JNK activation has been frequently implicated in CSC activity regulation in various cancer types" (line 150-151) to reflect these conflicting findings. Also, a reference should be added to support the following statement: "Because the active JNK pathway has been shown to be involved in CSC maintenance by numerous studies,…" (line 262).

b) The list of genes regulated by OTUD1 and presented in panel B [a mixture of markers of CSC identity together with transcription factors and MAPK6 (ERK3), MAP2K1 (MEK1), MAPK3K8)], does not comprise any particularly convincing JNK targets.

c) Line 149: "Given the smaller P value of the MAPK pathway compared with those of the other pathways…" which P value are the authors referring to? Which MAPK pathway? What about JNK? Is the P value the most appropriate to select a specific pathway? I am not convinced.

To address this issue the authors must re-analyze their RNA-seq data to identify known JNK target genes in addition to FOS. These data are critical to confirm the possibility that the level of OTUD1 determine the level of JNK activity. In addition, the legend of Figure 2A should be revised to clarify how the graph was produced and detail of the strategy for knocking out the OTUD1 locus in ovarian cancer cells by CRISPR-Cas9 should be provided. In particular, sequencing of the probes must be provided.

4 - Figure 6 and 7D-G: The interpretation of these data acquired from analysing the effect of SP600125 and ibrutinib is flowed because these are not specific JNK inhibitors. Therefore, the panels where SP600125 and ibrutinib are employed must be moved to supplementary data and interpreted in light of their non-specific effect. Where appropriate, these experiments must be repeated by using the ASK1 specific inhibitor selonsertin.

5- The removal of the data based on the use of SP600125 and ibrutinib will inevitably weaken the demonstration that JNK determines ovarian cancer stem cell phenotypes. Therefore, to firmly establish a link between JNK and the maintenance of stemness, the authors must provide experimental evidence that JNK-mediated c-Jun activation transcriptionally regulates some of the markers of ovarian cancer stem cells listed in Figure 7I.

Other comments

1 - Supplementary Figure 3A: The data presented in the lower panel do not make any sense. The HA antibody does not detect HA-OTUD1(N) in the first input (lane 1). The Flag antibody detects a protein in the anti-Flag immunocomplex from cells that do not express Flag-tagged OTUD1(N). Please clarify.

2 - Supplementary Figure 3F shows that the JNK/c-Jun pathway was inhibited after knocking out

OTUD1. These data should be moved to where the hypothesis that JNK is a downstream target of OTUD1 is first described (Fig. 2D).

3 - Figure 4H: Expression of Δ105-164 OTUD1 mutant appears to decrease ASK1 expression and inhibit ASK1 and JNK phosphorylation, without affecting the phosphorylation of c-Jun. Based on the hypothesis that c-Jun is a substrate of JNK, this observation does not make any sense. Please explain.

4 - Figure 4I is confusing because the panels with antibodies are mislabelled. Also, how is Ha-OTUD1 distinguished from Ha- ASK1(N). I presume that these two proteins exhibit different molecular weight, but this is not clear.

5 - Figure 4J: Similarly, I presume that the Flag panel shows Flag-ASK1 ad not Flag-OTUD1 (WT or mutant). This must be clarified.

6 - Most figure legends must be revised to clarify experimental designs and data presentations.

Reviewer #3:
Remarks to the Author:
This work detailed in this manuscript concerns the identification of biomarkers for OCSC-targeted therapy. Here, Chen et al. performed multi-omics screening to identify OTUD1 as a key factor of the OCSC dedifferentiation and tumorigenesis. A role for OTUD1 expression in maintaining stemness and promoting tumorigenic activity of OCSC is described. OTUD1 overexpression was shown to induce aggresome formation, which is responsible for the stabilization of the key JNK component, ASK1, which leads to the activation of the JNK pathway to potentiate OCSC stemness maintenance and tumorigenicity. Importantly, JNK pathways inhibitors, including the FDA-approved ibrutinib, suppressed the OTUD1-induced OCSC stemness maintenance, tumorigenesis and chemoresistance, highlighting the potential of combining JNK inhibitors and standard chemotherapeutics to treat ovarian cancer with OTUD1 overexpression or OCSC maintaining ability.
Mechanistically, they find that OTUD1 overexpression induced aggresome formation, which sequesters ASK1, resulting in its stabilization and enhanced activity that drives JNK pathway activation, thus maintaining OCSC stemness. The authors demonstrated that mutating the N-terminal intrinsically disordered region (IDR) of OTUD1, shown to be critical for OTUD1-based aggresome formation in the study, decreased ASK1 activation, disrupted OCSC stemness maintenance and reduced the tumorigenicity of xenotransplanted SKOV3 cells. The findings detailed in this manuscript are generally novel. Aggresome formation is a well-recognized hallmark of neurodegenerative diseases but its role in cancer is less well understood. Additionally, proteins sequestered in the aggresome are generally thought to be inactive, leading to the loss of functions of certain proteins. The authors show that aggresome formation can activate a cancer signaling pathway and provide novel insights on the role of aggresome in the pathology of cancer. This work also provides a novel example that proteins sequestered in the aggresome could be functional to illicit downstream cellular effects.
Despite these many novelties, I do have some concerns about the data and some key quantifications are missing, which dampens my enthusiasm about some of the major conclusions made by the authors. Additionally, some sections of the main text are not presented logically and should reworked to make the article easier to follow. The following major revisions need to be carried out prior to consideration for publication:

Major Points:

1. In Fig. 1, Fig. 2 and Supplemental Fig. 1, the sgRNA-mediated knock out of OTUD1 in SKOV1 and A2780 cells reduced stemness, sphere formation and colony formation. The authors need to perform rescue experiments using WT- and M1-OTUD1 re-expression in these contexts to exclude possible off-target effects and requirement of IDR domain. This would make the model proposed in this study

more convincing.

2. In Supplemental Fig. 1D, the decrease of OTUD1 level in differentiated A2780 cells seems marginal, which may compromise the conclusion regarding the roles of OTUD1 in OCSC stemness maintenance. It would be more convincing to show densitometric quantification of the band intensity and the statistical significance of the decrease based on several biological replicates.

3. Fig. 2F, why was the FLAG epitope detected in the FLAG-IP sample purified from cells that are not expressing FLAG-OTUD1? Is it an unspecific band? Also, HA is not detected in the input sample from cells expressing HA-ASK1, which is very confusing. Were the ectopic expression and the IP procedures successful?

4. On line 195-201, the rationale to perform nocodazole treatment to confirm the formation of OTUD1-based aggresome is not logically explained. Microtubule dynamics is known to promote aggresome formation (doi: 10.1385/JMN:29:2:153), but the rationale here emphasized the overwhelmed proteolysis system as the cause of aggresome formation. A rephased rationale and a more appropriate citation would be preferred.

5. In Fig. 4I, FLAG-IP was performed using HEK293T cells expressing FLAG-ASK1 (N), HA-ASK1 (N) and HA-OTUD1. Since both OTUD1 and ASK1 (N) were tagged with HA, it is confusing that the antibodies used here was detecting HA-OTUD1, HA-ASK1 (N) or FALG-ASK1 (N). If the antibody can not specifically detect HA-ASK1 (N), then the conclusion that ectopic expression of OTUD1 enhances ASK1 self-interaction would be wrong. This serious issue needs to be fully clarified.

6. Fig. 6C, the OTUD1 induced OCT4 gene expression seems to be reduced by treatment with DMSO alsone, especially in the ibirutinib experiments. Why is there such a dramatic alteration in its expression upon DMSO treatment? Were the experiments performed in a well controlled condition? I have similar concerns in supp, Fig. 5.

7. The authors suggested that OTUD1-based aggresome stabilize ASK1 and improve its activity, resulting in continuous activation of downstream JNK pathway. These provide an intriguing implication that proteins sequestered in the aggresome could be functional or continuously activated. The authors should examine the localization of activated form of protein involved in the JNK pathway, such as p-ASK1, p-JNK or p-c-Jun in the aggresome.

8. Since the authors argue that ASK1/JNK inhibitors could overcome the resistance of platinum-based chemotherapies, the demonstration of the synergistic cytotoxic effects between two classes of drugs will be important. A quantification and the statistical significance based on several repeated clonogenic assays in Fig. 6E and 6F would strengthen their conclusion.

9. The first sentence in the Abstract, which emphasized the causes of the overwhelmed proteosome capacity in aggresome formation, is redundant and a bit misleading. There is limited data to address whether OTUD1 overexpression overwhelms the proteosome capacity to form aggresome in this study. The data in this study instead indicates that OTUD1 itself is an aggregation-prone protein because of its IDR domain. Therefore, a re-written abstract with more emphasis on the necessities to seek therapeutic strategies to cure ovarian cancer would be more coherent.

Minor Points:
1. Line 106. The stemness-associated genes listed here are different from those presented in its corresponding figure (Supplemental Fig. 1A). This needs to be fixed.
2. Line 380. Is there a redundant word (sequestered)?
3. Fig. 2D, the cell line used for ectopic expression of OTUD1 should ne indicated.
4. In Fig. 3 and Supplemental Fig. 2, the DAPI channel is shown with low contrast, making it very difficult to see the relative localization of the aggresome in cells. A DAPI contrast should be adjusted.

In addition, other cell lines that are more appropriate for imaging (e.g. HeLa, RPE1) should be utilized and presented.

Below is our point-by-point response (in **blue**) to reviewers' comments (in **black**).

Reviewer #1, expertise in ovarian cancer stem cells and models (Remarks to the Author):
Given their focus is on an ovarian serous histophenotype the inclusion of SKOV3 and A2780 cell lines is questionable. These two lines, albeit used extensively in the past for ovarian cancer research have fallen out of favor as optimal models since it has been shown they are less like high grade ovarian serous cancer (Domcke et al., 2013) then more appropriate lines. Moreover, A2780 cells were originally derived from an ovarian endometrioid subtype and are p53 wild type. The OVCAR3 cell line is considered a line more representative of HGSOC. It is highly recommended the investigators expand their cell line repertoire to include more lines representative of HGSOC histophenotype if investigators want to make the claims related to HGSOC.

Response: We sincerely appreciate the reviewer's valuable comments. Following this suggestion, high grade ovarian serous cancer cell lines (OVCAR8 and CAOV3) were employed and the additional experiments with these cell lines were performed. As shown in the revised manuscript, those experiments including sphere formation assay (Figure 1E, Figure 6A, Figure 7D-7E, supplementary Figure 1H, supplementary Figure 6A, supplementary Figure 8B, 8D, 8F), soft agar assay (supplementary Figure 1I and Figure 6C), xenograft assay (supplementary Figure 1K-L and Figure 8G), colony formation assay (Figure 6H-I, Figure 6E, supplementary Figure 6D-G) and, qPCR quantification of CSCs markers (Figure 2C, supplementary Figure 1A, 1E, supplementary Figure 2A, 2B, 2D and supplementary Figure 6C). Consistent with our previous observations, OTUD1 promotes stemness and tumorigenicity in those HGSOC cell lines.

It has long been appreciated that the JNK signaling pathway contributes to the maintenance of CSC like cell in several types of solid tumors. There are also published studies demonstrating that inhibition of the JNK signaling can negatively impact self-renewal and tumor initiating capacity of A2780 CSCs (Okada et al. 2005, Seino et al., 2014 and Seino et al 2016 and should be included). However, the upstream mechanism(s) leading to JNK mediated support of the ovarian cancer stem cells was not known. More specifically the link between OTUD1 and aggresome like organelles promoting ASK1 sequestration and stability activating JNK and subsequently supporting CSCs was not appreciated. This has the potential to move the field forward.

Response: We sincerely appreciate the reviewer's valuable comments. We added more references regarding role of JNK CSCs maintenance (Seino et al., 2014 and Seino et al 2016) in the INTRODUCTION section in the revised manuscript.

In the introduction the authors focus on the epithelium of the ovarian surface epithelium. They point out that it is less differentiated than the epithelium of other anatomical sites. However, it is well accepted now that the bulk of the high grade serous ovarian cancer cases arise in the epithelium of the fallopian tube and not the ovarian surface epithelium. However, there is not reference to this in their manuscript which implies there may be limited background in the field and understanding of the disease itself.

Response: Thank you for pointing this out. We have modified introduction according to the reviewer's suggestion (Introduction, line 51) and appropriate references have been added (Zhang et al. 2019 and Shih et al. 2021).

The authors imply that OTUD1 maintains tumor dedifferentiation but also implies it promotes dedifferentiation – this raises the question - does treatment induced enrichment of CSCs result in further dedifferentiation? Specifically, are the authors implying OTUD1 de differentiates CSCs or are they implying it is causing non-CSCs to take on some stem like properties via dedifferentiation?
Examples: Line 93 …….cancer stem cell de-differentiation in ovarian cancer or Line 144 - 145 ….by which OTUD1 regulates ovarian cancer stemness and dedifferentiation. Their differeing use of the term dedifferentiation makes it challenging to interpret what they mean exactly.

Response: We appreciate reviewer's comment; we have made the necessary revision by changing "ovarian cancer" to "ovarian cancer stem cell" in the revised manuscript (Line 142 in revised manuscript).

Also in their early analyses it was not readily clear what parameters were used for determining a de-differentiated cell from a differentiated cell.

Response: Except for the ability to form floating sphere in the DMEM/F12 medium supplemented with 2% B27 serum replacement in low attachment cell culture plates, the de-differentiated or differentiated state was also determined by using qPCR to quantify the expression of several representative OCSCs genes, such as CD44, OCT4, NANOG, NOTCH1 and SOX2 (Supplementary Figure 1A and 1E.)

Line 122- 123 It is not clear by what the authors mean with regards to the statement ……high grade serous ovarian cancer ………possesses enhanced OCSCs….. Secondarily, are the authors implying that low grade serous ovarian cancer do not use the same mechanisms to maintain their CSCs or do not rely on CSCs?

Response: Bioinformatics analysis showed that the stemness index of high-grade serous adenocarcinoma was higher than that of low-grade serous adenocarcinoma (Supplementary Figure 1F), suggesting that HGSOC cells are less differentiated. Because OTUD1 is expressed in HGSOC tumor tissue with a relatively higher level (Figure 1D）, we speculated that the stemness maintenance of HGSOC stem cells is more likely to rely on the expression of OTUD1. On the other hand, high level OTUD1 predicts the poor prognosis （PFS）in serous ovarian cancer (Figure 1C), so our study does not imply that low-grade serous ovarian cancer do not use the same mechanisms to maintain their CSCs.

The IHC images provided for the HGSOC and LGSC are of poor quality and the staining for OTUD1 is not clear enough to discern organelles. In some instances, the staining appears

diffuse and in others it appears to be localized to the nuclei. Moreover, if the staining is representative of OTUD1 and CSCs as implied then these images would suggest that the bulk of the tumor cells within HGSOCs are CSCs.

Response: We fully agree with the reviewer's suggestion, and we apologize for the low quality IHC image of HGSOC and LGSC, which have been replaced in Figure 1D of the revised manuscript. Although OTUD1 is highly expressed in HGSOCs, this may not suggest that bulk of the tumor cells within HGSOCs are CSCs, we thought that it strongly implied an increased ratio of CSCs and the propensity of de-differentiation in HGSOCs. Furthermore, while most of the OTUD1 localize in cytoplasm, we could not rule out the possibility that residual OTUD1 may be localized to the nuclei, indeed, the analysis of localization of OTUD1 from GeneCards Database (https://www.genecards.org/cgi-bin/carddisp.pl?gene=OTUD1) also indicates that a small fraction of OTUD1 could localize in the nuclei.

The investigators suggest they used CRISPR to knockout OTUD1 yet their immunoblots of the xenograft tumors suggest it was not completely knocked out. Please explain.

Response: This may be due to the contamination of some fibrous connective tissue of a few samples when we obtained the xenograft tumors. We then used other xenograft tumor tissue samples that stored in liquid nitrogen and detected the protein level of OTUD1, as shown in Figure 1J in the revised manuscript.

The authors appear to focus their efforts on stem like markers in SKOV3 cells but not the other lines. Given their implied interest in HGSOCs it would be more relevant to demonstrate the loss knockdown of OTUD1 on CSC markers in the OVCAR3 cells as well as for all cells used herein the present study.

Response: Thank you for your valuable suggestion. We evaluated the effect of OTUD1 depletion on expression of stem cell markers in OVCAR8. The detailed results were shown in Figure 2C and supplementary Figure 2A-2B in the revised manuscript.

Line 80 …… the lack of biomarkers for OCSC-targeted therapy. Please explain given the authors themselves list a number of biomarkers for assessment of OCSC.

Response: Widely described serous ovarian CSC markers include CD44, CD117, CD24, CD133, ALDH1、OCT4、SOX2, NANOG[1]. Several strategies have been proposed to target the OCSC cells[2], for example, the tyrosine kinase inhibitor imatinib mesylate, which targets CD117, PDGFR-α and PDGFR−β, was tested in epithelial ovarian cancer but with poor results as a single agent[3]. The inhibitor 673A preferentially targets CD^{133+} ovarian cancer cells and induces necroptosis by promoting mitochondrial uncoupling protein expression and reducing oxidative phosphorylation[4]. However, none of these compounds are currently being used in clinical trial; one of the major reasons is that most of the anti-CSC therapies target stemness factors that are shared with normal stem cells, compromising their safety. In summary, while a number of biomarkers for assessment of OCSC have been identified, biomarker for OCSC-targeted therapy is still lacking.

Line 161 – Is this protein or mRNA?

Response: Thanks for the reviewer's comments. "ASK1 high expression" has been changed to "ASK1 high mRNA expression" in the revised manuscript.

Line 174 Figure 3 b shows two cell lines, but authors refer to other ovarian lines…. Which ones?

Response: We would like to thank the reviewer's comment. In response to the comments point 1, we deleted the data of A2780 cell lines in the revised manuscript. Meanwhile, to clearly show the effect of OTUD1 on ASK1 level, we constructed DOX-induced EGFP-OTUD1-WT and EGFP-OTUD1-C320A expression plasmid and generated SKOV3 cell lines that could express inducible-OTUD1-WT/OTUD1-C320A to perform western blotting experiments. As shown in the revised Figure 3B, overexpression of both OTUD1-WT and OTUD1- C320A promoted the accumulation of ASK1 protein after 12 hours of DOX induction. As shown in the original Figure 3B (namely supplementary Figure 2F of revised manuscript), OTUD1 knockout led to reduced ASK1 protein levels in SKOV3. In addition, we detected the ASK1 protein level in the OVCAR8 OTUD1 depleted cell and found that OTUD1 knockout obviously reduce the level of ASK1 in OVCAR8 cells (supplementary Figure 2F). Those data were moved to Figure 2B and supplementary figure 2F.

Why did the authors use HEK293 cells for their experiments? Please justify what they would not perform these experiments in ovarian lines for further proof of concept?

Response: Because the transfection efficiency of HEK293 cell is very high**,** it was a suitable tool that can be used to explore the molecular mechanisms, such as detection of protein interaction and identification of the key domains. We have also performed experiments regarding the effect of OTUD1 on tumor cell stemness maintenance in several ovarian cancer cell lines, such as OVCAR3, OVCAR8, CAOV3 and SKOV3.

Lines 271 -276 The flow data results, colony forming and sphere forming data are not accompanied by any statistical supportive data. If the authors are going to make statements related to changes/differences then this information needs to replicated and p values provided.

Response: In response to the reviewers' inquiries regarding the statistical supportive data, we have quantitatively analyzed data of flow cytometry, colony and sphere formation, and the statistical analysis results have been provided in the revised manuscript (Figure 1F, 1G, Figure 6B, 6D, 6G, 6I, Figure 7D, 7E, supplementary Figure 1H, 1I, 1J, supplementary Figure 5A-5C, 5F-5G , supplementary Figure 6B, 6E, 6G, supplementary Figure 7A, 7B, 7D and supplementary Figure 8C, 8E, 8F).

The in vivo data is highly informative. It's unfortunate the ibrutinib treatment was only

shown to be significant on SKOV3 xenografts and not the ovcar3 xenografts which is more representative of the serous phenotype. This implies their conclusions may be over interpreted. This is especially true given the lack of true preclinical models of high grade ovarian cancer.

Response: We sincerely appreciate the insightful comments provided by the reviewer. HGSOCs tend to contain a relatively higher OTUD1 protein level, whereas not for every sample (such as OVCAR3). In response to the reviewers' inquiries regarding preclinical models of high-grade ovarian cancer, we then examine that whether ibrutinib treatment is significant in other representative high-grade serous ovarian cancer cell xenografts. OVCAR8 is a HGSOC cell line and exhibits higher OTUD1 protein level compared with OVCAR3 (supplementary Figure 8A). We therefore performed the xenograft assay to explore the effects of ibrutinib on OVCAR8 xenografts. The results in the revised manuscript clearly showed that OVCAR8 xenografts were much more sensitive to ibrutinib treatment than that of OVCAR3 xenografts (supplementary Figure 8G), which is consistent with the observation in another OTUD1 high ovarian cancer cell line SKOV3. Taken together, ibrutinib treatment is probably more effective in OTUD1-high serous ovarian cancer.

Moreover, given the number of published studies on treatment induced enrichment of CSCs in preclinical models high grade serous ovarian cancer the authors should make the effort to better appreciate how this would alter OTUD1, aggresome formation and subsequent ASK1 signaling.

Response: We sincerely appreciate the insightful comments provided by the reviewer. In response to the reviewers' suggestion. Immunofluorescence assay was performed to explore the effect of ibrutinib on OTUD1 aggresome formation, As shown in Figure R1A, ibrutinib treatment inhibited the OTUD1 aggresome formation. We then detected the protein level of OTUD1, ASK1, p-ASK1, JNK, p-JNK in ibrutinib treated OVCAR8 xenografts tumor tissues. As shown in Figure R1B, ibrutinib decreased their level. We speculated that JNK signaling pathway, which is inhibited by ibrutinib, is also essential for OTUD1 expression. The exact molecular mechanisms by which ibrutinib regulates OTUD1 expression will be investigated in our future study.

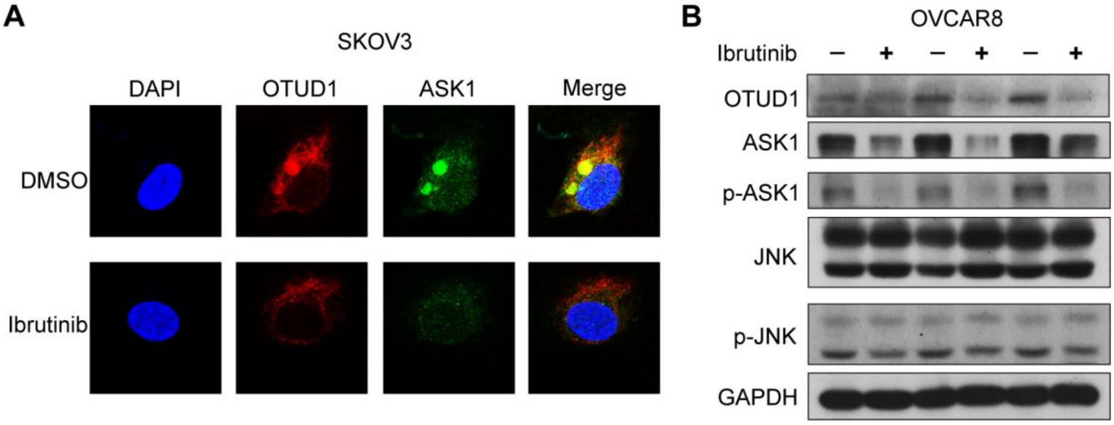

Figure R1. Ibrutinib treatment could inhibit OTUD1-based aggresome formation and ASK1 signaling pathway

(A) The effect of ibrutinib on OTUD1-based aggresome formation was evaluated by the immunofluorescence assay in SKOV3 cells.
(B) The protein level of OTUD1, ASK1, p-ASK1, JNK and p-JNK in ibrutinib treated OVCAR8 xenografts tumor tissues.

Line 423 – While targeting OTUD1 is of interest there is not enough data provided to support the fact the statement as a therapeutic target for high grade serous ovarian cancer.

Response: Thanks for the reviewer's comments. To provide more evidence that OTUD1 was a therapeutic target in high grade serous ovarian cancer, we performed additional experiments in more high grade serous ovarian cancer cell lines. Specifically, we knockout OTUD1 in HGSOC cell lines OVCAR8 and CAOV3 and measured the sphere formation ability and anchorage independent growth ability (Figure 1E-F and supplementary Figure 1H-1I). Next, we examined the effect of OTUD1 depletion on ASK1 signaling pathway (supplementary Figure 2F). Moreover, we performed the xenograft with OVCAR8 OTUD1 knockout cells (supplementary Figure 1L-1M). We have also evaluated the effect of ibrutinib on OTUD1-high HGSOC cells (Supplementary Figure 6). In addition, we performed experiments to explore whether JNK specific inhibitor (IN-8)/ASK1 inhibitor (selonsertib) or ibrutinib treatment could improve efficacy of platinum-based (Cisplatin, DDP) chemotherapy in OVCAR8 cells and CAOV3 cells. As shown in Figure 6H-6I and supplementary Figure 6D-6G in the revised manuscript, OTUD1 expression could reduce the sensitivity of SKOV3, OVCAR8 and CAOV3 cells to chemotherapeutic, while combination of ASK1/JNK pathway inhibition and platinum-based chemotherapy could enhance the chemotherapeutic efficacy. Those new results help to reinforce the ideal that OTUD1 might serve as a therapeutic target for high grade serous ovarian cancer.

Reviewer #2, expertise in the MAPK/JNK pathway (Remarks to the Author):

This paper investigates the mechanism underpinning ovarian cancer cell de-differentiation. The data show that ovarian cancer cells exhibiting characteristics of stem cells display increased expression of the deubiquitinase OTUD1 that leads to the formation of aggresome-like organelles. The stabilization of ASK1 following its recruitment to these organelles via direct interaction with OTUD1 activates JNK. ASK1/JNK activation associated with elevated OTUD1 expression contributes to maintaining ovarian cancer stem cells (OCSCs) tumorigenic properties.
Opinion: The paper contains a large amount of interesting data. However, I have raised a number of main concerns that must be addressed prior to considering the paper for publication in Nature Communications.

1. In addition to providing evidence that different ovarian cancer cell lines (i.e. OVCAR3 and SKOV3) exhibit different levels of OTUD1 protein (Figure 7A), the authors must demonstrate that the protein level of OTUD1 in floating SKVO3 spheres (i.e. SKOV3 cells seeded in ultra-low-attachment plates) is higher compared with that in differentiated cells (i.e. SKOV3 spheres re-cultured in plates). These data should be included in Figure 1 to confirm that OTUD1 is important for maintaining ovarian cancer stem cell characteristics.

Response: We deeply appreciate the reviewer's insightful comments. Following the reviewer's suggestion, we collected floating SKVO3 spheres and measured protein level of OTUD1 (Figure 1B in revised manuscript)， the result showed that OTUD1 protein level is upregulated in floating spheres. We have also determined the OTUD1 protein level in OVCAR8 floating spheres, which is a HGSOC cell lines. As shown in supplementary Figure 1D, OTUD1 protein level is upregulated in OVCAR8 floating spheres.

2. Figure 2D: The apparent very high level of ectopic expression of OTUD1 in SKOV3 raises concern about the possibility of artefacts associated with transfection of expression vectors to study protein functions, in particular as a consequence of overwhelming endogenous protein sorting processes. Therefore, the authors must repeat this experiment using an inducible system (e.g. the dox-inducible EGFP-OTUD1 plasmid they used in Figure 3H) that would permit to achieve a level of ectopic OTUD1 expression similar to that observed in spheres (see my comment above). This should be done even if the OTUD1M1 mutant does not appear to form aggresomes (Figure 4F), despite expressing at the same level as the wild type protein (Figure 4D/4H). A similar criticism can be made when interpreting the data obtained based on ASK1 overexpression (Figure 3).

Response: Thanks for the reviewer's comments. In response to reviewer's inquiry regarding the OTUID1 expression level, we re-performed the experiments by using the dox-inducible EGFP-OTUD1 expression system. As shown in Figure 2D, Figure 3B and Figure 4H, dox-inducible ectopic OTUD1 WT, C320A or M1 mutant expression levels were similar to that observed in spheres (Figure 1B) was achieved at 12 hours post induction (2-3 folds increased). Consistently, expression of OTUD1 WT and C320A mutant but not M1 mutant are sufficient to causes the aggresome formation and activate ASK1 pathway (Figure 3C and Figure 4F-4G). In terms of the ASK1 overexpression in Figure 3, we noticed that ASK1 was overexpressed in all the groups, however, ASK1 protein aggregates only when the ectopic OTUD1 was present, suggesting that ASK1's recruitment to aggresome is unlikely to be influenced by its expression level.

3. Figure 2 Panels A and B: The justification for focusing on JNK signalling is weak for a number of reasons:
a) The role of JNK in CSC remains controversial with some studies suggesting that JNK does not affect CSC activity, while others indicating that JNK contributes to maintaining CSC properties. Hence, the authors must revise their statement that: "MAPK/JNK activation has been frequently implicated in CSC activity regulation in various cancer types" (line 150-151) to reflect these conflicting findings. Also, a reference should be added to support the following statement: "Because the active JNK pathway has been shown to be involved in CSC maintenance by numerous studies,…" (line 262).

Response: Thanks for the reviewer's suggestion. We have changed statement to "MAPK/JNK activation has been implicated in ovarian CSC activity regulation, whereas its roles may vary in different cancer types" in the revised manuscript (lines 148-149). As suggested by the reviewer, we have added more references to support the statement (Yoon et al., 2012, Seino et al., 2014,

Girnius et al., 2018, and Ohta et al. 2013).

b) The list of genes regulated by OTUD1 and presented in panel B [a mixture of markers of CSC identity together with transcription factors and MAPK6 (ERK3), MAP2K1 (MEK1), MAPK3K8)], does not comprise any particularly convincing JNK targets.

Response: We thank our reviewer for pointing this out. To confirm the specific effect of OTUD1 on JNK signaling pathway, we detected the phosphorylation levels of several key components of the MAPK signaling pathway, and the results revealed that only the p-JNK level but not p-ERK or p-p38 was significantly reduced after knocking out OTUD1 in SKOV3 cells (Figure 2B in revised manuscript).

c) Line 149: "Given the smaller P value of the MAPK pathway compared with those of the other pathways…" which P value are the authors referring to? Which MAPK pathway? What about JNK? Is the P value the most appropriate to select a specific pathway? I am not convinced.

Response: We appreciate the reviewer's constructive criticism. P value we referred to here is generated by clusterProfiler when performing KEGG enrichment analysis, which is used to indicate the pathways that have been enriched are statistically significant. Our initial KEGG enrichment suggested that OTUD1 was closely related to the MAPK pathway. Because among the MAPK pathways, JNK signaling has been shown to be involved in CSCs stemness maintenance by many studies, and our results implied that OTUD1 plays an important role in CSCs stemness maintenance (Figure 1), we then hypothesized that OTUD1 depletion might impairs the JNK signaling (Figure 2B). We agreed that the P value is not the only criteria for selecting a specific pathway. To test our hypothesis, we further detected p-ERK, p-p38 and p-JNK level in OTUD1 knockout cells, the result showed that only p-JNK was significantly downregulated (also see our response to Point 3b), suggesting that the JNK should be the appropriate one in enriched signaling pathways.

To address this issue the authors must re-analyze their RNA-seq data to identify known JNK target genes in addition to FOS. These data are critical to confirm the possibility that the level of OTUD1 determine the level of JNK activity. In addition, the legend of Figure 2A should be revised to clarify how the graph was produced and detail of the strategy for knocking out the OTUD1 locus in ovarian cancer cells by CRISPR-Cas9 should be provided. In particular, sequencing of the probes must be provided.

Response: We fully agree with reviewer's suggestion. We re-analyzed RNA-seq data and found that in addition to FOS, several reported JNK target genes[5-7] were downregulated upon OTUD1 depletion. We then performed qPCR to verify their expression in OTUD1 knockout cells (Figure 2C). Besides, given that a number of CSCs markers have been shown to be JNK target[6], we also measured their transcription in the same cell samples, the results revealed that their expression were downregulated as well (supplementary Figure 2A-2B). We revised the legend of Figure 2A and provided the details of the strategy for knocking out the OTUD1 locus in ovarian cancer cells

by CRISPR-Cas9 in the vectors and plasmids production of method. The sequencing of the sgRNA-OTUD1 probes were also provided in table S1.

4. Figure 6 and 7D-G: The interpretation of these data acquired from analysing the effect of SP600125 and ibrutinib is flowed because these are not specific JNK inhibitors. Therefore, the panels where SP600125 and ibrutinib are employed must be moved to supplementary data and interpreted in light of their non-specific effect. Where appropriate, these experiments must be repeated by using the ASK1 specific inhibitor selonsertib.

Response: Thank you for your valuable suggestion. As requested by the reviewer, the panels where SP600125 was used have been moved to supplementary Figure 7. In addition, we repeated the experiments by using a specific JNK inhibitors (IN-8) and a ASK1 specific inhibitor selonsertib (please see Figure 6, supplementary Figure 6, Figure 7D-7E and supplementary Figure 8B-8F in the revised manuscript). The results revealed that OTUD1 promote floating sphere formation and anchorage independent growth, while the promoting effect was significantly compromised by administration of JNK specific inhibitor IN-8, ASK1 specific inhibitor selonsertib or ibrutinib (Figure 6A-6D and supplementary Figure 6A-6B). In addition, we performed experiments to explore whether IN-8, selonsertib, and ibrutinib could improve the platinum based (Cisplatin, DDP) chemotherapy efficacy in OVCAR8 and CAOV3 cells caused by ectopic OTUD1. Interestingly, treatment with either IN-8, selonsertib or ibrutinib significantly eliminated the enhanced the sensitivity of cells to cisplatin (Figure 6H-6I and supplementary Figure 6D-6G). We have also treated spheres of several ovarian cancer cells with IN-8, ibrutinib and selonsertib, the results showed that those inhibitors more effectively inhibited sphere formation in OTUD1-high cancer cells (Figure 7D-7E and supplementary Figure 8B-8F). Although ibrutinib may not be a specific JNK inhibitor, it was a FDA approved drug with tolerable toxicity, we therefore focused on the effect of ibrutinib on tumorigenicity to explore the translational potential for patients with OTUD1-high ovarian cancer.

5. The removal of the data based on the use of SP600125 and ibrutinib will inevitably weaken the demonstration that JNK determines ovarian cancer stem cell phenotypes. Therefore, to firmly establish a link between JNK and the maintenance of stemness, the authors must provide experimental evidence that JNK-mediated c-Jun activation transcriptionally regulates some of the markers of ovarian cancer stem cells listed in Figure 7I.

Response: We deeply appreciate the reviewer's insightful comments. T-5224 (T-5) is a selective c-Jun inhibitor that has been shown to inhibit lymph node metastasis in oral cancer and high-grade serous ovarian carcinoma[8,9]. qPCR was used to confirm whether JNK-mediated c-Jun activation transcriptionally regulates some of the OCSCs markers. As shown in supplementary Figure 2D, T-5224 treatment significantly compromised the promoting effect of ectopic OTUD1 on expression of key CSC genes (*CD44*, *OCT4*, *SOX2*, *NANOG*).

6. Supplementary Figure 3A: The data presented in the lower panel do not make any sense. The HA antibody does not detect HA-OTUD1(N) in the first input (lane 1). The Flag

antibody detects a protein in the anti-Flag immunocomplex from cells that do not express Flag-tagged OTUD1(N). Please clarify.

Response: Firstly, we want to apologize for the mistake we have made in Supplementary Figure 3A, those mislabeling have been corrected in the revised manuscript (Supplementary Figure 4A in the revised manuscript).

7. Supplementary Figure 3F shows that the JNK/c-Jun pathway was inhibited after knocking out OTUD1. These data should be moved to where the hypothesis that JNK is a downstream target of OTUD1 is first described (Fig. 2D).

Response: Thanks for the reviewer's suggestion. The Supplementary Figure 3F has been moved to Figure 2B in the revised manuscript.

8. Figure 4H: Expression of Δ105-164 OTUD1 mutant appears to decrease ASK1 expression and inhibit ASK1 and JNK phosphorylation, without affecting the phosphorylation of c-Jun. Based on the hypothesis that c-Jun is a substrate of JNK, this observation does not make any sense. Please explain.

Response: Thanks for the reviewer's comments. We repeated this assay with an inducible system. As shown in Figure 4H in the revised manuscript, while overexpression of OTUD1 WT strongly promotes ASK1 expression and increased ASK1/JNK/c-Jun phosphorylation, M1 (Δ105-164) mutant decreased ASK1 protein level and inhibited ASK1, JNK and c-Jun phosphorylation. Given that M1 mutant destroy the aggresome formation and could not sequester ASK1, one of the possibility is that loss of aggregation of ASK1 promotes its degradation.

9. Figure 4I is confusing because the panels with antibodies are mislabelled. Also, how is Ha-OTUD1 distinguished from Ha- ASK1(N). I presume that these two proteins exhibit different molecular weight, but this is not clear.

Response: Thank you for pointing this out. We want to apologize for the confusion caused by our mistake that the HA-OTUD1 band was not shown in original Figure 4I. We have changed the figure in the revised manuscript (Figure 4I). It is true that HA-OTUD1 and HA-ASK1(N) exhibit different molecular weight (HA-OTUD1 is about 55-60kd; HA-ASK1(N) is over 100kd).

10. Figure 4J: Similarly, I presume that the Flag panel shows Flag-ASK1 ad not Flag-OTUD1 (WT or mutant). This must be clarified.

Response: Thanks for pointing this out. We are very sorry because HA and Flag antibodies were mislabeled and they were in the wrong order. Besides, the Flag-OTUD1 and Flag-M1 bands were missing. Those mistakes have been corrected in the revised manuscript (Figure 4J).

11. Most figure legends must be revised to clarify experimental designs and data presentations.

Response: Thanks for the reviewer's suggestion. All figure legends were revised to clarify experimental designs and data presentations.

Reviewer #3, expertise in aggresome formation (Remarks to the Author):

This work detailed in this manuscript concerns the identification of biomarkers for OCSC-targeted therapy. Here, Chen et al. performed multi-omics screening to identify OTUD1 as a key factor of the OCSC dedifferentiation and tumorigenesis. A role for OTUD1 expression in maintaining stemness and promoting tumorigenic activity of OCSC is described. OTUD1 overexpression was shown to induce aggresome formation, which is responsible for the stabilization of the key JNK component, ASK1, which leads to the activation of the JNK pathway to potentiate OCSC stemness maintenance and tumorigenicity. Importantly, JNK pathways inhibitors, including the FDA-approved ibrutinib, suppressed the OTUD1-induced OCSC stemness maintenance, tumorigenesis and chemoresistance, highlighting the potential of combining JNK inhibitors and standard chemotherapeutics to treat ovarian cancer with OTUD1 overexpression or OCSC maintaining ability.

Mechanistically, they find that OTUD1 overexpression induced aggresome formation, which sequesters ASK1, resulting in its stabilization and enhanced activity that drives JNK pathway activation, thus maintaining OCSC stemness. The authors demonstrated that mutating the N-terminal intrinsically disordered region (IDR) of OTUD1, shown to be critical for OTUD1-based aggresome formation in the study, decreased ASK1 activation, disrupted OCSC stemness maintenance and reduced the tumorigenicity of xenotransplanted SKOV3 cells. The findings detailed in this manuscript are generally novel. Aggresome formation is a well-recognized hallmark of neurodegenerative diseases but its role in cancer is less well understood. Additionally, proteins sequestered in the aggresome are generally thought to be inactive, leading to the loss of functions of certain proteins. The authors show that aggresome formation can activate a cancer signaling pathway and provide novel insights on the role of aggresome in the pathology of cancer. This work also provides a novel example that proteins sequestered in the aggresome could be functional to illicit downstream cellular effects.

Despite these many novelties, I do have some concerns about the data and some key quantifications are missing, which dampens my enthusiasm about some of the major conclusions made by the authors. Additionally, some sections of the main text are not presented logically and should reworked to make the article easier to follow. The following major revisions need to be carried out prior to consideration for publication:

1. In Fig. 1, Fig. 2 and Supplemental Fig. 1, the sgRNA-mediated knock out of OTUD1 in SKOV3 and A2780 cells reduced stemness, sphere formation and colony formation. The authors need to perform rescue experiments using WT- and M1-OTUD1 re-expression in these contexts to exclude possible off-target effects and requirement of IDR domain. This would make the model proposed in this study more convincing.

Response: We appreciate reviewer's insightful comments. Following reviewer's suggestion, we performed rescue experiments by re-expressing OTUD1 WT-or M1 mutant in OTUD1 depleted cells. As shown in supplementary Figure 5E-5G, WT-OTUD1 but not the M1 mutant re-expression increased sphere formation and colony formation ability.

2. In Supplemental Fig. 1D, the decrease of OTUD1 level in differentiated A2780 cells seems marginal, which may compromise the conclusion regarding the roles of OTUD1 in OCSC stemness maintenance. It would be more convincing to show densitometric quantification of the band intensity and the statistical significance of the decrease based on several biological replicates.

Response: Thanks for the reviewer's comments. We repeated this experiment by extending the culture time in in the DMEM/F12 medium supplemented with 2% B27 serum replacement. As shown in Figure R2, A2780 sphere exhibited higher OTUD1 protein level. Because A2780 is not a very typical serous ovarian cancer cell line (See Point 1, Reviewer 1), we have deleted A2780 data in the revised manuscript. On the other hand, we performed floating sphere formation experiments in more representative HGSOC cell lines, such as OVACAR8, and we detected the protein level of OTUD1 in floating OVACAR8 spheres (supplementary Figure 1C).

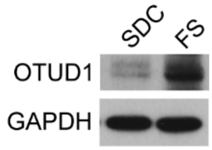

Figure R2. OTUD1 level was increased in A2780 cells floating spheres.
SDC, Shpere Differentiated Cell. FS, Floating sphere.

3. Fig. 2F, why was the FLAG epitope detected in the FLAG-IP sample purified from cells that are not expressing FLAG-OTUD1? Is it an unspecific band? Also, HA is not detected in the input sample from cells expressing HA-ASK1, which is very confusing. Were the ectopic expression and the IP procedures successful?

Response: Thanks for the reviewer's comments. We want to apologize for mislabeled antibodies, which have been corrected in the revised manuscript (please see the Figure 2E in the revised manuscript).

4. On line 195-201, the rationale to perform nocodazole treatment to confirm the formation of OTUD1-based aggresome is not logically explained. Microtubule dynamics is known to promote aggresome formation (doi: 10.1385/JMN:29:2:153), but the rationale here emphasized the overwhelmed proteolysis system as the cause of aggresome formation. A rephased rationale and a more appropriate citation would be preferred.

Response: We fully agreed with reviewer's suggestion. We have rephased the rationale of nocodazole (NOCO) in our manuscript (Line 209-210 in the revised manuscript), now the rationale is "which is essentially rely on the presence of microtubule". In addition, more appropriate reference to support the statement have been included (Webb et al. 2004, Nina et al.

2006).

5. In Fig. 4I, FLAG-IP was performed using HEK293T cells expressing FLAG-ASK1 (N), HA-ASK1 (N) and HA-OTUD1. Since both OTUD1 and ASK1 (N) were tagged with HA, it is confusing that the antibodies used here was detecting HA-OTUD1, HA-ASK1 (N) or FALG-ASK1 (N). If the antibody cannot specifically detect HA-ASK1 (N), then the conclusion that ectopic expression of OTUD1 enhances ASK1 self-interaction would be wrong. This serious issue needs to be fully clarified.

Respones: Thanks for the reviewer's comments. We want to apologize for the confusion caused by our mistake that the HA-OTUD1 band was not shown in Figure 4I. We have changed the figure 4I in the revised manuscript (Figure 4I). Please see reviewer #2 point 9.

6. Fig. 6C, the OTUD1 induced OCT4 gene expression seems to be reduced by treatment with DMSO alsone, especially in the ibirutinib experiments. Why is there such a dramatic alteration in its expression upon DMSO treatment? Were the experiments performed in a well controlled condition? I have similar concerns in supp, Fig. 5.

Response: Thanks for the reviewer's comments. In fact, DMSO treatment did not influence the OCT4 gene expression, because when we analyzed the effect of ibrutinib on OTUD1-expressing cells, DMSO group was employed as control, respectively. To better display the effect of ibrutinib, we re-performed the experiments and used empty vector group as control. The results revealed that ibrutinib treatment blunted the promoting effect of OTUD1 on CSCs markers expression, which remained unchanged in the presence of DMSO (Figure 6E and supplementary Figure 6C of the revised manuscript).

7. The authors suggested that OTUD1-based aggresome stabilize ASK1 and improve its activity, resulting in continuous activation of downstream JNK pathway. These provide an intriguing implication that proteins sequestered in the aggresome could be functional or continuously activated. The authors should examine the localization of activated form of protein involved in the JNK pathway, such as p-ASK1, p-JNK or p-c-Jun in the aggresome.

Response: Thanks for the reviewer's insightful comments. Following reviewer's suggestion, we performed the immunofluorescence experiment to determine the localization of p-ASK1 and p-JNK protein upon OTUD1 expression. As shown in supplementary Figure 4F-4G in the revised manuscript, enhanced p-ASK1 and p-JNK signal intensity were observed. Moreover, we found that both p-ASK1 and p-JNK were enriched in OTUD1 WT based aggresome, while deletion of OTUD1105-164aa (M1) failed to sequester p-ASK1 and p-JNK, suggesting that those proteins sequestered in the aggresome could be functional or continuously activated.

8. Since the authors argue that ASK1/JNK inhibitors could overcome the resistance of platinum-based chemotherapies, the demonstration of the synergistic cytotoxic effects between two classes of drugs will be important. A quantification and the statistical significance based on several repeated clonogenic assays in Fig. 6E and 6F would strengthen

their conclusion.

Response: Thanks for the reviewer's comments. The quantification and the statistical significance analysis of several repeated clonogenic assays were performed in the revised Figure 6B, 6F and 6I. As shown in the Figure 6B, 6F and 6I of revised manuscript, ibrutinib and cisplatin indeed exhibited a synergistic effect because their combination inhibited tumor cell colony formation to a greater extent than either of them alone. The similar results were also obtained in OVCAR8 cells (supplementary Figure 6D-6G).

9. The first sentence in the Abstract, which emphasized the causes of the overwhelmed proteosome capacity in aggresome formation, is redundant and a bit misleading. There is limited data to address whether OTUD1 overexpression overwhelms the proteosome capacity to form aggresome in this study. The data in this study instead indicates that OTUD1 itself is an aggregation-prone protein because of its IDR domain. Therefore, a re-written abstract with more emphasis on the necessities to seek therapeutic strategies to cure ovarian cancer would be more coherent.

Response: Thanks for the insightful comments. We re-written the abstract in the revised manuscript according to reviewer's suggestion.

10. Line 106. The stemness-associated genes listed here are different from those presented in its corresponding figure (Supplemental Fig. 1A). This needs to be fixed.

Response: We modified stemness-associated genes listed in line 104 to make sure that they the same as the genes in supplementary Figure 1A.

11. Line 380. Is there a redundant word (sequestered)?

Response: Thanks for pointing this out. We have deleted the redundant word (sequestered) in line 398.

12. Fig. 2D, the cell line used for ectopic expression of OTUD1 should ne indicated.

Response: Thanks for the reviewer's comments. We have added the cell line information for ectopic expression of OTUD1 at the bottom of Figure 2D.

13. In Fig. 3 and Supplemental Fig. 2, the DAPI channel is shown with low contrast, making it very difficult to see the relative localization of the aggresome in cells. A DAPI contrast should be adjusted. In addition, other cell lines that are more appropriate for imaging (e.g. HeLa, RPE1) should be utilized and presented.

Response: We fully agreed with the reviewer. We have adjusted DAPI contrast in Figure 3 and Supplemental Figure 3 (original supplemental Figure 2). In addition, we performed immunofluorescence experiments in HeLa cells. As shown in Figure R3, expression of the Δ 105 164

(M1) but not OTUD1 WT resulted in OTUD1 aggresome formation in Hela cells.

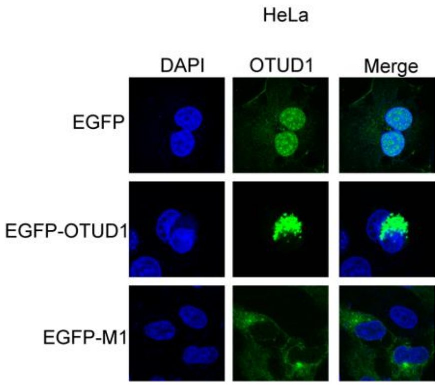

Figure R3. The localization of the OTUD1-based aggresome in HeLa cells**.**

References

1 Burgos-Ojeda, D., Rueda, B. R. & Buckanovich, R. J. Ovarian cancer stem cell markers: prognostic and therapeutic implications. *Cancer Lett* **322**, 1-7 (2012).

2 Munoz-Galvan, S. & Carnero, A. Targeting Cancer Stem Cells to Overcome Therapy Resistance in Ovarian Cancer. *Cells* **9** (2020).

3 Schilder, R. J. *et al.* Phase II evaluation of imatinib mesylate in the treatment of recurrent or persistent epithelial ovarian or primary peritoneal carcinoma: a Gynecologic Oncology Group Study. *J Clin Oncol* **26**, 3418-3425 (2008).

4 Chefetz, I. *et al.* A Pan-ALDH1A Inhibitor Induces Necroptosis in Ovarian Cancer Stem-like Cells. *Cell Rep* **26** (2019).

5 Liu, Y., Zhang, X., Wang, J., Yang, J. & Tan, W.-f. JNK is required for maintaining the tumor-initiating cell-like properties of acquired chemoresistant human cancer cells. *Acta Pharmacol Sin* **36**, 1099-1106 (2015).

6 Xie, X. *et al.* c-Jun N-terminal kinase promotes stem cell phenotype in triple-negative breast cancer through upregulation of Notch1 via activation of c-Jun. *Oncogene* **36**, 2599-2608 (2017).

7 Balko, J. M. *et al.* Activation of MAPK pathways due to DUSP4 loss promotes cancer stem cell-like phenotypes in basal-like breast cancer. *Cancer Res* **73**, 6346-6358 (2013).

8 Kamide, D. *et al.* Selective activator protein-1 inhibitor T-5224 prevents lymph node metastasis in an oral cancer model. *Cancer Sci* **107**, 666-673 (2016).

9 Hao, Q. *et al.* Single-cell transcriptomes reveal heterogeneity of high-grade serous ovarian carcinoma. *Clin Transl Med* **11**, e500 (2021).

Reviewers' Comments:

Reviewer #1:
Remarks to the Author:
In general, the manuscript remains of interest. The authors dod address some of the issues raised. Unfortunately, there remain some concerns and the data doesn’t always support the author's conclusions. The authors basically imply OTUD1 is universally promoting or supporting the maintenance of ovarian CSCs but the results of their experiments suggest it may be limited in its role in specific lines.

Independent of the science the manuscript has an abundance of spelling/grammatical errors. Some of the sentences are awkward and challenging to follow. There are too many to list. It is recommended they recruit someone to assist.

Despite the blanket claims that OTUD1 promotes or maintains stemness in ovarian cancer. It is not entirely clear how the authors defend how ovarian cancer cell lines with no to low levels of OTUD1 maintain their stem-like features. Alternatively, are the authors implying there are a subset of ovarian cancer cell lines or ovarian high-grade serous cancer cells that express elevated levels of OTUD1 which results in a greater abundance of stem-like cells? The inhibitor actions or knockdown strategies appear to influence the OTUD1 induced increase but not necessarily the baseline levels.

The fact that the highest levels of OTUD1 are evident in the SKOV3 line doesn’t provide a lot of confidence this is a universal player in all high-grade serous cell lines.

The authors need to explain more comprehensively what they mean by the term ovarian cancer stem cell dedifferentiation. Are they implying that ovarian CSCs are further dedifferentiating or are they implying non CSCs are becoming CSCs?

Line 143 – Based on the data provided, it is not clear how OTUD1 plays a role in dedifferentiation.

Line 276 - What is the difference in tumorigenicity and stemness? Isn’t tumorigenicity one of the readouts for stemness?

Line 285 – how was “enhanced” tumorigenicity impaired exactly?

Line 291- How did WT OTUD1 promote chemosensitivity?

Line 323 – How do you enhance maintenance?

Lines 425 – 429 Given the range by which the different cell lines were impacted, this is a bold statement.

Lines 439 – 440 As presented the summary statement is not supported by the data provided and the conclusion should be toned down.

Line 574 -5 What are distal ovarian tissues? Please define.

Are the supplementary figures 1 and 2 duplicated?

Reviewer #2:
Remarks to the Author:
The authors have addressed all my concerns.

Reviewer #3:
Remarks to the Author:
Summary:
All the concerns raised were properly addressed in the rebuttal letter and the revised manuscript. There are some minor issues in the revised manuscript, listed as follows:
Remaining issues:
1. Line 29 in the Abstract: which is mediated by a short peptide locates in …" (located?)

2. Line 50-51 in the Introduction: "While the high grade serous ovarian cancer (HGSOC), which is the most the common histotype in the clinic, was believed to arise from the fallopian tube." Gramatical error?

3. Line 126-139 and the supplementary Fig. 1: It seems like there are mistakes in the figure labeling (all the labels are shifted alphabetically?)

4. Supplementary Fig 2 seems to be identical to supp. Fig. 1. Mistakes while uploading?

5. Line 205-206: "We then hypothesized that if the aggregation was an aggresome, which is normally formed relied on the presence of microtubule" (relying?)

6. Line 240 and Supplementary Fig. 4A, these labels of the OTUD constructs are a bit confusing. aa 287-481 was termed N and aa 1-286 was termed C? maybe N∆ and C∆ respectively?

7. Line 252: ", expression of the Δ105-164 (M1) but not OTUD1WT significantly reduced cellular OTUD1 aggresome formation and co-localized ASK1 aggregation." This is a bit imprecise description to me. Maybe "ectopically expressed Δ105-164 (OTUD-M1) did not form aggresome colocalized with ASK1, as observed for OTUD1WT" or " deletion of aa 105-164 disrupted the tendency of OTUD1 to form aggresome colocalized with ASK1".

8. Line 324 and supplementary Fig 7: is there a mistake in the fig indication? (Supplementary Fig. 7D rather then C?) To support the conclusion "OTUD1 expression reduced the sensitivity of SKOV3, OVCAR8 and CAOV3 cells to cisplatin treatment…", the author need to compare the OTUD1 expression + cisplatin (DPP) group to the empty vector (EV) + DPP control. However, I only saw EV + DPP in the statistic plot but not in the corresponding image of Supp. Fig. 7D.

Below is our point-by-point response (in blue) to reviewers' comments (in black) for your consideration.

Reviewer #1 (Remarks to the Authors):

In general, the manuscript remains of interest. The authors dod address some of the issues raised. Unfortunately, there remain some concerns and the data doesn't always support the author's conclusions. The authors basically imply OTUD1 is universally promoting or supporting the maintenance of ovarian CSCs but the results of their experiments suggest it may be limited in its role in specific lines.

Independent of the science the manuscript has an abundance of spelling/grammatical errors. Some of the sentences are awkward and challenging to follow. There are too many to list. It is recommended they recruit someone to assist.

Response: We thank our reviewer for pointing this out, we invited a native speaker to help us proofread the manuscript to minimize typographical and grammatical errors.

Despite the blanket claims that OTUD1 promotes or maintains stemness in ovarian cancer. It is not entirely clear how the authors defend how ovarian cancer cell lines with no to low levels of OTUD1 maintain their stem-like features. Alternatively, are the authors implying there are a subset of ovarian cancer cell lines or ovarian high-grade serous cancer cells that express elevated levels of OTUD1 which results in a greater abundance of stem-like cells? The inhibitor actions or knockdown strategies appear to influence the OTUD1 induced increase but not necessarily the baseline levels.

Response: We sincerely appreciate the reviewer's valuable comments. While our results indicated that high-level OTUD1 and associated MAPK/JNK signaling activation promotes ovarian cancer cell stemness, it does not mean that every serous ovarian cancer cell line rely on this mechanism to maintain the stemness of OCSCs. Like normal tissue stem cells, CSCs similarly exhibit significant phenotypic and functional heterogeneity[1]. Previous studies have shown that several signaling pathways, such as WNT, Notch, MPAK and SHH are associated with ovarian cancer stem cell properties[2], implying that OCSCs stemness could be maintained through multiple mechanisms. For example, our results showed that OTUD1 protein level is much higher in SKOV3 and OVCAR8 cells compared to OVCAR3 cells, and those OUTD1-high cells are more sensitive to JNK signaling inhibitor treatment (Figure 7 and supplementary Figure 8). In contrast, notch signaling pathway protein Notch3 is highly expressed in OVCAR3 but not in SKOV3 cells, and targeting Notch3 specifically sensitize OVCAR3 cells to chemotherapy[3]. We treated spheres of ovarian cancer cells with IN-8, ibrutinib and selonsertib, and the promoting effect of OTUD1 on sphere formation were compromised in response to those treatment (Figure 6 and supplementary Figure 6). Meanwhile, those inhibitors more effectively suppressed sphere formation of ovarian cancer cells with higher level of OTUD1 (Figure 7 and supplementary Figure 8). Those results imply that JNK inhibition in OTUD1-high serous ovarian cancer for attenuating CSCs stemness might be a new personalized therapeutic approach.

The fact that the highest levels of OTUD1 are evident in the SKOV3 line doesn't provide a lot of confidence this is a universal player in all high-grade serous cell lines.

Response: In the revised manuscript, we have employed several high-grade serous cell lines and found that OVACAR8 also contains a relatively higher protein level of OTUD1, which is comparable to that of SKOV3. As we have mentioned before, while high-grade serous ovarian cancer tissues are more likely to exhibit higher level of OTUD1, we could not rule out the possibility that individual sample of other subtypes or undefined subtypes also contain abundant OTUD1. In addition, high level of OTUD1 predicts poor prognosis in serous ovarian cancer (Figure 1C, Supplementary Figure 1B). We speculated that OTUD1 is important for CSCs stemness maintenance in high-grade serous ovarian cancer as well as a portion of other serous ovarian cancers.

The authors need to explain more comprehensively what they mean by the term ovarian cancer stem cell dedifferentiation. Are they implying that ovarian CSCs are further dedifferentiating or are they implying non CSCs are becoming CSCs?

Response: Cancer stem cells (CSCs) are a subgroup of tumor cells that are capable of self-renewal and differentiation into non-tumorigenic progeny. In this study, we isolated stem-like cells from ovarian cell lines under standard conditions, namely, by culturing the tumor cells in the DMEM/F12 medium supplemented with 2% B27 serum replacement. Because certain environmental stimuli were required (such as when the tumor cells were co-inoculated with irradiated cells) for transition from non CSCs to CSCs[4], the term cancer stem cell dedifferentiation here actually means maintaining CSCs stemness. To avoid any confusion, we can replace "dedifferentiation" with "stemness maintenance".

Line 143 – Based on the data provided, it is not clear how OTUD1 plays a role in dedifferentiation.

Response: We appreciate reviewer's comment. As shown in Figure 1, multiomics-based screening revealed that OTUD1 is a biomarker of poor prognosis and is associated with cancer stem cell stemness maintenance in ovarian cancer. To precisely explain our results, we changed "ovarian cancer stem cell stemness and dedifferentiation" to "ovarian cancer stem cell stemness" in line 142 of the revised manuscript (namely, line 143 of the original manuscript). Moreover, the RNA-seq result in Figure 2A implied that OTUD1 regulates stemness through MAPK pathway.

Line 276 - What is the difference in tumorigenicity and stemness? Isn't tumorigenicity one of the readouts for stemness?

Response: We agree with the reviewer that tumorigenicity is one of the readouts for stemness. Because in this section, we measured the tumor formation ability and the stem cell properties, so we mentioned both of them in the subtitle.

Line 285 – how was "enhanced" tumorigenicity impaired exactly?

Response: Thanks for the reviewer's comments. We apologize for the lack of clarity in line 285 of the original manuscript. "enhanced" was changed to "induced" in line 286 of the revised manuscript.

Line 291- How did WT OTUD1 promote chemosensitivity?

Response: Thanks for pointing this out. We deleted the redundant words (and sensitivity to

chemotherapy) in line 292 of the revised manuscript (namely, line 291 of the original manuscript).

Line 323 – How do you enhance maintenance?

Response: Thanks for the reviewer's comments and we apologize for the confusion. CSCs are resistant to standard chemotherapy, and we replaced the "enhancing" with "promoting" in line 324 of the revised manuscript (namely, line 323 of the original manuscript).

Lines 425 – 429 Given the range by which the different cell lines were impacted, this is a bold statement.

Response: Thanks for the reviewer's valuable comments. We modified the statement in lines 425 -429 in the original manuscript as "Our results indicated that high level of OTUD1 drives JNK pathway activation, suggesting that the OTUD1 might serve as biomarkers for JNK inhibition based OCSC-targeted therapy" in line 424-426 in the revised manuscript.

Lines 439 – 440 As presented the summary statement is not supported by the data provided and the conclusion should be toned down.

Response: We toned down the conclusion in lines 439-440 of the original manuscript as "In summary, our work suggests that aggresome formation in tumor cells could function as a signaling hub, moreover, aggresome based therapy has translational potential for patients with OTUD1^{high} ovarian cancer" in line 436-438 of the revised manuscript.

Line 574 -5 What are distal ovarian tissues? Please define.

Response: Thanks for pointing this out. The ovarian cancer tissue chip used in this study contains ovarian cancer samples and normal ovarian tissue samples. IHC results of serous adenocarcinoma were analyzed. We modified the description in lines 574-575 of the original manuscript as "Tissue chips containing with 4 cases of low grade ovarian serous carcinoma and 54 cases of high grade ovarian serous carcinoma" in lines 572-574 of the revised manuscript.

Are the supplementary figures 1 and 2 duplicate

Response: Thanks for pointing this out. We apologize for the error and we corrected it in revised manuscript.

Reviewer #2 (Remarks to the Author):

The authors have addressed all my concerns.

Reviewer #3 (Remarks to the Author):

Summary:

All the concerns raised were properly addressed in the rebuttal letter and the revised manuscript. There are some minor issues in the revised manuscript, listed as follows:

Remaining issues:

1. Line 29 in the Abstract: which is mediated by a short peptide locates in …" (located?)

Response: Thanks for pointing this out. We modified “locates” in line 29 of the original manuscript to “located” in line 30 of the revised manuscript.

2. Line 50-51 in the Introduction: “While the high grade serous ovarian cancer (HGSOC), which is the most the common histotype in the clinic, was believed to arise from the fallopian tube.” Gramatical error?

Response: Thanks for pointing this out. We modified the statement “While the high grade serous ovarian cancer (HGSOC), which is the most the common histotype in the clinic, was believed to arise from the fallopian tube” in lines 50-51 of original manuscript as “High-grade serous ovarian cancer (HGSOC), which is the most common histotype in the clinic, is believed to arise from the fallopian tube” in lines 49-50 of the revised manuscript.

3. Line 126-139 and the supplementary Fig. 1: It seems like there are mistakes in the figure labeling (all the labels are shifted alphabetically?)

Response: Thanks for pointing this out. We modified the figure labeling in lines 128-139 of the revised manuscript (namely, lines 126-139 of the revised manuscript).

4. Supplementary Fig 2 seems to be identical to supp. Fig. 1. Mistakes while uploading?

Response: Thanks for pointing this out. We apologize for the error and we corrected it in revised manuscript.

5. Line 205-206: “We then hypothesized that if the aggregation was an aggresome, which is normally formed relied on the presence of microtubule” (relying?)

Response: Thanks for pointing this out. We modified “relied” in lines 205-206 of original manuscript as “depending on” in line 207 of revised manuscript.

6. Line 240 and Supplementary Fig. 4A, these labels of the OTUD constructs are a bit confusing. aa 287-481 was termed N and aa 1-286 was termed C? maybe NΔ and CΔ respectively?

Response: Thanks for pointing this out. aa 1-286 was termed N and aa 287-481 was termed C. We modified labels of the OTUD constructs “Flag-OTUD1(C), HA-OTUD1(C), Flag-OTUD1(N), and HA-OTUD1(N)” in supplementary Fig. 4A to “Flag-OTUD1(ΔC), HA-OTUD1(ΔC), Flag-OTUD1(ΔN), and HA-OTUD1(ΔN)”.

7. Line 252: “, expression of the Δ105-164 (M1) but not OTUD1WT significantly reduced cellular OTUD1 aggresome formation and co-localized ASK1 aggregation.” This is a bit imprecise description to me. Maybe “ectopically expressed Δ105-164 (OTUD-M1) did not form aggresome colocalized with ASK1, as observed for OTUD1WT” or “deletion of aa 105-164 disrupted the tendency of OTUD1 to form aggresome colocalized with ASK1”.

Response: Thanks for the reviewer’s comments. We changed the statement “expression of the Δ105-164 (M1) but not OTUD1WT significantly reduced cellular OTUD1 aggresome formation and co-localized ASK1 aggregation” in line 252 of the original manuscript to “deletion of aa 105-164 disrupted the tendency of OTUD1 to form aggresomes colocalized with ASK1” in lines 253-254 of the revised manuscript.

8. Line 324 and supplementary Fig 7: is there a mistake in the fig indication? (Supplementary Fig. 7D rather then C?) To support the conclusion "OTUD1 expression reduced the sensitivity of SKOV3, OVCAR8 and CAOV3 cells to cisplatin treatment…", the author need to compare the OTUD1 expression + cisplatin (DPP) group to the empty vector (EV) + DPP control. However, I only saw EV + DPP in the statistic plot but not in the corresponding image of Supp. Fig. 7D.

Response: Thanks for pointing this out. We apologize for the mistake and changed "Supplementary Fig. 7C" to "Supplementary Fig. 7D" in line 325 of the revised manuscript. In addition, we modified the Supplementary Fig. 7D and the corresponding statistic plot in the revised manuscript.

References

1. Prasetyanti PR, Medema JP. Intra-tumor heterogeneity from a cancer stem cell perspective. *Mol Cancer* **16**, 41 (2017).
2. Keyvani V*, et al.* Ovarian cancer stem cells and targeted therapy. *J Ovarian Res* **12**, 120 (2019).
3. McAuliffe SM*, et al.* Targeting Notch, a key pathway for ovarian cancer stem cells, sensitizes tumors to platinum therapy. *Proc Natl Acad Sci U S A* **109**, E2939-E2948 (2012).
4. Batlle E, Clevers H. Cancer stem cells revisited. *Nat Med* **23**, 1124-1134 (2017).

Reviewers' Comments:

Reviewer #1:
Remarks to the Author:

Within the abstract on line 27 the authors state that
'the multiomics screen revealed that a high level of OTUD1 is important for retaining ovarian cancer stem properties. " The authors responded to the previous critiques raised by acknowledging that the high level of OTUD1 may only be important in some lines. More importantly, however, to be clear the multiomics screen did not reveal that a high level of OTUD1 is important it only set the stage to test its functional value in the subsequent experiments.
There remain some issues with the terminology around CSCs
Lines 33 and 36 highlight the language the authors use that cause confusion throughout the manuscript …… their use of "OCSC stemness maintenance" …. The authors should be consistent and use ….OSCS maintenance or support OSCs properties or enhance OSCS numbers etc… the term OCSC stemness is redundant. Again, in line 36 the authors claim they appreciate that tumorigenicity is a property of OSCS so why be redundant? Examples are too numerous to point out but some are highlighted below

Lines 86-87 the authors write that these findings revealed that aggresome formation contributes to the existence of cancer stem cells and plays an important role in tumorigenesis….. The way the sentence is presented they are implying the aggresomes contribute to the genesis of OCSCs? Because if you already have OCSCs you are not contributing to the genesis of new tumor if the tumor already exists. Or are they referring to progression or recurrent disease?

Lines 93 and 125 …..maintaining CSC stemness should just be maintaining CSCs

Line 138 …..implying that OTUD1 probably contributes to tumorigenesis through its effect on OCSC stemness …. Aside from the redundancy of OCSC stemness, again if you already have OCSC you already have tumor so it would not be tumorigenesis – it would be tumor progression or recurrence …

Line 142….. promotes OCSC stemness … should be promotes OCSC properties or maintenance

Lines 276 and 277 see comments stated above

Line 281 on the stemness of OCSCs ….?

Lines 305 – 306, 314, 324, 329 etc..

The list goes on

Below is our point-by-point response (in blue) to reviewer's comments (in black) for your consideration

Reviewer #1 (Remarks to the Author):

Within the abstract on line 27 the authors state that 'the multiomics screen revealed that a high level of OTUD1 is important for retaining ovarian cancer stem properties. " The authors responded to the previous critiques raised by acknowledging that the high level of OTUD1 may only be important in some lines. More importantly, however, to be clear the multiomics screen did not reveal that a high level of OTUD1 is important it only set the stage to test its functional value in the subsequent experiments.

Response: We appreciate reviewer's comment. To avoid any possible confusion, this sentence has been rephrased to read as follows "the multiomics screen revealed that OTUD1 protein plays an important role in retaining ovarian cancer stem cell (OCSC) properties."

There remain some issues with the terminology around CSCs

Lines 33 and 36 highlight the language the authors use that cause confusion throughout the manuscript …… their use of "OCSC stemness maintenance" …. The authors should be consistent and use ….OSCS maintenance or support OSCs properties or enhance OSCS numbers etc… the term OCSC stemness is redundant. Again, in line 36 the authors claim they appreciate that tumorigenicity is a property of OSCS so why be redundant? Examples are too numerous to point out but some are highlighted below

Response: Thanks for the reviewer's comments. According to reviewer's suggestion, we used the abbreviation "OSCS" in this revised manuscript to refer to "OCSC stemnes". We have changed "OCSC stemness maintenance" in line 33 of the manuscript to "OSCS maintenance". In addition, the sentence in line 36 of the manuscript has been changed to "effectively reduced OSCS of $OTUD1^{high}$ ovarian cancer cells".

Lines 86-87 the authors write that these findings revealed that aggresome formation contributes to the existence of cancer stem cells and plays an important role in tumorigenesis….. The way the sentence is presented they are implying the aggresomes contribute to the genesis of OCSCs? Because if you already have OCSCs you are not contributing to the genesis of new tumor if the tumor already exists. Or are they referring to progression or recurrent disease?

Response: We appreciate reviewer's comment. We have changed "tumorigenesis" to "cancer progression and recurrence" in line 87.

Lines 93 and 125 …..maintaining CSC stemness should just be maintaining CSCs

Response: We appreciate reviewer's comment. We have changed "CSC

stemness” to “OSCS” in line 93 and line 125, respectively.

Line 138 …..implying that OTUD1 probably contributes to tumorigenesis through its effect on OCSC stemness …. Aside from the redundancy of OCSC stemness, again if you already have OCSC you already have tumor so it would not be tumorigenesis – it would be tumor progression or recurrence …
Response: We agreed with reviewer’s suggestion. We have changed “tumorigenesis” to “cancer progression” in line 138.

Line 142….. promotes OCSC stemness … should be promotes OCSC properties or maintenance
Response: We have modified the sentences in line 142 according to reviewer’s comment.

Lines 276 and 277 see comments stated above
Response: We have rephrased the sentence in line 276 of the manuscript as follows “Abrogation of OTUD1 aggresome formation impaired the stimulatory effect of OTUD1 on OSCS maintenance”.

Line 281 on the stemness of OCSCs ….?
Response: We have changed the “stemness of OCSCs” to “OSCS” in line 281 of the revised manuscript.

Lines 305 – 306, 314, 324, 329 etc..
Response: We appreciate reviewer’s comment. We have changed the “OCSC stemness” in lines 305-306, 314, 324, 329 of the manuscript to “OSCS”.
